# Supplementary material for: Risk-adjusted trend in national inpatient fall rates observed from 2011 to 2019 in acute care hospitals in Switzerland: a repeated multicentre cross-sectional study
Source: BMJ Open. 2024 May 15;14(5):e082417. doi: 10.1136/bmjopen-2023-082417 (PMC11097859; doi:10.1136/bmjopen-2023-082417)
Supplement: Supplementary data [file bmjopen-2023-082417supp001.pdf]

Supplementary file

			2011	2012	2013	2014	2015	2016	2017	2018	2019
**BFH national analysis dataset**	*Survey year* / *n=*		15637	18105	17486	17550	17233	17619	17438	17250	17464
**Exclusion**	Non-comparable hospital/ward types	*n=*	42	-	-	-	-	-	-	-	-
	Patients younger than 18 years	*n=*	71	-	-	-	-	-	-	-	-
	Conflicting cases: Non-participation, but data available; participation, but missings on mandatory variables	*n=*	44	6	3	1	-	-	-	-	-
	Unknown on outcome variable	*n=*	172	203	209	167	158	11	41	78	71
	Non-participation	*n=*	4939	4453	4580	4233	4070	4154	4211	4023	4224
	No data available on care dependency*	*n=*	4726	-	-	-	-	-	-	-	-
**Study population**		*n=*	5643	13443	12694	13149	13005	13454	13186	13149	13169
		%	36.1%	74.3%	72.6%	74.9%	75.5%	76.4%	75.6%	76.2%	75.4%
**Merged analysis dataset**		*n=*					110892				
		%					71.2%				

*In 2011, the hospitals were free to decide whether to collect the Care Dependency Scale (CDS). In the analyses, 4726 patients from the survey year 2011 therefore had to be excluded due to missing values. No significant group difference was found in the frequency of inpatient falls in the excluded group (no information on CDS available) compared to the included group (information on CDS available) (Chi-Square *p*=0.107).

Supplementary figure S1: Flowchart depicting the selection of cases for analysis

Supplementary table S1: Description of patient characteristics in the sample across the survey years

	**2011**	**2012**	**2013**	**2014**	**2015**	**2016**	**2017**	**2018**	**2019**	**Total**
	n = 5643	*n* = 13443	*n* = 12694	*n* = 13149	*n* = 13005	*n* = 13454	*n* = 13186	*n* = 13149	*n* = 13169	*n* = 110892
**Patient characteristics**	*n* (%)	*n* (%)	*n* (%)	*n* (%)	*n* (%)	*n* (%)	*n* (%)	*n* (%)	*n* (%)	*n* (%)
Sex [female]	3000 (53.2)	7300 (54.3)	6400 (50.4)	6693 (50.9)	6519 (50.1)	6646 (49.4)	6527 (49.5)	6458 (49.1)	6471 (49.1)	56014 (50.5)
Care dependency (CDS)										
care independent [70-75]	3106 (55.0)	7533 (56.0)	6790 (53.5)	7182 (54.6)	7080 (54.4)	7340 (54.6)	7036 (53.4)	7000 (53.2)	6807 (51.7)	59874 (54.0)
to a great extent independent [60-69]	1288 (22.8)	2893 (21.5)	2963 (23.3)	2989 (22.7)	3072 (23.6)	3236 (24.1)	3130 (23.7)	3174 (24.1)	3225 (24.5)	25970 (23.4)
partially dependent [45-59]	707 (12.5)	1844 (13.7)	1813 (14.3)	1879 (14.3)	1767 (13.6)	1760 (13.1)	1890 (14.3)	1888 (14.4)	1960 (14.9)	15508 (14.0)
to a great extent dependent [25-44]	408 (7.2)	883 (6.6)	850 (6.7)	814 (6.2)	783 (6.0)	776 (5.8)	817 (6.2)	792 (6.0)	869 (6.6)	6992 (6.3)
completely dependent [15-24]	134 (2.4)	290 (2.2)	278 (2.2)	285 (2.2)	303 (2.3)	342 (2.5)	313 (2.4)	295 (2.2)	308 (2.3)	2548 (2.3)
Surgical procedure within 14 days prior to measurement [yes]	2111 (37.4)	5336 (39.7)	5218 (41.1)	5469 (41.6)	5708 (43.9)	5981 (44.5)	5863 (44.5)	5774 (43.9)	5773 (43.8)	47233 (42.6)
ICD-10 – Diseases of the circulatory system [yes]	2949 (52.3)	6607 (49.2)	6453 (50.8)	6622 (50.4)	6755 (51.9)	7275 (54.1)	7368 (55.9)	7397 (56.3)	7560 (57.4)	58986 (53.2)
ICD-10 – Diseases of the musculoskeletal system and connective tissue [yes]	1876 (33.2)	4317 (32.1)	4632 (36.5)	4919 (37.4)	5041 (38.8)	5550 (41.3)	5373 (40.7)	5357 (40.7)	5284 (40.1)	42349 (38.2)
ICD-10 – Endocrine, nutritional and metabolic diseases [yes]	1476 (26.2)	3277 (24.4)	3569 (28.1)	3788 (28.8)	4047 (31.1)	4359 (32.4)	4522 (34.3)	4430 (33.7)	4776 (36.3)	34244 (30.9)
ICD-10 – Diseases of the genitourinary system [yes]	1578 (28.0)	3519 (26.2)	3387 (26.7)	3522 (26.8)	3509 (27.0)	4086 (30.4)	4085 (31.0)	4028 (30.6)	4365 (33.1)	32079 (28.9)
ICD-10 – Diseases of the digestive system [yes]	1538 (27.3)	3191 (23.7)	2991 (23.6)	3155 (24.0)	3096 (23.8)	3417 (25.4)	3443 (26.1)	3385 (25.7)	3668 (27.9)	27884 (25.1)
ICD-10 – Diseases of the respiratory system [yes]	1175 (20.8)	2592 (19.3)	2805 (22.1)	2837 (21.6)	3038 (23.4)	3259 (24.2)	3241 (24.6)	3181 (24.2)	3472 (26.4)	25600 (23.1)
ICD-10 – Neoplasms [yes]	1014 (18.0)	2204 (16.4)	1739 (13.7)	2426 (18.5)	2450 (18.8)	2716 (20.2)	2836 (21.5)	2792 (21.2)	2990 (22.7)	21167 (19.1)
ICD-10 – Mental, behavioural and neurodevelopmental disorders [yes]	878 (15.6)	2019 (15.0)	2222 (17.5)	2196 (16.7)	2182 (16.8)	2485 (18.5)	2609 (19.8)	2531 (19.2)	2705 (20.5)	19827 (17.9)
ICD-10 – Diseases of the blood and blood-forming organs [yes]	494 (8.8)	1089 (8.1)	1663 (13.1)	1739 (13.2)	1785 (13.7)	2083 (15.5)	2145 (16.3)	2092 (15.9)	2371 (18.0)	15461 (13.9)
ICD-10 – Diseases of the nervous system [yes]	465 (8.2)	1139 (8.5)	1333 (10.5)	1598 (12.2)	1496 (11.5)	1813 (13.5)	1897 (14.4)	1823 (13.9)	1930 (14.7)	13494 (12.2)
ICD-10 – Certain infectious and parasitic diseases [yes]	752 (13.3)	1672 (12.4)	1325 (10.4)	1365 (10.4)	1386 (10.7)	1695 (12.6)	1823 (13.8)	1663 (12.6)	1813 (13.8)	13494 (12.2)
ICD-10 – Diseases of the eye and adnexa OR ICD-10 – Diseases of the ear and mastoid process [yes]	390 (6.9)	745 (5.5)	871 (6.9)	904 (6.9)	919 (7.1)	1046 (7.8)	1091 (8.3)	1046 (8.0)	1136 (8.6)	8148 (7.3)
ICD-10 – Diseases of the skin and subcutaneous tissue [yes]	272 (4.8)	603 (4.5)	812 (6.4)	848 (6.4)	909 (7.0)	988 (7.3)	1031 (7.8)	1021 (7.8)	1088 (8.3)	7572 (6.8)
ICD-10 – Injury, poisoning and certain other consequences of external causes [yes]	631 (11.2)	1207 (9.0)	922 (7.3)	802 (6.1)	762 (5.9)	808 (6.0)	708 (5.4)	875 (6.7)	844 (6.4)	7559 (6.8)
ICD-10 – Congenital malformations, deformations and chromosomal abnormalities [yes]	34 (0.6)	72 (0.5)	82 (0.6)	67 (0.5)	77 (0.6)	70 (0.5)	64 (0.5)	81 (0.6)	78 (0.6)	625 (0.6)
	M (IQR)	M (IQR)	M (IQR)	M (IQR)	M (IQR)	M (IQR)	M (IQR)	M (IQR)	M (IQR)	M (IQR)
Age [in years]	69 (25)	68 (27)	69 (24)	69 (24)	69 (23)	70 (24)	70 (24)	70 (23)	71 (23)	70 (24)

M=Median; IQR=interquartile range.

Bernet NS, *et al. BMJ Open* 2024; 14:e082417. doi: 10.1136/bmjopen-2023-082417

Supplementary table S2: Illustration of the estimated non-linear trend using odds ratios, each of which describes the decrease in Swiss national fall rates in relation to the previous survey year

**Survey year**	**2011/2012**	**2012/2013**	**2013/2014**	**2014/2015**	**2015/2016**	**2016/2017**	**2017/2018**	**2018/2019**
OR	0.92	0.93	0.94	0.95	0.97	0.98	0.99	1.00
95% *CI*	0.88 – 0.96	0.90 – 0.96	0.92 – 0.96	0.94 – 0.97	0.95 – 0.98	0.96 – 1.00	0.96 – 1.02	0.96 – 1.04

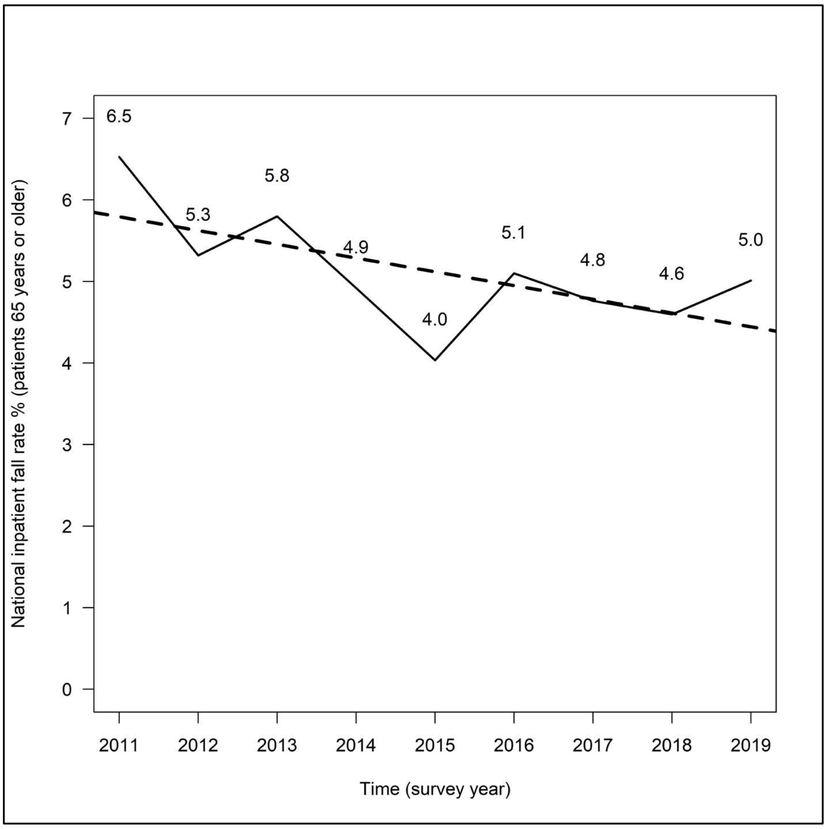

Supplementary figure S2: Visualisation of the descriptive Swiss national inpatient fall rates from 2011 to 2019 and the estimated linear trend over time, taking into account only patients 65 years old or older

Supplementary table S3: Overview of the two-level random intercept logistic regression model used to derive the risk-adjusted trend in Swiss national inpatient fall rates, taking into account only patients 65 years old or older

	**Risk-adjusted trend in national inpatient fall rates**				
Predictors	*coeff.*	*SE*	*p-value*	*OR*	*95% CI*
(Intercept)	-4.52	0.21	**<0.001**	-	-
**Time related factors (trend)**					
Time	-0.12	0.04	**0.001**	-	-
(Time)^2	0.01	0.00	**0.020**	-	-
**Patient related fall risk factors**					
Age [in years]	0.01	0.00	**<0.001**	1.01	1.01 – 1.02
CDS [care independent (70-75)]	Ref.				
CDS [to a great extent independent (60-69)]	0.97	0.06	**<0.001**	2.64	2.36 – 2.95
CDS [partially dependent (45-59)]	1.38	0.06	**<0.001**	3.96	3.54 – 4.43
CDS [to a great extent dependent (25-44)]	1.66	0.06	**<0.001**	5.25	4.63 – 5.94
CDS [completely dependent (15-24)]	1.30	0.09	**<0.001**	3.68	3.05 – 4.43
ICD-10 – Mental and Behavioural disorders [yes]	0.53	0.04	**<0.001**	1.71	1.58 – 1.85
ICD-10 – Neoplasms [yes]	0.32	0.04	**<0.001**	1.38	1.27 – 1.50
ICD-10 – Diseases of the nervous system [yes]	0.21	0.05	**<0.001**	1.24	1.13 – 1.36
ICD-10 – Diseases of the blood and blood-forming organs [yes]	0.21	0.04	**<0.001**	1.24	1.13 – 1.35
ICD-10 – Injury, poisoning, other consequences of external causes [yes]	0.18	0.06	**0.003**	1.20	1.06 – 1.36
ICD-10 – Endocrine, nutritional and metabolic diseases [yes]	0.09	0.04	**0.015**	1.09	1.02 – 1.18
Sex [female]	-0.27	0.04	**<0.001**	0.76	0.71 – 0.82
Surgical procedure within 14 days prior to measurement [yes]	-0.40	0.04	**<0.001**	0.67	0.62 – 0.73
**Random Effects**					
τ_{00} [variability in hospital intercepts]			0.09		
N [hospitals]			221		
Observations			67336		

Significant *p*-values are highlighted in bold.

Bernet NS, *et al. BMJ Open* 2024; 14:e082417. doi: 10.1136/bmjopen-2023-082417

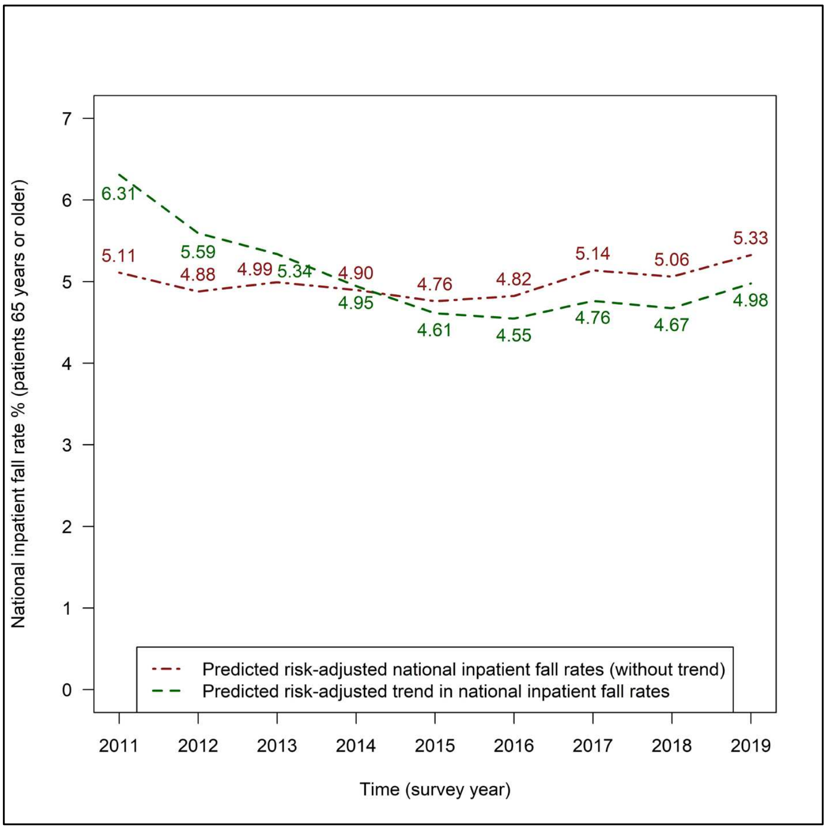

Supplementary figure S3: Visualisation of predicted risk-adjusted (with and without time trend) Swiss national inpatient fall rates from 2011 to 2019, taking into account only patients 65 years old or older

Supplementary table S4: Illustration of the estimated non-linear trend using odds ratios, each of which describes the decrease in Swiss national fall rates in relation to the previous survey year, taking into account only patients 65 years old or older

Survey year	2011/2012	2012/2013	2013/2014	2014/2015	2015/2016	2016/2017	2017/2018	2018/2019
OR	0.91	0.92	0.94	0.95	0.97	0.98	1.00	1.01
**95% *CI***	0.86 – 0.96	0.89 – 0.96	0.91 – 0.96	0.93 – 0.97	0.95 – 0.98	0.96 – 1.00	0.96 – 1.03	0.97 – 1.06

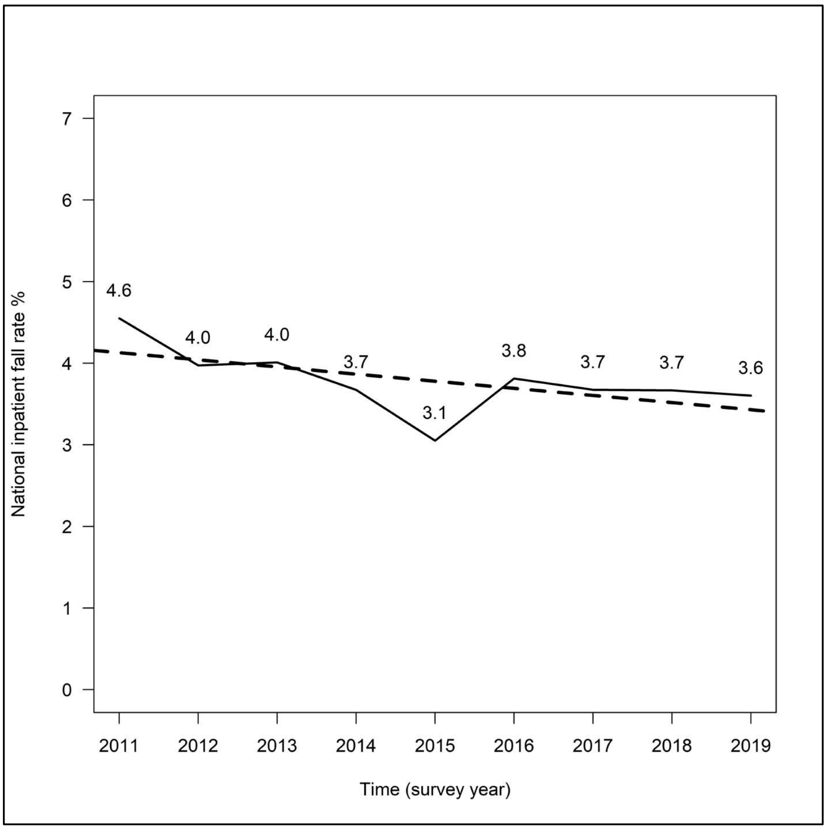

Supplementary figure S4: Visualisation of the descriptive Swiss national inpatient fall rates from 2011 to 2019 and the estimated linear trend over time, taking into account only hospitals that provided data in all survey years

Supplementary table S5: Overview of the two-level random intercept logistic regression model used to derive the risk-adjusted trend in Swiss national inpatient fall rates, taking into account only hospitals that provided data in all survey years

	Risk-adjusted trend in national inpatient fall rates				
Predictors	*coeff.*	*SE*	*p-value*	*OR*	*95% CI*
(Intercept)	-5.16	0.13	**<0.001**	-	-
**Time related factors (trend)**					
Time	-0.05	0.01	**<0.001**	-	-
**Patient related fall risk factors**					
Age [in years]	0.01	0.00	**<0.001**	1.02	1.01 – 1.02
CDS [care independent (70-75)]	Ref.				
CDS [to a great extent independent (60-69)]	1.04	0.06	**<0.001**	2.82	2.49 – 3.20
CDS [partially dependent (45-59)]	1.45	0.07	**<0.001**	4.25	3.73 – 4.84
CDS [to a great extent dependent (25-44)]	1.75	0.07	**<0.001**	5.76	4.99 – 6.66
CDS [completely dependent (15-24)]	1.28	0.11	**<0.001**	3.59	2.88 – 4.46
ICD-10 – Mental and Behavioural disorders [yes]	0.58	0.05	**<0.001**	1.79	1.64 – 1.96
ICD-10 – Neoplasms [yes]	0.35	0.05	**<0.001**	1.42	1.29 – 1.56
ICD-10 – Diseases of the nervous system [yes]	0.26	0.06	**<0.001**	1.30	1.16 – 1.45
ICD-10 – Diseases of the blood and blood-forming organs [yes]	0.22	0.05	**<0.001**	1.24	1.12 – 1.38
ICD-10 – Endocrine, nutritional and metabolic diseases [yes]	0.11	0.04	**0.011**	1.12	1.03 – 1.22
ICD-10 – Diseases of the circulatory system [yes]	0.12	0.05	**0.021**	1.12	1.02 – 1.24
Sex [female]	-0.17	0.04	**<0.001**	0.85	0.78 – 0.92
Surgical procedure within 14 days prior to measurement [yes]	-0.50	0.05	**<0.001**	0.60	0.55 – 0.67
**Random Effects**					
τ_{00} [variability in hospital intercepts]			0.06		
N [hospitals]			93		
Observations			64038		

Significant *p*-values are highlighted in bold.

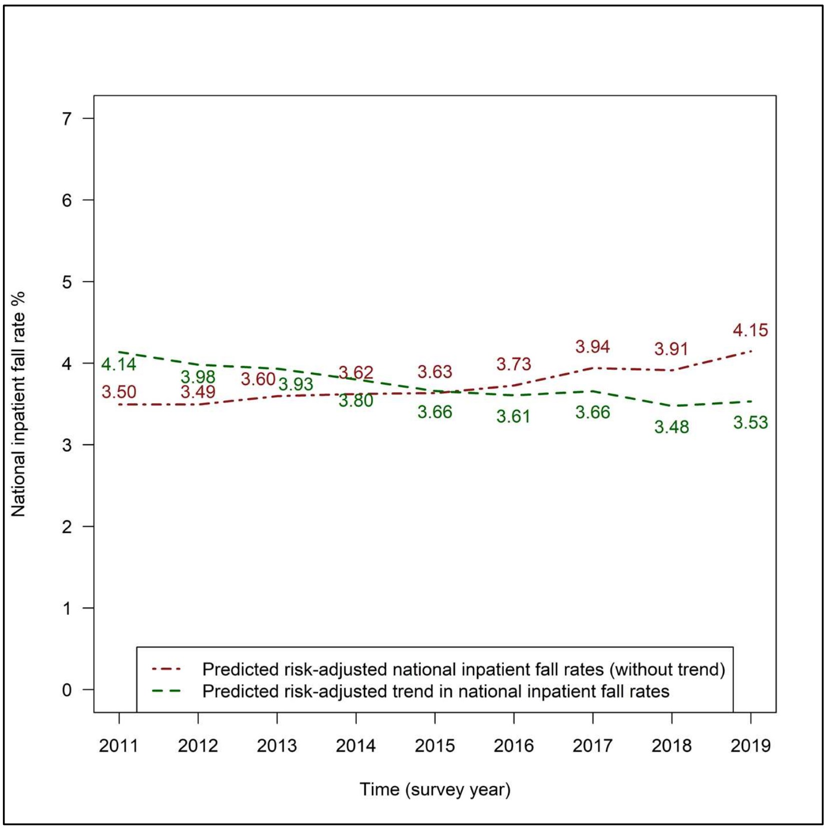

Supplementary figure S5: Visualisation of predicted risk-adjusted (with and without time trend) Swiss national inpatient fall rates from 2011 to 2019, taking into account only hospitals that provided complete data in all survey years

Bernet NS, *et al. BMJ Open* 2024; 14:e082417. doi: 10.1136/bmjopen-2023-082417
