# Supplementary material for: Risk-adjusted trend in national inpatient fall rates observed from 2011 to 2019 in acute care hospitals in Switzerland: a repeated multicentre cross-sectional study
Source: BMJ Open. 2024 May 15;14(5):e082417. doi: 10.1136/bmjopen-2023-082417 (PMC11097859; doi:10.1136/bmjopen-2023-082417)
Supplement: Author's manuscript [file bmjopen-2023-082417.draft_revisions.pdf]

BMJ Open

BMJ Open is committed to open peer review. As part of this commitment we make the peer review history of every article we publish publicly available.

When an article is published we post the peer reviewers' comments and the authors' responses online. We also post the versions of the paper that were used during peer review. These are the versions that the peer review comments apply to.

The versions of the paper that follow are the versions that were submitted during the peer review process. They are not the versions of record or the final published versions. They should not be cited or distributed as the published version of this manuscript.

BMJ Open is an open access journal and the full, final, typeset and author-corrected version of record of the manuscript is available on our site with no access controls, subscription charges or pay-per-view fees (http://bmjopen.bmj.com).

If you have any questions on BMJ Open's open peer review process please email info.bmjopen@bmj.com

# Risk-adjusted trend in national inpatient fall rates observed from 2011 to 2019 in acute care hospitals in Switzerland: a repeated multicentre cross-sectional study

| | |
|---|---|
| Journal: | *BMJ Open* |
| Manuscript ID | bmjopen-2023-082417 |
| Article Type: | Original research |
| Date Submitted by the Author: | 22-Nov-2023 |
| Complete List of Authors: | Bernet, Niklaus; Bern University of Applied Sciences, School of Health Professions<br>Everink, Irma; Maastricht University Care and Public Health Research Institute, Department of Health Services Research<br>Hahn, Sabine; Bern University of Applied Sciences, School of Health Professions<br>Müller, Marianne; Bern University of Applied Sciences, School of Health Professions<br>Schols, Jos; Maastricht University Care and Public Health Research Institute, Department of Health Services Research |
| Keywords: | Hospitals, Nursing Care, Quality in health care < HEALTH SERVICES ADMINISTRATION & MANAGEMENT, Risk Factors |
| | |

SCHOLARONE™
Manuscripts

*I, the Submitting Author has the right to grant and does grant on behalf of all authors of the Work (as defined in the below author licence), an exclusive licence and/or a non-exclusive licence for contributions from authors who are: i) UK Crown employees; ii) where BMJ has agreed a CC-BY licence shall apply, and/or iii) in accordance with the terms applicable for US Federal Government officers or employees acting as part of their official duties; on a worldwide, perpetual, irrevocable, royalty-free basis to BMJ Publishing Group Ltd ("BMJ") its licensees and where the relevant Journal is co-owned by BMJ to the co-owners of the Journal, to publish the Work in this journal and any other BMJ products and to exploit all rights, as set out in our licence.*

*The Submitting Author accepts and understands that any supply made under these terms is made by BMJ to the Submitting Author unless you are acting as an employee on behalf of your employer or a postgraduate student of an affiliated institution which is paying any applicable article publishing charge ("APC") for Open Access articles. Where the Submitting Author wishes to make the Work available on an Open Access basis (and intends to pay the relevant APC), the terms of reuse of such Open Access shall be governed by a Creative Commons licence – details of these licences and which Creative Commons licence will apply to this Work are set out in our licence referred to above.*

*Other than as permitted in any relevant BMJ Author's Self Archiving Policies, I confirm this Work has not been accepted for publication elsewhere, is not being considered for publication elsewhere and does not duplicate material already published. I confirm all authors consent to publication of this Work and authorise the granting of this licence.*

For peer review only

# Risk-adjusted trend in national inpatient fall rates observed from 2011 to 2019 in acute care hospitals in Switzerland: a repeated multicentre cross-sectional study

**Author names:**

Niklaus S Bernet[a], Irma HJ Everink[b], Sabine Hahn[a], Marianne Müller[a], Jos MGA Schols[b]

**Affiliations**:

[a] Bern University of Applied Sciences, School of Health Professions, Applied Research & Development in Nursing, Murtenstrasse 10, 3008 Bern, Switzerland

niklausstefan.bernet@bfh.ch, sabine.hahn@bfh.ch, marianne.mueller@bfh.ch

[b] Maastricht University, Care and Public Health Research Institute, Department of Health Services Research, PO BOX 616, 6200 MD, Maastricht, the Netherlands

i.everink@maastrichtuniversity.nl, jos.schols@maastrichtuniversity.nl

**Corresponding author**:

Niklaus S Bernet, Bern University of Applied Sciences, School of Health Professions, Applied Research & Development in Nursing, Murtenstrasse 10, 3008 Bern, Switzerland, +41318484585, niklausstefan.bernet@bfh.ch

Word count of the main manuscript (introduction, methods, results, discussion, conclusions): 4488



For peer review only - http://bmjopen.bmj.com/site/about/guidelines.xhtml

## Abstract

*Objectives:* This study aimed to investigate whether a significant trend regarding inpatient falls in Swiss acute hospitals could be confirmed on a national level between 2011 and 2019, and whether the trend persists after risk adjustment for patient-related fall risk factors.

*Design:* A secondary data analysis was conducted based on annual multicentre cross-sectional studies carried out between 2011 and 2019.

*Setting:* All Swiss acute care hospitals were obliged to participate in the surveys. Except for emergency departments, outpatient wards and recovery rooms, all wards were included.

*Participants:* All inpatients aged 18 or older who had given their informed consent were included in the surveys.

*Main outcome measure:* Whether a patient had fallen in hospital was retrospectively determined on the survey day by asking patients the following question: Have you fallen in this institution in the last 30 days?

*Results:* Based on data from 110893 patients from 222 Swiss hospitals, a national inpatient fall rate of 3.7% was determined over the 9 survey years. A significant linear decreasing trend (*p* for trend = 0.004) was observed using the Cochran-Armitage trend test. After adjusting for patient-related fall risk factors in a multilevel logistic model, a significant decreasing curvilinear trend was found at the national level.

*Conclusions:* A significant decrease in fall rates in Swiss hospitals, indicating an improvement in the quality of care provided, could be confirmed both descriptively



For peer review only - http://bmjopen.bmj.com/site/about/guidelines.xhtml

and after risk adjustment. However, the curvilinear trend, meaning that the decreasing trend found flattens out over time, also indicates a possible future increase in fall rates. Monitoring of falls in hospitals should be maintained at national level. Risk adjustment accounts for the observed increase in patient-related fall risk factors in hospitals, thus promoting a fairer comparison of quality of care provided over time.

# Article summary

### Strengths and limitations of this study

- The data used in this study are based on an internationally well-defined and standardised primary data collection, which favours high data quality in terms of completeness and uniformity. Therefore, this data base may have relevant advantages compared to the use of data generated for other purposes (e.g. a critical incidence reporting system).
- The study is based on a nationally representative sample in which almost all Swiss hospitals with an acute somatic care mandate were obliged to participate in the annual survey between 2011 and 2019. Therefore, the results found can be generalised to the situation in Switzerland.
- The data used in our study enabled a comparison of fall rates over time, taking into account patient-related fall risk factors. The risk adjustment method used contributes to a fairer comparison of results over time.
- In principle, risk adjustment should only consider differences in patients' risk factors before or at the time of admission. This means that adjustment should only be made for risk factors that patients have already brought with them to hospital and which are therefore not the product of care or influenced by care



For peer review only - http://bmjopen.bmj.com/site/about/guidelines.xhtml

during hospitalisation. Due to the annual cross-sectional survey design, it is not possible in our study to determine the temporal sequence between risk factor and fall event and consequently to take it into account.


## Keywords

- Accidental falls
- Cross-Sectional Studies
- Hospitals
- Inpatients
- Quality Assurance
- Quality of Health Care
- Multilevel Analysis
- Risk Adjustment
- Trends




For peer review only - http://bmjopen.bmj.com/site/about/guidelines.xhtml

# Introduction

In health care, national quality measurements are a recognised strategy for monitoring and improving the quality of care.[1] They generate data that can be used at all levels of health care: at the national level for accountability and at the provider level for external as well as internal comparison.[2] There is evidence in the literature that regular quality measurements combined with effective change management can positively influence quality of care over time.[1 3] For example, in the US, a risk-adjusted trend analysis showed a significant reduction in adverse events in hospitals between 2010 and 2019.[4]

Falls in hospital are just such an adverse event, as they have serious consequences for both patients and the healthcare system as a whole.[5 6] Based on data from Australia, a fall in hospital results in an average of an 8-day longer hospital stay and an additional cost of $6669.[7] Many inpatient falls, though not all, can be prevented with rigorous application of best-practice prevention measures.[8] Therefore, the number of falls in hospital is widely recognised as a relevant indicator of quality of care. Internationally, there are only a few organisations that we are aware of, such as the Press Ganey National Database of Nursing Quality Indicators (NDNQI)[9 10] or the International Prevalence Measurement of Care Quality (LPZ)[11 12], that provide a national infrastructure for collecting data on the quality indicator of falls in hospital. Inpatient falls in Switzerland are systematically recorded using the LPZ method and submitted to the Swiss National Association for Quality Development in Hospitals and Clinics (ANQ). Since 2011, the inpatient fall rate has been surveyed annually in all acute care hospitals as part of a repeated cross-sectional study.[13]



For peer review only - http://bmjopen.bmj.com/site/about/guidelines.xhtml

A recently submitted descriptive analysis of inpatient fall rates based on data from Swiss hospitals between 2011 and 2019 showed that national rates stagnated over time after a substantial decrease following the first survey, although this could not be conclusively assessed from the descriptive analysis.[14] A decreasing trend in fall rates at the national level might be an indication that the quality of care in hospitals with regard to fall prevention has improved over time. However, a stagnation of fall rates over time would contradict the hypothesis that regular quality measurements favour the initiation of change processes and thus continuous quality improvement, which would be reflected in decreasing national inpatient fall rates over time. It is possible, however, that there are other reasons that favour a stagnation of fall rates in the hospitals and thus possibly mask the effect of regular quality measurements on quality improvement in the hospitals. First, not all falls are preventable[8] and, accordingly, a reduction to zero is unrealistic, so fall rates will remain constant at a certain level even if prevention is steadily improved. Second, the stagnation could suggest that a continuous intensification of prevention efforts to reduce falls in hospitals is no longer possible due to increasing financial constraints and staff shortages.[15] Third, the fall risk profiles of patients in hospital have changed over time, e.g. more elderly and multimorbid patients in hospital, which increases the chance of falling in hospital over time, thus counteracting a continuous reduction of fall rates in hospital despite continuous quality improvement.[14] In the last scenario, keeping fall rates constant would still be a success.

In order to at least take this aspect into account when comparing fall rates over time, it is usually recommended that changing patient-related risk factors should be controlled for, i.e. that a risk-adjusted comparison over time should be performed.[16] Risk adjustment in this context means that a statistical model is used to control for



For peer review only - http://bmjopen.bmj.com/site/about/guidelines.xhtml

ongoing changes in patient-related risk factors that cannot be influenced by hospitals. By keeping patient-related risk factors constant over time, it can be ensured that positive/negative trends are actually due to quality improvements/deteriorations in care rather than to changes in patient-related risk factors.

The objectives of this study were therefore, first, to investigate whether a trend regarding the prevalence of inpatient falls in acute care hospitals can be statistically confirmed at the national level in Switzerland between 2011 and 2019. Second, whether any trend identified would persist after risk adjustment for patient-related fall risk factors had been carried out.

# Methods

## Study design

We conducted a secondary data analysis of a multicentre cross-sectional study repeated annually between 2011 and 2019.

## Data collection and population

The data used on inpatient falls in Switzerland were collected within the framework of the International Prevalence Measurement of Care Quality (LPZ).[12] The method of the so-called LPZ measurement is described in detail elsewhere.[12 13 17] In short, as part of the LPZ measurement, data on various quality indicators such as falls, pressure ulcers and malnutrition are collected at bedside in different countries (e.g. the Netherlands, Austria, Switzerland) during a defined data collection period. In Switzerland, usually on the Tuesday of the second week in November.



For peer review only - http://bmjopen.bmj.com/site/about/guidelines.xhtml

For data collection, the LPZ questionnaire was used.[12 13] This questionnaire emerged from an instrument developed on the basis of a literature review, a Delphi and a pilot study for the cross-setting and cross-institutional survey of pressure injuries [18], which was assessed as reliable and valid.[18-20] Subsequently, the initial instrument was supplemented by additional instruments to capture further quality indicators, such as falls, by an international research group and based on evidence-based research. The LPZ questionnaire is divided into three parts: an institutional, a ward and a patient questionnaire. In 2016, the LPZ questionnaire was revised and has since been known as LPZ 2.0.

Data collection in hospitals is organised and coordinated by designated hospital coordinators who are trained during annual national training events using the train-the-trainer principle, so that they can subsequently train their clinical data collection teams on site. To additionally ensure uniform data collection, a detailed measurement manual with definitions, instructions, explanations and examples is available to all persons involved. The clinical data collection teams consist of two nurses who gather the required data directly from the patient or, where permissible, from the patient's documentation on the survey day and then enter it into the web-based data entry programme.

In Switzerland, all acute care hospitals with an acute somatic service mandate have been obliged to collect data on pressure injuries and inpatient falls once a year in November since 2011. In 2020 and 2021, no survey took place in Switzerland due to the Covid pandemic. Hospitals that did not have an acute somatic service mandate, such as psychiatric, rehabilitation and geriatric clinics, were excluded from the survey. At ward level, all wards were included, with the exception of emergency departments, outpatient wards and recovery rooms as of 2012, and additionally,



For peer review only - http://bmjopen.bmj.com/site/about/guidelines.xhtml

maternity wards as of 2013. On the survey day, all inpatients 18 years or older were included in the study. In addition, written consent had to be obtained from patients or legal representatives for inclusion in 2011, and verbal consent from 2012 onwards. The hospital coordinators were responsible for ensuring that all patients or, if they were unable to do so, their legal representatives were informed in advance in writing about the aim and procedure of the measurement. The written/oral consent of the patients was clarified directly at the time of the survey, that of the legal representatives in advance.

**Measures**

In the present study, selected data from the patient questionnaire on patient characteristics and falls of the LPZ questionnaire were used. Only variables that were collected on all survey dates and that had not undergone any significant changes in content over the course of the survey years were considered, although in some cases minor linguistic changes were accepted. The outcome variable "inpatient fall" was retrospectively assessed by the clinical data collection teams by answering (yes/no) the question "Has the client fallen in the last 30 days in this institution?". To answer the question, the patients were asked in the first instance, the nurse in charge in the second instance, and the patient documentation was reviewed in the third instance. A fall in hospital is defined in the LPZ measurement as follows: "Any unintentional change in position that results in the client coming to rest on the ground or other lower level, regardless of the reason".[12 21] The following patient-related characteristics were available as possible variables for risk adjustment: age, sex, surgical procedure within 14 days prior to measurement day, care dependency according to the Care Dependency Scale (CDS), ICD-10 (International Statistical Classification of Diseases and Related Health Problems 10$^{th}$



For peer review only - http://bmjopen.bmj.com/site/about/guidelines.xhtml

Revision[22]) diagnosis groups. For more information on the variables used in this study, see table 1.

*Table 1: Overview of the variables used in this study*

| **Outcome variable** | **Answer options** |
|---|---|
| Has the client fallen in the last 30 days in this institution | no [0] / yes [1] |
| **Patient-related variables** | **Answer options** |
| Age (in years) | scale |
| Sex | male [0] / female [1] |
| Surgical procedure within 14 days prior to measurement | no [0] / yes [1] |
| Care Dependency Scale (CDS)* | care independent (70–75) [0]<br>to a great extent independent (60–69) [1]<br>partially dependent (45–59) [2]<br>to a great extent dependent (25–44) [3]<br>completely dependent (15–24) [4] |
| ICD-10 – Diseases of the circulatory system | no [0] / yes [1] |
| ICD-10 – Diseases of the musculoskeletal system and connective tissue | no [0] / yes [1] |
| ICD-10 – Endocrine, nutritional and metabolic diseases | no [0] / yes [1] |
| ICD-10 – Diseases of the genitourinary system | no [0] / yes [1] |
| ICD-10 – Diseases of the digestive system | no [0] / yes [1] |
| ICD-10 – Diseases of the respiratory system | no [0] / yes [1] |
| ICD-10 – Neoplasms | no [0] / yes [1] |
| ICD-10 – Mental, behavioural and neurodevelopmental disorders | no [0] / yes [1] |
| ICD-10 – Diseases of the blood and blood-forming organs | no [0] / yes [1] |
| ICD-10 – Diseases of the nervous system | no [0] / yes [1] |
| ICD-10 – Certain infectious and parasitic diseases | no [0] / yes [1] |
| ICD-10 – Diseases of the skin and subcutaneous tissue | no [0] / yes [1] |
| ICD-10 – Injury, poisoning and certain other consequences of external causes | no [0] / yes [1] |
| ICD-10 – Diseases of the eye and adnexa AND ICD-10 – Diseases of the ear and mastoid process** | no [0] / yes [1] |
| ICD-10 – Congenital malformations, deformations and chromosomal abnormalities | no [0] / yes [1] |
| **Time variable** | **Values** |
| Time (survey year) | 2011 [1] / 2012 [2] / 2013 [3] / 2014 [4] / 2015 [5] / 2016 [6] / 2017 [7] / 2018 [8] / 2019 [9] |

* The Care Dependency Scale (CDS) is composed of 15 questions, which are rated on a scale from 1 to 5. The total score ranges from 15 to 75 points, whereby a lower score indicates higher care dependency.[23 24] The following five categories can be derived from the sum score: completely dependent on care from others, to a great extent dependent, partially dependent, to a great extent independent and care independent.[25 26] In 2011, the hospitals were free to decide whether to collect the CDS. In the analyses, 4726 patients from the survey year 2011 therefore had to be excluded due to missing values.

** These ICD-10 diagnosis groups were only recorded separately from 2013 onwards and were therefore combined for the analyses in order to standardise them.

## Data analysis



For peer review only - http://bmjopen.bmj.com/site/about/guidelines.xhtml

Once the nine datasets (one for each survey year) were uniformly prepared and cleaned, the datasets were merged into one overall dataset by adding a time variable. Then, the sample was first described by means of frequencies and percentages and age by means of median and interquartile range (IQR). In addition, the annual fall rates from 2011 to 2019 were calculated by dividing the annual number of patients with a fall in hospital by the number of all participating patients and multiplying by 100.

Second, to investigate whether a trend in the prevalence of inpatient falls in acute care hospitals could be statistically confirmed at the national level in Switzerland between 2011 and 2019, the Cochrane-Armitage trend test was used. This test is suitable to identify a linear trend in a binary variable, where the null hypothesis is that there is no trend,[27] i.e., that fall rates have remained the same over time. For purposes of visualisation, a linear trend line was plotted based on a linear regression.

Third, to answer the second research question as to whether the trend persists when adjusting for changes in patient-related fall risk factors, we first set up the inpatient fall risk adjustment model by following the procedure according to Bernet, et al. [13]. As a starting point, we performed a logistic regression that included all available patient-related variables (see table 1) as covariates and, to depict a trend, the time variable as a numerical covariate (coded from 1 to 9, where 1 denotes survey year 2011 and 9 survey year 2019). Furthermore, to test for a curvilinear trend, the numerical time variable was squared (quadratic term) and also included in the model. If a statistically significant quadratic term is identified in the model, this indicates, for example, a non-linear decrease in national fall rates over time. That is, there is a negative trend over time, namely, one which is not linear but flattens out



For peer review only - http://bmjopen.bmj.com/site/about/guidelines.xhtml

over time. We then applied a stepwise backward variable selection algorithm based on the Akaike Information Criterion (AIC)[28] to determine the relevant patient-related fall risk factors to be included in the risk adjustment model and the presence of a linear and or curvilinear trend. Since the data have a hierarchical structure (patients grouped in hospitals), the selected variables were then modelled as fixed effects and the hospitals as a random effect in a multilevel logistic regression analysis. The analysis was performed twice. In the first model, both the selected patient-related and time-related factors were considered as covariates. As the model controls for changes in patient-related fall risk factors, it was used to determine the risk-adjusted trend in the national inpatient fall rates. Therefore, changes in patient-related fall risk factors can largely be excluded as a reason for significant increasing/decreasing trends observed in predicted national fall rates based on this model. Such an increase/decrease is, rather, more likely to be attributable to a deterioration/improvement in the quality of care provided in hospitals over time. In the second model, only the selected patient-related fall risk factors were considered as covariates, thus testing the hypothesis that patient-related fall risk factors have increased over time. If, based on this model, the predicted national inpatient fall rate per survey year increases, the hypothesis can be confirmed: it can be assumed that a risk adjustment is necessary. Finally, the predicted risk-adjusted trend in national inpatient fall rates based on the first model and the risk-adjusted national inpatient fall rates based on the second model were graphically plotted.

Fourth, as part of a sensitivity analysis, we repeated the complete data analysis only with hospitals that provided data in all nine survey years to assess for potential bias in the results from hospitals that had joined or left the measurement during the study period.



For peer review only - http://bmjopen.bmj.com/site/about/guidelines.xhtml

Data cleaning and merging of the data sets was carried out with IBM© SPSS© Statistics (version 28). All analyses and graphics were carried out with the statistics programme R, version 4.1.0[29] and the packages “DescTools”[30] “MASS”[31], “lme4”[32], “effects”[33 34] and “sjplot”[35]. In the analyses, a p-value < .05 was set as the statistical significance level.

# Results

*Sample description*

A total of 154506 inpatients were recorded in 224 Swiss acute care hospitals on the survey days over the 9 survey years. Of these, 115619 patients actually participated in the surveys. This corresponds to an average participation rate of 74.8%. The participation rate was lowest in 2011 with 67.7% and highest in 2018 with 76.6%. The main reasons for non-participation were refusal to participate, unavailability of patients at the time of survey or cognitive impairment. Due to missing information on care dependency in 2011, 4726 patients had to be excluded from the analyses, so that the results presented below are based on a total of 110893 cases from 222 hospitals. The patient characteristics of the sample are described in table 2.

*Table 2: Description of the patient characteristics of the sample*

| **Patient characteristics** | *n* = 110893 | % |
|---|---|---|
| Sex [female] | 56015 | 50.5 |
| Care dependency (CDS) | | |
| care independent [70-75] | 59875 | 54.0 |
| to a great extent independent [60-69] | 25970 | 23.4 |
| partially dependent [45-59] | 15508 | 14.0 |
| to a great extent dependent [25-44] | 6992 | 6.3 |
| completely dependent [15-24] | 2548 | 2.3 |
| Surgical procedure within 14 days prior to measurement [yes] | 47233 | 42.6 |
| ICD-10 – Diseases of the circulatory system [yes] | 58987 | 53.2 |



For peer review only - http://bmjopen.bmj.com/site/about/guidelines.xhtml

| | | |
|---|---|---|
| ICD-10 – Diseases of the musculoskeletal system and connective tissue [yes] | 42349 | 38.2 |
| ICD-10 – Endocrine, nutritional and metabolic diseases [yes] | 34244 | 30.9 |
| ICD-10 – Diseases of the genitourinary system [yes] | 32079 | 28.9 |
| ICD-10 – Diseases of the digestive system [yes] | 27884 | 25.1 |
| ICD-10 – Diseases of the respiratory system [yes] | 25600 | 23.1 |
| ICD-10 – Neoplasms [yes] | 21167 | 19.1 |
| ICD-10 – Mental, behavioural and neurodevelopmental disorders [yes] | 19827 | 17.9 |
| ICD-10 – Diseases of the blood and blood-forming organs [yes] | 15461 | 13.9 |
| ICD-10 – Diseases of the nervous system [yes] | 13494 | 12.2 |
| ICD-10 – Certain infectious and parasitic diseases [yes] | 13494 | 12.2 |
| ICD-10 – Diseases of the skin and subcutaneous tissue [yes] | 7572 | 6.8 |
| ICD-10 – Injury, poisoning and certain other consequences of external causes [yes] | 6637 | 6.0 |
| ICD-10 – Diseases of the eye and adnexa OR ICD-10 – Diseases of the ear and mastoid process [yes] | 8148 | 7.3 |
| ICD-10 – Congenital malformations, deformations and chromosomal abnormalities [yes] | 543 | 0.5 |
| | Median | IQR |
| Age [in years] | 70 | 24 |

*Unadjusted trend in national inpatient fall rates*

The national inpatient fall rate over the 9 years of survey was 3.7%. As figure 1 shows, the national inpatient fall rate was highest in 2011 at 4.6% and lowest in 2015 at 3.0%. At the last survey in 2019, the fall rate was 3.7%. Using the Cochran-Armitage trend test, a significant linear decreasing trend over time was found (*p* for trend = 0.004). This means that the national fall rate has decreased linearly over time when patient-related fall risk factors are not adjusted for. The estimated linear trend line (see also figure 1) intersects the intercept y at a predicted value of 4.15% in 2011 and reaches a predicted value of 3.41% in 2019.

The national inpatient fall rate in 2015 deviates from the rates of the other survey years. In a sensitivity analysis we excluded the data of the survey year 2015 and checked whether the results of the Cochran-Armitage trend test become significantly

For peer review only



For peer review only - http://bmjopen.bmj.com/site/about/guidelines.xhtml

different or not. Since a significant linear decreasing trend could be found both with and without the data from 2015, these were retained in the following analyses.

*Risk-adjusted trend of Swiss national inpatient fall rate*

According to table 3, a statistically significant decreasing curvilinear trend, indicated by the negative coefficient (coeff.) of the time variable (-0.10, *SE* 0.03, *p*=0.002) and the positive coefficient of the squared time variable (0.01, *SE* 0.00, *p*=0.049) in the model, was observed in inpatient fall rates at the national level when patient-related fall risk factors were controlled for. The curvilinear trend indicates that the relationship between time and inpatient falls at national level is not linear but follows a U-shaped curve, meaning that the decreasing trend found flattens out over time. The calculated odds ratios (online supplemental table S1) illustrate the observed decreasing curvilinear trend in that, for example, at the national level, the odds of falling in hospital decreased by 8% in 2012 compared to 2011 (*OR* 0.92, 95% *CI* 0.88-0.96). In contrast, however, the odds did not decrease in 2019 compared to 2018 (*OR* 1.00, 95% *CI* 0.96-1.04).

The above-described trend was observed while controlling for a total of twelve patient-related fall risk variables in the risk adjustment model (table 3): eight significantly fall risk-increasing patient-related variables, such as age (*OR* 1.02, 95% *CI* 1.01–1.02, $p < 0.001$), being to a great extent care dependent compared to care independent (*OR* 5.67, 95% *CI* 5.08–6.34, $p < 0.001$) or in the ICD-10 diagnosis group Mental and Behavioural disorders (*OR* 1.79, 95% *CI* 1.67–1.92, $p < 0.001$); two significantly fall risk-reducing patient-related characteristics, sex (*OR* 0.82, 95% *CI* 0.77–0.87, $p < 0.001$) and surgery in the last 14 days prior to measurement (*OR* 0.61, 95% *CI* 0.57–0.66, $p < 0.001$); two non-significant ICD-10 diagnosis groups,



For peer review only - http://bmjopen.bmj.com/site/about/guidelines.xhtml

Certain infectious and parasitic diseases (*OR* 1.09, 95% *CI* 1.00–1.18, *p* = 0.064) and Diseases of the genitourinary system (*OR* 0.95, 95% *CI* 0.89–1.02, *p* = 0.190).

*Table 3: Overview of the multilevel logistic regression model used to derive the risk-adjusted trend in Swiss national inpatient fall rates*

| | Risk-adjusted trend in national inpatient fall rates | | | | |
|---|---|---|---|---|---|
| *Predictors* | *coeff.* | *SE* | *p-value* | *OR* | *95% CI* |
| (Intercept) | -5.03 | 0.12 | **<0.001** | - | - |
| **Time related factors (trend)** | | | | | |
| Time | -0.10 | 0.03 | **0.002** | - | - |
| (Time)^2 | 0.01 | 0.00 | **0.049** | - | - |
| **Patient related fall risk factors** | | | | | |
| Age [in years] | 0.02 | 0.00 | **<0.001** | 1.02 | 1.01 – 1.02 |
| CDS [care independent (70-75)] | Ref. | | | | |
| CDS [to a great extent independent (60-69)] | 1.04 | 0.05 | **<0.001** | 2.83 | 2.57 – 3.11 |
| CDS [partially dependent (45-59)] | 1.42 | 0.05 | **<0.001** | 4.14 | 3.75 – 4.58 |
| CDS [to a great extent dependent (25-44)] | 1.74 | 0.06 | **<0.001** | 5.67 | 5.08 – 6.34 |
| CDS [completely dependent (15-24)] | 1.31 | 0.09 | **<0.001** | 3.70 | 3.12 – 4.38 |
| ICD-10 – Mental and Behavioural disorders [yes] | 0.58 | 0.04 | **<0.001** | 1.79 | 1.67 – 1.92 |
| ICD-10 – Neoplasms [yes] | 0.37 | 0.04 | **<0.001** | 1.45 | 1.35 – 1.56 |
| ICD-10 – Diseases of the nervous system [yes] | 0.28 | 0.04 | **<0.001** | 1.32 | 1.22 – 1.44 |
| ICD-10 – Diseases of the blood and blood-forming organs [yes] | 0.21 | 0.04 | **<0.001** | 1.23 | 1.13 – 1.33 |
| ICD-10 – Endocrine, nutritional and metabolic diseases [yes] | 0.13 | 0.03 | **<0.001** | 1.13 | 1.06 – 1.21 |
| ICD-10 – Injury, poisoning, other consequences of external causes [yes] | 0.14 | 0.06 | **0.023** | 1.15 | 1.02 – 1.29 |
| ICD-10 – Certain infectious and parasitic diseases [yes] | 0.08 | 0.04 | 0.064 | 1.09 | 1.00 – 1.18 |
| ICD-10 – Diseases of the genitourinary system [yes] | -0.05 | 0.04 | 0.190 | 0.95 | 0.89 – 1.02 |
| Sex [female] | -0.20 | 0.03 | **<0.001** | 0.82 | 0.77 – 0.87 |
| Surgical procedure within 14 days prior to measurement [yes] | -0.49 | 0.04 | **<0.001** | 0.61 | 0.57 – 0.66 |
| **Random Effects** | | | | | |
| $\tau_{00}$ [variability in hospital intercepts] | | | 0.08 | | |
| N [hospitals] | | | 222 | | |
| Observations | | | 110893 | | |

Significant *p*-values are highlighted in bold.

Figure 2 illustrates the predicted national fall rates per survey year when the following are adjusted for: a) only patient-related fall risk factors (red line), or, b) patient-related fall risk factors as well as a curvilinear time trend (green line). The red line shows that patient-related fall risk factors have tended to increase over time, which is reflected in increasing predicted risk-adjusted national fall rates. These are estimated to be 3.66% in year 2011 and 4.04% in year 2019. In contrast, the green



For peer review only

For peer review only - http://bmjopen.bmj.com/site/about/guidelines.xhtml

line shows a decrease followed by a slight increase in predicted national inpatient fall rates over time when adjusting for both changes in patient-related fall risk factors as well as a curvilinear time trend. The predicted fall rates based on this model are highest at 4.45% in 2011, lowest at 3.44% in 2016 and are 3.72% in 2019.

*Sensitivity analysis*

The sensitivity analysis included 122 hospitals that provided data over all nine survey years, with a total of 90661 patients. The results based on this subsample do not differ significantly from the overall sample: First, a significant decreasing linear trend (*p* for Cochran-Armitage trend test = 0.011) was also observed (online supplemental figure S1). Second, the same patient-related fall risk variables were selected into the risk adjustment model and the coefficients of the risk variables in the model were not substantially different (online supplemental table S2). Third, a significant decreasing curvilinear trend, indicated by the negative coefficient of the time variable (-0.12, *SE* 0.04, *p*=0.001) and the positive coefficient of the squared time variable (0.01, *SE* 0.00, *p*=0.024) in the model was also found after risk adjustment (online supplemental table S2, table S3 and figure S2).

# Discussion

In this study, we investigated whether inpatient fall rates in acute care hospitals in Switzerland have changed at the national level between 2011 and 2019, and whether these changes persist when patient-related fall risk factors are adjusted for. In both the unadjusted analysis and the risk-adjusted analysis controlling for changing patient-related fall risk factors over time, a significant decreasing trend in inpatient fall rates at the national level was found across the survey years. Similar results were found some years ago based on National Database of Nursing Quality



For peer review only - http://bmjopen.bmj.com/site/about/guidelines.xhtml

Indicators (NDNQI) data in US hospitals over a 27-month as well as six-year time period by observing a non-adjusted decreasing trend in fall rates.[36 37] More recently, a study found that combined inpatient falls and pressure ulcer rates in the US decreased statistically significantly from 2010 to 2019 after risk adjustment.[4]

It is assumed that the negative trend in risk-adjusted national fall rates observed in our study between 2011 and 2019 is largely due to continuous quality improvement of structures and processes for fall prevention in hospitals. This assumption is reinforced by a study which showed that improvements designed to prevent falls were implemented at both the structural and process level in Swiss hospitals during the same time period.[14] For example, the average relative frequency of the number of fall prevention measures applied per patient, such as the evaluation of current medication or the evaluation of aid devices, increased significantly from 12.9% in 2011 to 23.1% in 2019.[14] The impetus for the improvement measures put in place by hospitals is likely to have been facilitated by the well-known quality improvement mechanisms associated with the introduction of national inpatient falls measurement in 2011. Although regular quality measurement alone does not automatically lead to quality improvement,[1] accompanying measures such as public reporting,[38] comparative benchmarking[39] and performance feedback via quality dashboard[40 41] are described as effective in triggering quality improvement activities in hospitals.

Nevertheless, despite the quality improvement measures implemented by hospitals to prevent falls, the risk-adjusted national fall rates did not decline linearly over time; rather, the decline was greater at the beginning of the surveys and then gradually levelled off, as demonstrated by the significant curvilinear effect observed in our study. About the reasons for this curvilinear effect, we can only speculate. Generally, it can be assumed that the lower fall rates get, the more difficult it



For peer review only - http://bmjopen.bmj.com/site/about/guidelines.xhtml

becomes to reduce them further, as not all fall events are preventable.[8] Another explanation could be that, given the increase in patient-related fall risk factors over time, more and more effort is required on the part of hospitals just to keep fall rates constant and, accordingly, it becomes increasingly challenging to reduce them further.[14] This possible link is particularly emphasised by the results of the present study, which showed an increase in patient-related fall risk factors and an increase in predicted fall rates at national level over time when prediction is based on these patient-related fall risk factors.

Almost more important than identifying the reasons for the curvilinear trend is the fact that it indicates a potential increase in fall rates at national level in the coming years if the observed trend continues. This development could be exacerbated by a further increase in fall risk factors and patient complexity in hospital, as well as by a shortage of nursing staff, especially registered nurses, which is expected to worsen in the coming years both internationally[15] and in Switzerland[42]. A shortage of registered nurses has already been linked in a meta-analysis to poorer overall patient outcomes[43] and, in an observational study, to higher fall rates in hospital[44]. Under these circumstances, it is strongly recommended that inpatient fall rates should be continuously monitored. On the one hand, to inform decision-makers at all levels and health professionals about potential deteriorations in the quality of care provided and to enable initiation of appropriate data-driven countermeasures, and on the other hand, to investigate whether the curvilinear effect found is confirmed in the future or whether it represents a sample-specific observation.

It should be noted that due to the large sample in our study even small differences become statistically significant. Therefore, as in the study by Bouldin, et al. [36], the question arises whether the observed decline in fall rates over time is also value-



For peer review only - http://bmjopen.bmj.com/site/about/guidelines.xhtml

based. In our study, we found an absolute reduction in predicted risk-adjusted fall rates of 0.73% between 2011 and 2019. Extrapolated to the slightly more than 1.2 million cases in general hospitals in 2019 in Switzerland[45], this means that the quality improvement measures implemented over the years prevented 8760 fall events in 2019 compared to 2011. This decrease may seem small, but considering the possible impact of a fall on the individual, such as injury or death[46], this decrease is nevertheless remarkable and of great value to patients. The decrease is also valuable from an economic point of view. Multiplying the prevented fall events by the average cost of a fall according to Morello, et al. [7] of $6669, it is estimated that almost $60 million in healthcare costs could be saved nationwide in 2019.

*Strength and Limitations*

A strength of our study is that the data used was collected using a highly standardised, internationally proven method and based on a census survey of all Swiss hospitals; it thus accurately reflects the situation at the national level. However, due to the average participation rate of patients of just under 75%, a possible selection bias cannot be ruled out. Likewise, a recall bias cannot be ruled out either, since the patients were asked whether they had fallen in hospital in the last 30 days. As with the selection bias, the recall bias could have led to a potential underestimation of the fall rates observed. Since it can be assumed that the underestimation, if present, has remained constant over time, i.e. a systematic underestimation, this would have no influence on the trend found. Due to questionnaire adjustments over time, not all relevant fall risk variables, such as a fall in the last 12 months or the use of sedatives/psychotropic drugs (only collected from 2016 onwards), could be considered in the risk adjustment model. Although these variables were not available for the present analyses, on the whole, the same



For peer review only - http://bmjopen.bmj.com/site/about/guidelines.xhtml

patient-related characteristics with similar coefficients were identified as fall risk variables as in our previous studies.[13 47] Due to the cross-sectional survey design, a temporal link between the fall risk factors and the outcome is not possible. In this context, it is recommended that future trend analyses use data from all patients covering the period from admission to discharge as well as relevant risk adjustment variables, e.g. based on routine clinical data as suggested by Bernet, et al. [48].

## Conclusions

In our study, we found a significant decreasing trend in fall rates in Swiss hospitals, both descriptively and after adjustment for patient-related fall risk factors. It can be assumed that the introduction of the annual national measurement of inpatient falls in 2011 triggered improvement measures in hospitals in Switzerland, which in turn manifested in lower fall rates over time. The quality improvement achieved has had positive effects both for patients and for society as a whole by saving costs. However, the identified curvilinear effect also showed that the fall rates decreased more at the beginning of the measurements than later on. This could be an indication that fall rates may increase in the future, especially in light of future challenges such as increasing fall risk factors, as shown in our study, and the staff shortages that hospitals are facing. The extent to which the observed curvilinear trend will be confirmed in the future requires further investigation. In this context, it is recommended that national policy makers continue to collect and monitor the fall rates in hospitals, on the one hand because this enables data-based quality development in the hospitals, and on the other so that a possible increase in national fall rates can be recognised at an early stage and appropriate targeted quality-improvement measures can be planned and implemented at national level.



For peer review only - http://bmjopen.bmj.com/site/about/guidelines.xhtml

For peer review only

For this, robust, reliable and fair comparisons over time are important. Risk adjustment can be recommended as a method for improving the comparability of results over time. To address the limitations of the present study, it is also recommended to further investigate to what extent routine clinical data are suitable as a basis for national comparisons of fall rates over time including risk adjustment.

## Acknowledgements

We would like to thank all hospitals and patients who participated in the surveys.

## Competing interests

None declared.

## Funding

The annual national measurement of inpatient falls in Switzerland and thus the data collection was financed by the Swiss National Association for Quality Development in Hospitals and Clinics (ANQ) and carried out under mandate by the Bern University of Applied Sciences (BFH). The present data analysis was also financed by ANQ.

## Data availability statement

The data on which the present results are based can be obtained on reasonable request from the Swiss National Association for Quality Development in Hospitals and Clinics (ANQ) (see www.anq.ch). However, the availability of these data may be subject to restrictions in accordance with the "Empfehlungen Verwendung von ANQ-



For peer review only - http://bmjopen.bmj.com/site/about/guidelines.xhtml

For peer review only

Daten zu Forschungszwecken [Recommendations for the use of ANQ data for research purposes]", which can be found at www.anq.ch.

# Ethics statements

### Patient consent for publication

Not applicable.

### Ethics approval

This study was conducted in accordance with the ethical principles of the Declaration of Helsinki. Full Research Ethics Committee approval was granted by the Ethics Committee of the Canton of Bern on 4 October 2011 (application no. 122/11) and the other twelve local ethics committees. As of 2012, Swiss ethics and the cantonal ethics committees declared the measurement as a quality measurement, which does not require ethical approval because it is not subjected to the Swiss Human Research Act. Furthermore, in 2022, the Ethics Advisory Board of the Bern University of Applied Sciences declared that the anonymised data from the LPZ measurement could be used again without further approval from a cantonal ethics committee (Ref. EAB2022_012).

All patients or their legal representatives gave their verbal informed consent to participate in the measurement, and only fully anonymised data were used in the present study.

# Contributors

Conceptualization of the measurement, NB, IE, SH, JS; conceptualization of the manuscript, NB, MM; data collection and curation, NB, MM; data analysis, NB, MM;



For peer review only - http://bmjopen.bmj.com/site/about/guidelines.xhtml

data interpretation, NB, IE, SH, MM, JS; writing – original draft preparation, NB; writing – review and editing, IE, SH, MM, JS.

NB is responsible for the overall content of the manuscript as guarantor.

## Patient and public involvement

Patients and/or the public were not involved in the design, or conduct, or reporting or dissemination plans of this research.

## References

1. Agweyu A, Hill K, Diaz T, et al. Regular measurement is essential but insufficient to improve quality of healthcare. *BMJ* 2023;380:e073412. doi: https://doi.org/10.1136/bmj-2022-073412
2. Busse R, Klazinga N, Panteli D, et al. Improving healthcare quality in Europe: Characteristics, effectiveness and implementation of different strategies. Health Policy Series: OECD & Observatory, 2019.
3. Berwick DM, James B, Coye MJ. Connections Between Quality Measurement and Improvement. *Medical Care* 2003;41(1):I-30-I-38. doi: https://doi.org/10.1097/00005650-200301001-00004
4. Eldridge N, Wang Y, Metersky M, et al. Trends in Adverse Event Rates in Hospitalized Patients, 2010-2019. *Jama* 2022;328(2):173-83. doi: https://doi.org/10.1001/jama.2022.9600
5. LeLaurin JH, Shorr RI. Preventing Falls in Hospitalized Patients: State of the Science. *Clinics in geriatric medicine* 2019;35(2):273-83. doi: https://doi.org/10.1016/j.cger.2019.01.007
6. Slawomirski L, Klazinga N. The economics of patient safety: From analysis to action: OECD, 2020.
7. Morello RT, Barker AL, Watts JJ, et al. The extra resource burden of in-hospital falls: a cost of falls study. *Medical Journal of Australia* 2015;203(9):367. doi: https://doi.org/10.5694/mja15.00296
8. Registered Nurses' Association of Ontario. Preventing Falls and Reducing Injury from Falls. 4th ed. Toronto, ON., 2017.
9. NDNQI - Nursing excellence made simple: PressGaney; 2023 [Available from: https://www.pressganey.com/platform/ndnqi/ accessed 11 July 2023.
10. Staggs VS, Davidson J, Dunton N, et al. Challenges in defining and categorizing falls on diverse unit types: lessons from expansion of the NDNQI Falls Indicator. *Journal of Nursing Care Quality* 2015;30(2):106-12. doi: https://doi.org/10.1097/ncq.0000000000000085
11. Halfens RJG, Meesterberends E, van Nie NC, et al. International prevalence measurement of care problems: results. *Journal of Advanced Nursing* 2013;69(9):e5-c17. doi: https://doi.org/10.1111/jan.12189



For peer review only - http://bmjopen.bmj.com/site/about/guidelines.xhtml

12. Van Nie NC, Schols JMGA, Meesterberends E, et al. An international prevalence measurement of care problems: study protocol. *Journal of Advanced Nursing* 2013;69(9):c18-c29. doi: https://doi.org/10.1111/jan.12190
13. Bernet NS, Everink IHJ, Schols JMGA, et al. Hospital performance comparison of inpatient fall rates; the impact of risk adjusting for patient-related factors: a multicentre cross-sectional survey. *BMC Health Services Research* 2022;22(1):225. doi: https://doi.org/10.1186/s12913-022-07638-7
14. Thomann S, Zimmermann R, Riedweg JS, et al. National developments in falls and pressure ulcers in Swiss hospitals from 2011-2019: a secondary data analysis of the national quality measurement. *Manuscript submitted for publication* 2023,
15. World Health Organization (WHO). Regional Office for Europe. Health and care workforce in Europe: time to act. Copenhagen: World Health Organization. Regional Office for Europe, 2022.
16. Lane-Fall MB, Neuman MD. Outcomes measures and risk adjustment. *International Anesthesiology Clinics* 2013;51(4):10-21. doi: https://doi.org/10.1097/AIA.0b013e3182a70a52
17. Everink I, Gordon A, Rijcken S, et al. Learning and improving in long-term care internationally: the International Prevalence Measurement of Care Quality (LPZ). *Geriatrics, Gerontology and Aging* 2021;15(2021) doi: http://dx.doi.org/10.53886/gga.e0210056
18. Bours GJ, Halfens RJG, Lubbers M, et al. The development of a national registration form to measure the prevalence of pressure ulcers in the Netherlands. *Ostomy Wound Management* 1999;45(11):28-33, 36-8, 40.
19. Gorecki C, Brown J, Cano S, et al. Development and validation of a new patient-reported outcome measure for patients with pressure ulcers: the PU-QOL instrument. *Health and Quality of Life Outcomes* 2013;11(1):95. doi: https://doi.org/10.1186/1477-7525-11-95
20. Halfens RJG, Meesterberends E, Meijers JMM, et al. Landelijke Prevalentiemeting Zorgproblemen. Rapportage resultaten 2011. Maastricht: Universiteit Maastricht, CAPHRI school for Public Health and Primary care, Department of Health Services Research, 2011:1-49.
21. Kellogg International Work Group on the Prevention of Falls by the Elderly. The prevention of falls in later life. A report of the Kellogg International Work Group on the Prevention of Falls by the Elderly. *Danish medical bulletin* 1987;34(Supplement 4):1-24.
22. World Health Organization. International Statistical Classification of Diseases and Related Health Problems 10th Revision 2016 [Available from: https://icd.who.int/browse10/2016/en accessed January 19, 2023.
23. Dijkstra A, Smith J, White M. Manual Care Dependency Scale. Measuring care dependency with the Care Dependency Scale (CDS). A manual, 2006.
24. Lohrmann C, Dijkstra A, Dassen T. The Care Dependency Scale: an assessment instrument for elderly patients in German hospitals. *Geriatric Nursing* 2003 doi: https://doi.org/10.1067/mgn.2003.8
25. Eglseer D, Halfens RJG, Schols JMGA, et al. Dysphagia in Hospitalized Older Patients: Associated Factors and Nutritional Interventions. *Journal of Nutrition, Health and Aging* 2018;22(1):103-10. doi: https://doi.org/10.1007/s12603-017-0928-x
26. Schüttengruber G, Halfens RJG, Lohrmann C. Care dependency of patients and residents at the end of life: A secondary data analysis of data from a cross-sectional study in hospitals and geriatric institutions. *Journal of Clinical Nursing* 2022;31(5-6):657-68. doi: https://doi.org/10.1111/jocn.15925



For peer review only - http://bmjopen.bmj.com/site/about/guidelines.xhtml

27. Armitage P. Tests for Linear Trends in Proportions and Frequencies. *Biometrics* 1955;11(3):375-86. doi: https://www.doi.org/10.2307/3001775
28. Akaike H. A new look at the statistical model identification. *IEEE Transactions on Automatic Control* 1974;19(6):716-23. doi: https://doi.org/10.1109/TAC.1974.1100705
29. R: A Language and Environment for Statistical Computing [program]. Vienna, Austria: R Foundation for Statistical Computing, 2021.
30. DescTools: Tools for Descriptive Statistics [program]. R package version 0.99.48 version, 2023.
31. Venables WN, Ripley BD. Modern Applied Statistics with S. Fourth ed. New York: Springer 2002.
32. Bates D, Mächler M, Bolker B, et al. Fitting Linear Mixed-Effects Models Using lme4. *Journal of Statistical Software* 2015;67(1):1-48. doi: https://doi.org/10.18637/jss.v067.i01
33. Fox J. Effect Displays in {R} for Generalised Linear Models. *Journal of Statistical Software* 2003;8(15):1–27. doi: https://doi.org/10.18637/jss.v008.i15
34. Fox J, Weisberg S. An R Companion to Applied Regression. 3rd ed. Thousand Oaks CA: Sage 2019.
35. sjPlot: Data Visualization for Statistics in Social Science [program], 2021.
36. Bouldin ELD, Andresen EM, Dunton NE, et al. Falls among adult patients hospitalized in the United States: prevalence and trends. *Journal of Patient Safety* 2013;9(1):13-17. doi: https://doi.org/10.1097/PTS.0b013e3182699b64
37. He J, Dunton N, Staggs V. Unit-level Time Trends in Inpatient Fall Rates of US Hospitals. *Medical Care* 2012;50(9):801-07. doi: https://doi.org/10.1097/mlr.0b013e31825a8b88
38. Campanella P, Vukovic V, Parente P, et al. The impact of Public Reporting on clinical outcomes: a systematic review and meta-analysis. *BMC Health Services Research* 2016;16(1):296. doi: https://doi.org/10.1186/s12913-016-1543-y
39. Willmington C, Belardi P, Murante AM, et al. The contribution of benchmarking to quality improvement in healthcare. A systematic literature review. *BMC Health Services Research* 2022;22(1):139. doi: https://doi.org/10.1186/s12913-022-07467-8
40. Brown B, Gude WT, Blakeman T, et al. Clinical Performance Feedback Intervention Theory (CP-FIT): a new theory for designing, implementing, and evaluating feedback in health care based on a systematic review and meta-synthesis of qualitative research. *Implementation Science* 2019;14(1):40. doi: https://doi.org/10.1186/s13012-019-0883-5
41. Dowding D, Randell R, Gardner P, et al. Dashboards for improving patient care: Review of the literature. *International Journal of Medical Informatics* 2015;84(2):87-100. doi: https://doi.org/10.1016/j.ijmedinf.2014.10.001
42. Merçay C, Grünig A, Dolder P. Gesundheitspersonal in der Schweiz – Nationaler Versorgungsbericht 2021. Bestand, Bedarf, Angebot und Massnahmen zur Personalsicherung. Obsan Bericht. Neuchâtel: Schweizerisches Gesundheitsobservatorium, 2021.
43. Dall'Ora C, Saville C, Rubbo B, et al. Nurse staffing levels and patient outcomes: A systematic review of longitudinal studies. *International Journal of Nursing Studies* 2022;134:104311. doi: https://doi.org/10.1016/j.ijnurstu.2022.104311
44. Kim J, Lee E, Jung Y, et al. Patient-level and organizational-level factors influencing in-hospital falls. *Journal of Advanced Nursing* 2022;78(11):3641-51. doi: https://doi.org/10.1111/jan.15254



For peer review only - http://bmjopen.bmj.com/site/about/guidelines.xhtml

45. Bundesamt für Gesundheit. Evaluation der KVG-Revision im Bereich der Spitalfinanzierung - Schlussbericht des BAG an den Bundesrat. Bern: Bundesamt für Gesundheit, 2019.
46. Agency for Healthcare Research and Quality. Network of Patient Safety Databases Chartbook, 2022. Rockville, MD: Agency for Healthcare Research and Quality, 2022.
47. Bernet NS, Everink IH, Hahn S, et al. Comparing risk-adjusted fall rates internationally: Validation of a risk-adjustment model using multicentre cross-sectional data from hospitals in Switzerland and Austria. *Manuscript submitted for publication* 2023,
48. Bernet NS, Thomann S, Kurpicz-Briki M, et al. Potential of Electronic Medical Record Data for National Quality Measurement. *Studies in Health Technology and Informatics* 2022;292:51-56. doi: https://doi.org/10.3233/shti220320

For peer review only



For peer review only - http://bmjopen.bmj.com/site/about/guidelines.xhtml

*Figure 1: Visualisation of the descriptive Swiss national inpatient fall rates from 2011 to 2019 and the estimated linear trend over time.*

*Figure 2: Visualisation of predicted risk-adjusted (without and with time trend) Swiss national inpatient fall rates from 2011 to 2019*

For peer review only



For peer review only - http://bmjopen.bmj.com/site/about/guidelines.xhtml

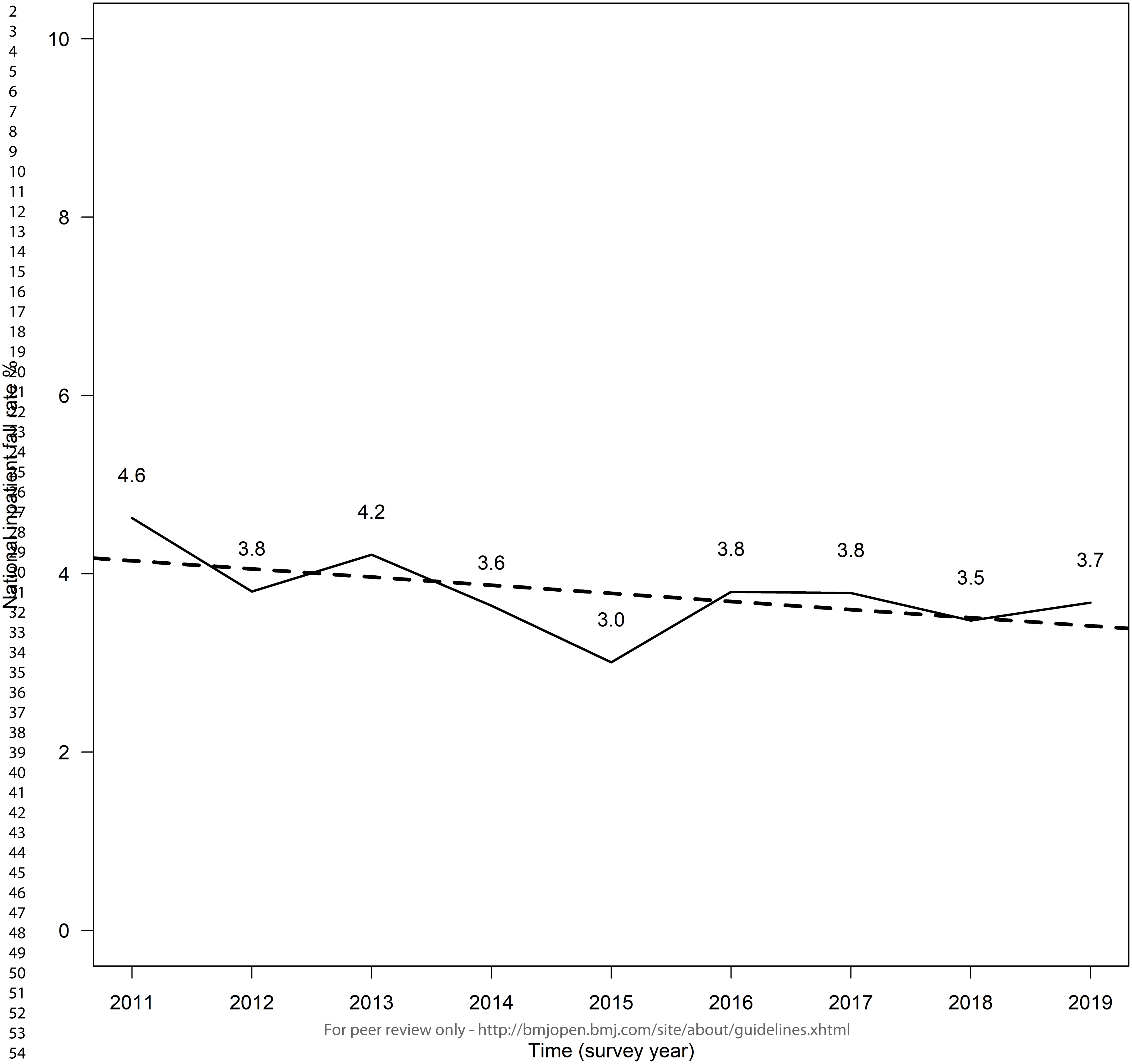
10
8
6
4
2
0
National inpatient fall rate %
4.6
3.8
4.2
3.6
3.0
3.8
3.8
3.5
3.7
2011
2012
2013
2014
2015
2016
2017
2018
2019
Time (survey year)

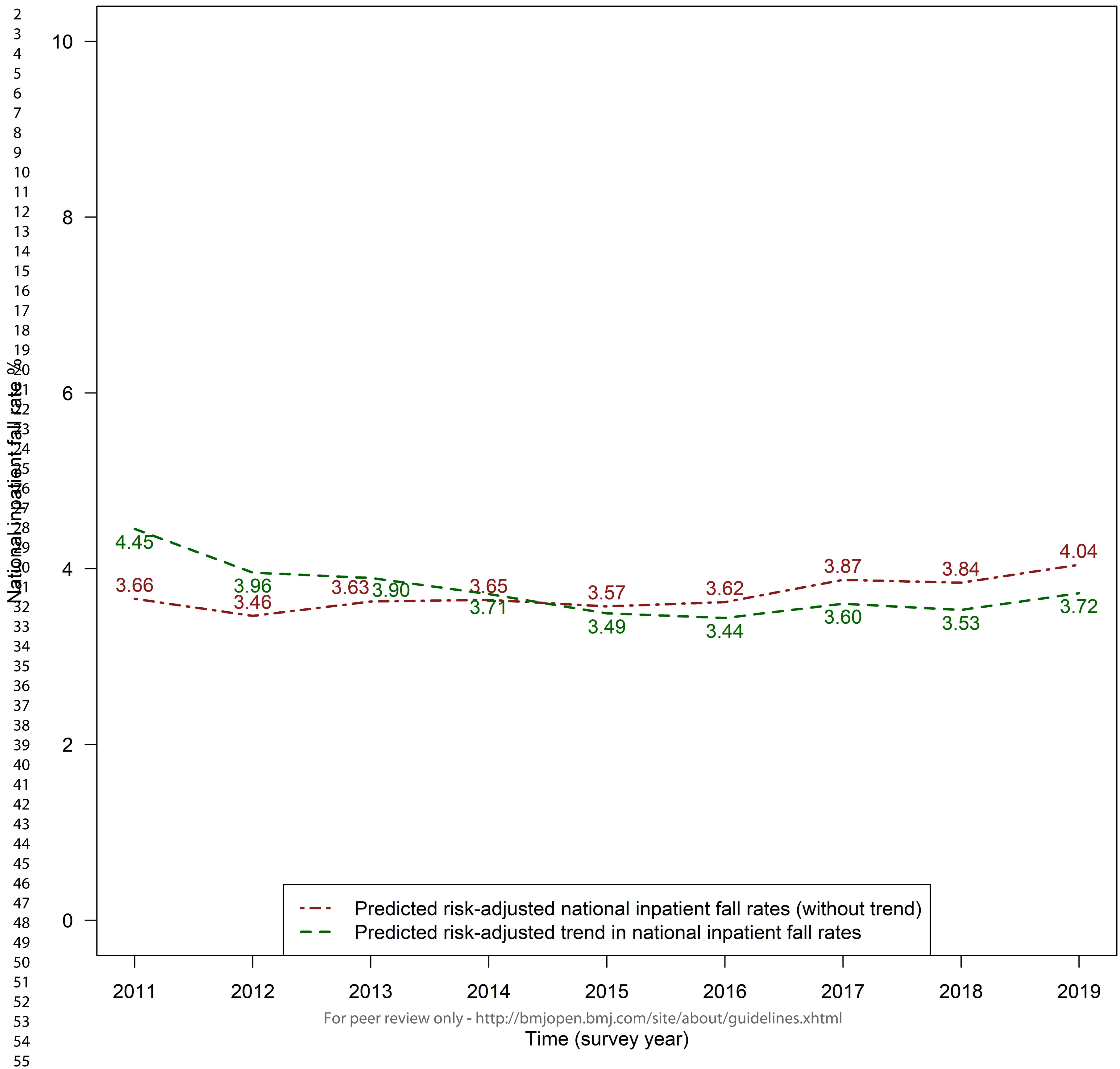


For peer review only - http://bmjopen.bmj.com/site/about/guidelines.xhtml

*Supplementary table S1: Illustration of the estimated curvilinear trend using odds ratios, each of which describes the decrease in Swiss national fall rates in relation to the previous survey year*

| **Survey year** | **2011/2012** | **2012/2013** | **2013/2014** | **2014/2015** | **2015/2016** | **2016/2017** | **2017/2018** | **2018/2019** |
|---|---|---|---|---|---|---|---|---|
| *OR* | 0.92 | 0.93 | 0.94 | 0.95 | 0.96 | 0.98 | 0.99 | 1.00 |
| 95% *CI* | 0.88 – 0.96 | 0.90 – 0.96 | 0.92 – 0.97 | 0.94 – 0.97 | 0.95 – 0.98 | 0.96 – 1.00 | 0.96 – 1.02 | 0.96 – 1.04 |

For peer review only



For peer review only - http://bmjopen.bmj.com/site/about/guidelines.xhtml

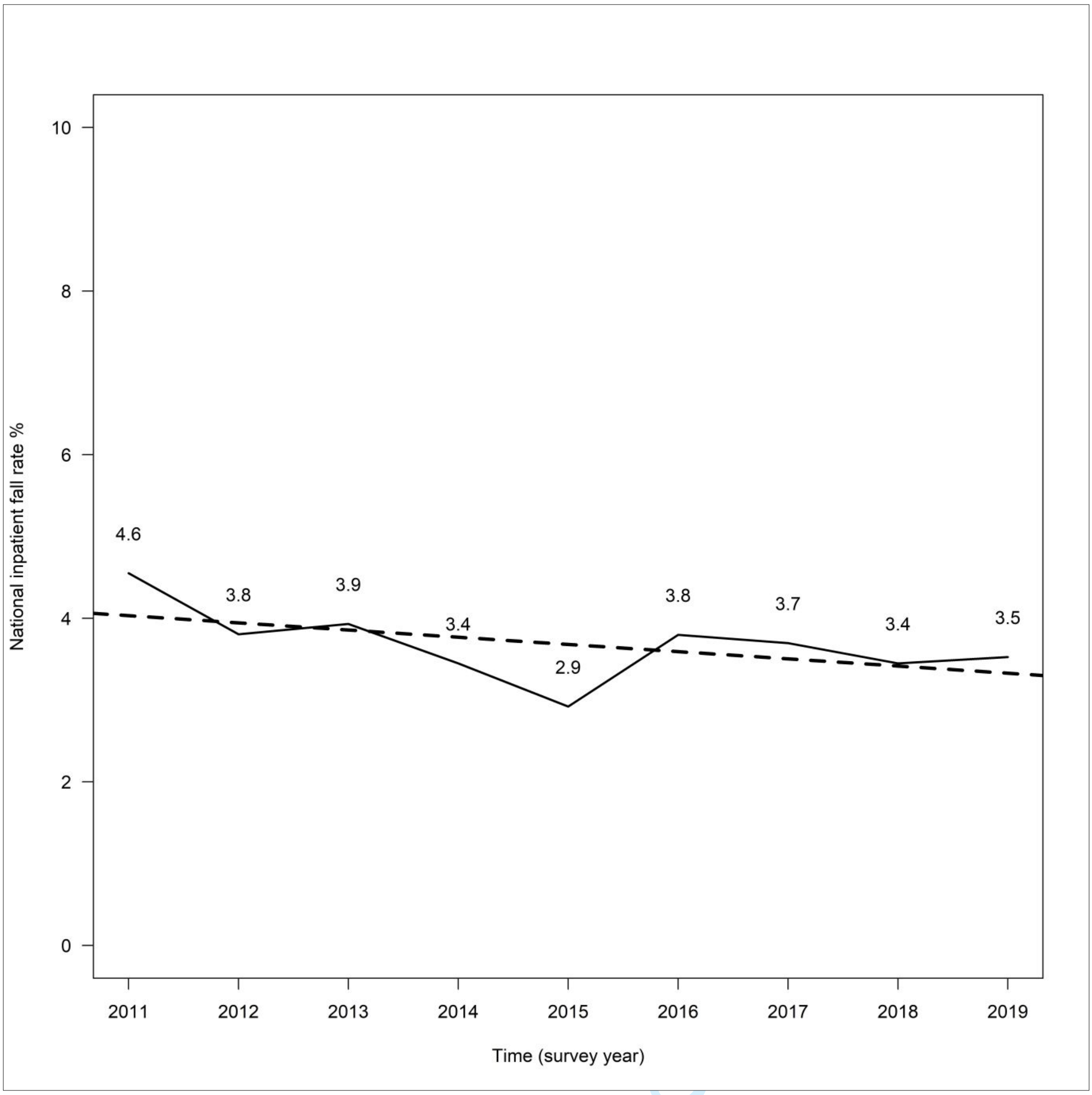


*Supplementary figure S1: Visualisation of the descriptive Swiss national inpatient fall rates from 2011 to 2019 and the estimated linear trend over time, taking into account only hospitals that provided data in all survey years*



For peer review only - http://bmjopen.bmj.com/site/about/guidelines.xhtml

*Supplementary table S2: Overview of the multilevel logistic regression model used to derive the risk-adjusted trend in Swiss national inpatient fall rates, taking into account only hospitals that provided data in all survey years*

| | **Risk-adjusted trend in national inpatient fall rates** | | | | |
|---|---|---|---|---|---|
| *Predictors* | *coeff.* | *SE* | *p-value* | *OR* | *95% CI* |
| (Intercept) | -5.06 | 0.13 | **<0.001** | - | - |
| **Time related factors (trend)** | | | | | |
| Time | -0.12 | 0.04 | **0.001** | - | - |
| (Time)^2 | 0.01 | 0.00 | **0.024** | - | - |
| **Patient related fall risk factors** | | | | | |
| Age [in years] | 0.02 | 0.00 | **<0.001** | 1.02 | 1.01 – 1.02 |
| CDS [care independent (70-75)] | | | | Ref. | |
| CDS [to a great extent independent (60-69)] | 1.03 | 0.05 | **<0.001** | 2.79 | 2.51 – 3.10 |
| CDS [partially dependent (45-59)] | 1.42 | 0.06 | **<0.001** | 4.13 | 3.70 – 4.62 |
| CDS [to a great extent dependent (25-44)] | 1.69 | 0.06 | **<0.001** | 5.41 | 4.78 – 6.13 |
| CDS [completely dependent (15-24)] | 1.26 | 0.10 | **<0.001** | 3.52 | 2.90 – 4.27 |
| ICD-10 – Mental and Behavioural disorders [yes] | 0.60 | 0.04 | **<0.001** | 1.82 | 1.68 – 1.97 |
| ICD-10 – Neoplasms [yes] | 0.37 | 0.04 | **<0.001** | 1.44 | 1.33 – 1.57 |
| ICD-10 – Diseases of the nervous system [yes] | 0.33 | 0.05 | **<0.001** | 1.38 | 1.26 – 1.52 |
| ICD-10 – Diseases of the blood and blood-forming organs [yes] | 0.25 | 0.05 | **<0.001** | 1.28 | 1.17 – 1.40 |
| ICD-10 – Endocrine, nutritional and metabolic diseases [yes] | 0.13 | 0.04 | **0.001** | 1.13 | 1.05 – 1.22 |
| ICD-10 – Injury, poisoning, other consequences of external causes [yes] | 0.13 | 0.07 | 0.055 | 1.14 | 1.01 – 1.30 |
| ICD-10 – Certain infectious and parasitic diseases [yes] | 0.09 | 0.05 | 0.059 | 1.10 | 1.00 – 1.21 |
| ICD-10 – Diseases of the genitourinary system [yes] | -0.04 | 0.04 | 0.373 | 0.97 | 0.89 – 1.04 |
| Sex [female] | -0.19 | 0.04 | **<0.001** | 0.82 | 0.77 – 0.89 |
| Surgical procedure within 14 days prior to measurement [yes] | -0.46 | 0.04 | **<0.001** | 0.63 | 0.58 – 0.69 |
| **Random Effects** | | | | | |
| $\tau_{00}$ [variability in hospital intercepts] | | | 0.07 | | |
| N [hospitals] | | | 132 | | |
| Observations | | | 90661 | | |

Significant *p*-values are highlighted in bold.

For peer review only



For peer review only - http://bmjopen.bmj.com/site/about/guidelines.xhtml

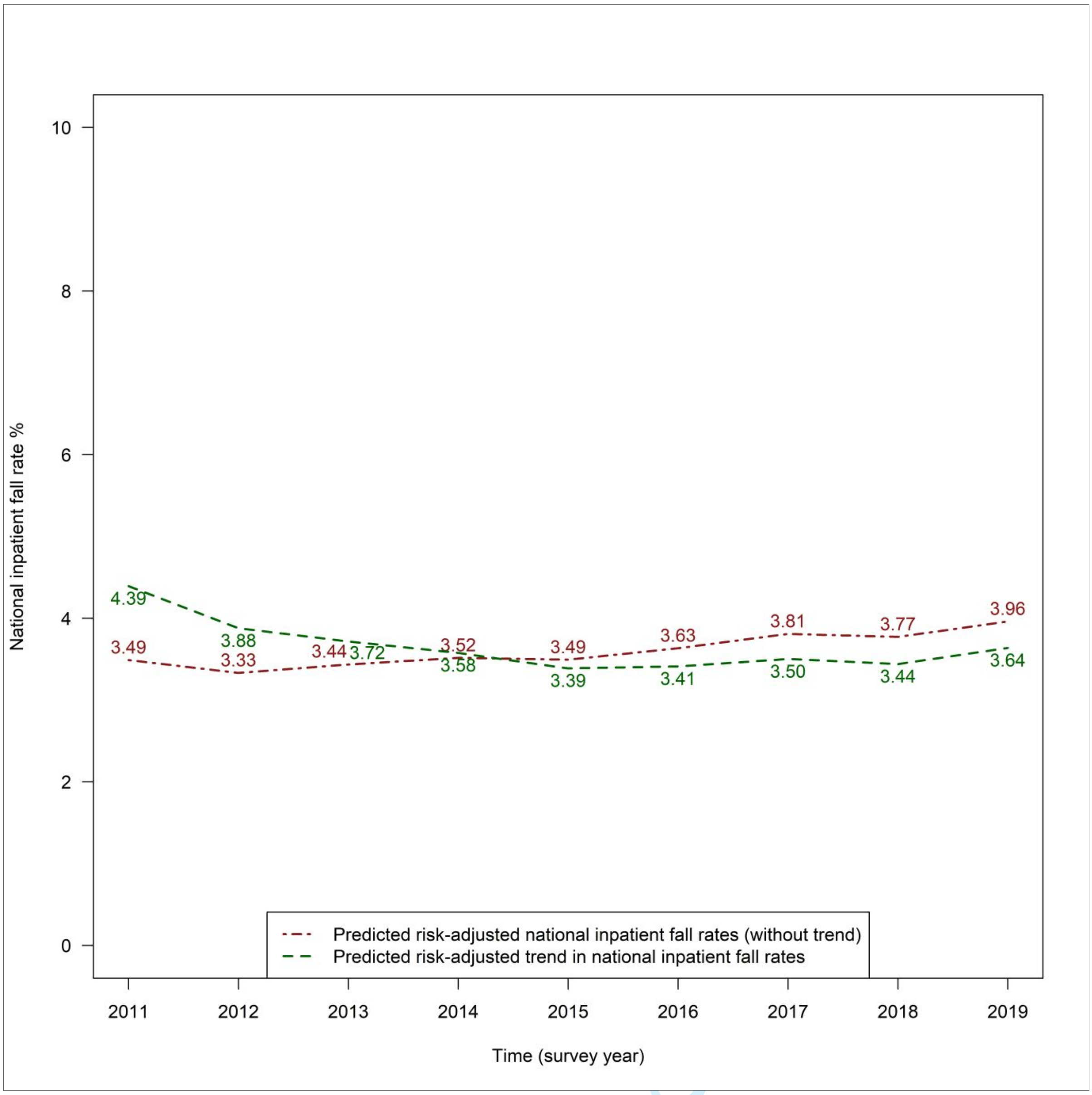


*Supplementary figure S2: Visualisation of observed and predicted risk-adjusted (without and with time trend) Swiss national inpatient fall rates from 2011 to 2019, taking into account only hospitals that provided data in all survey years*



For peer review only - http://bmjopen.bmj.com/site/about/guidelines.xhtml

*Supplementary table S3: Illustration of the estimated curvilinear trend using odds ratios, each of which describes the decrease in Swiss national fall rates in relation to the previous survey year, taking into account only hospitals that provided data in all survey years*

| Survey year | 2011/2012 | 2012/2013 | 2013/2014 | 2014/2015 | 2015/2016 | 2016/2017 | 2017/2018 | 2018/2019 |
|---|---|---|---|---|---|---|---|---|
| ***OR*** | 0.91 | 0.92 | 0.93 | 0.95 | 0.96 | 0.98 | 0.99 | 1.01 |
| **95% *CI*** | 0.86 – 0.95 | 0.89 – 0.96 | 0.91 – 0.96 | 0.93 – 0.96 | 0.95 – 0.98 | 0.95 – 1.00 | 0.96 – 1.02 | 0.96 – 1.05 |

For peer review only



For peer review only - http://bmjopen.bmj.com/site/about/guidelines.xhtml

**STROBE 2007 (v4) Statement—Checklist of items that should be included in reports of *cross-sectional studies***

| Section/Topic | Item # | Recommendation | Reported on page # |
|---|---|---|---|
| **Title and abstract** | 1 | (*a*) Indicate the study's design with a commonly used term in the title or the abstract | 1 |
| | | (*b*) Provide in the abstract an informative and balanced summary of what was done and what was found | 2 |
| **Introduction** | | | |
| Background/rationale | 2 | Explain the scientific background and rationale for the investigation being reported | 5-7 |
| Objectives | 3 | State specific objectives, including any prespecified hypotheses | 7 |
| **Methods** | | | |
| Study design | 4 | Present key elements of study design early in the paper | 7 |
| Setting | 5 | Describe the setting, locations, and relevant dates, including periods of recruitment, exposure, follow-up, and data collection | 7-9 |
| Participants | 6 | (*a*) Give the eligibility criteria, and the sources and methods of selection of participants | 8-9 |
| Variables | 7 | Clearly define all outcomes, exposures, predictors, potential confounders, and effect modifiers. Give diagnostic criteria, if applicable | 9 |
| Data sources/ measurement | 8* | For each variable of interest, give sources of data and details of methods of assessment (measurement). Describe comparability of assessment methods if there is more than one group | 9 |
| Bias | 9 | Describe any efforts to address potential sources of bias | 8 |
| Study size | 10 | Explain how the study size was arrived at | 13 |
| Quantitative variables | 11 | Explain how quantitative variables were handled in the analyses. If applicable, describe which groupings were chosen and why | 10, 11 |
| Statistical methods | 12 | (*a*) Describe all statistical methods, including those used to control for confounding | 10-13 |
| | | (*b*) Describe any methods used to examine subgroups and interactions | 12 |
| | | (*c*) Explain how missing data were addressed | 13 |
| | | (*d*) If applicable, describe analytical methods taking account of sampling strategy | - |
| | | (*e*) Describe any sensitivity analyses | 12, 15 |
| **Results** | | | |

For peer review only

| | | | |
|---|---|---|---|
| Participants | 13* | (a) Report numbers of individuals at each stage of study—eg numbers potentially eligible, examined for eligibility, confirmed eligible, included in the study, completing follow-up, and analysed | 13 |
| | | (b) Give reasons for non-participation at each stage | 13 |
| | | (c) Consider use of a flow diagram | - |
| Descriptive data | 14* | (a) Give characteristics of study participants (eg demographic, clinical, social) and information on exposures and potential confounders | 13-14 |
| | | (b) Indicate number of participants with missing data for each variable of interest | 13 |
| Outcome data | 15* | Report numbers of outcome events or summary measures | 14-15 |
| Main results | 16 | (*a*) Give unadjusted estimates and, if applicable, confounder-adjusted estimates and their precision (eg, 95% confidence interval). Make clear which confounders were adjusted for and why they were included | 14-18 |
| | | (*b*) Report category boundaries when continuous variables were categorized | - |
| | | (*c*) If relevant, consider translating estimates of relative risk into absolute risk for a meaningful time period | - |
| Other analyses | 17 | Report other analyses done—eg analyses of subgroups and interactions, and sensitivity analyses | 18-19 |
| **Discussion** | | | |
| Key results | 18 | Summarise key results with reference to study objectives | 19-20 |
| Limitations | 19 | Discuss limitations of the study, taking into account sources of potential bias or imprecision. Discuss both direction and magnitude of any potential bias | 22 |
| Interpretation | 20 | Give a cautious overall interpretation of results considering objectives, limitations, multiplicity of analyses, results from similar studies, and other relevant evidence | 20-22 |
| Generalisability | 21 | Discuss the generalisability (external validity) of the study results | 22 |
| **Other information** | | | |
| Funding | 22 | Give the source of funding and the role of the funders for the present study and, if applicable, for the original study on which the present article is based | 32 |

*Give information separately for cases and controls in case-control studies and, if applicable, for exposed and unexposed groups in cohort and cross-sectional studies.

**Note:** An Explanation and Elaboration article discusses each checklist item and gives methodological background and published examples of transparent reporting. The STROBE checklist is best used in conjunction with this article (freely available on the Web sites of PLoS Medicine at http://www.plosmedicine.org/, Annals of Internal Medicine at http://www.annals.org/, and Epidemiology at http://www.epidem.com/). Information on the STROBE Initiative is available at www.strobe-statement.org.

For peer review only

# Risk-adjusted trend in national inpatient fall rates observed from 2011 to 2019 in acute care hospitals in Switzerland: a repeated multicentre cross-sectional study



| | |
|---|---|
| Journal: | *BMJ Open* |
| Manuscript ID | bmjopen-2023-082417.R1 |
| Article Type: | Original research |
| Date Submitted by the Author: | 28-Feb-2024 |
| Complete List of Authors: | Bernet, Niklaus; Bern University of Applied Sciences, School of Health Professions<br>Everink, Irma; Maastricht University Care and Public Health Research Institute, Department of Health Services Research<br>Hahn, Sabine; Bern University of Applied Sciences, School of Health Professions<br>Müller, Marianne; Bern University of Applied Sciences, School of Health Professions<br>Schols, Jos; Maastricht University Care and Public Health Research Institute, Department of Health Services Research |
| <b>Primary Subject Heading</b>: | Health services research |
| Secondary Subject Heading: | Nursing, Health policy, Health services research |
| Keywords: | Hospitals, Nursing Care, Quality in health care < HEALTH SERVICES ADMINISTRATION & MANAGEMENT, Risk Factors |
| | |



SCHOLARONE™ Manuscripts

*I, the Submitting Author has the right to grant and does grant on behalf of all authors of the Work (as defined in the below author licence), an exclusive licence and/or a non-exclusive licence for contributions from authors who are: i) UK Crown employees; ii) where BMJ has agreed a CC-BY licence shall apply, and/or iii) in accordance with the terms applicable for US Federal Government officers or employees acting as part of their official duties; on a worldwide, perpetual, irrevocable, royalty-free basis to BMJ Publishing Group Ltd ("BMJ") its licensees and where the relevant Journal is co-owned by BMJ to the co-owners of the Journal, to publish the Work in this journal and any other BMJ products and to exploit all rights, as set out in our licence.*

*The Submitting Author accepts and understands that any supply made under these terms is made by BMJ to the Submitting Author unless you are acting as an employee on behalf of your employer or a postgraduate student of an affiliated institution which is paying any applicable article publishing charge ("APC") for Open Access articles. Where the Submitting Author wishes to make the Work available on an Open Access basis (and intends to pay the relevant APC), the terms of reuse of such Open Access shall be governed by a Creative Commons licence – details of these licences and which Creative Commons licence will apply to this Work are set out in our licence referred to above.*

*Other than as permitted in any relevant BMJ Author's Self Archiving Policies, I confirm this Work has not been accepted for publication elsewhere, is not being considered for publication elsewhere and does not duplicate material already published. I confirm all authors consent to publication of this Work and authorise the granting of this licence.*

For peer review only

# Risk-adjusted trend in national inpatient fall rates observed from 2011 to 2019 in acute care hospitals in Switzerland: a repeated multicentre cross-sectional study

**Author names:**

Niklaus S Bernet[a], Irma HJ Everink[b], Sabine Hahn[a], Marianne Müller[a], Jos MGA Schols[b]

**Affiliations**:

[a] Bern University of Applied Sciences, School of Health Professions, Applied Research & Development in Nursing, Murtenstrasse 10, 3008 Bern, Switzerland

niklausstefan.bernet@bfh.ch, sabine.hahn@bfh.ch, marianne.mueller@bfh.ch

[b] Maastricht University, Care and Public Health Research Institute, Department of Health Services Research, PO BOX 616, 6200 MD, Maastricht, the Netherlands

i.everink@maastrichtuniversity.nl, jos.schols@maastrichtuniversity.nl

**Corresponding author**:

Niklaus S Bernet, Bern University of Applied Sciences, School of Health Professions, Applied Research & Development in Nursing, Murtenstrasse 10, 3008 Bern, Switzerland, +41318484585, niklausstefan.bernet@bfh.ch

Word count of the main manuscript (introduction, methods, results, discussion, conclusions): 4488

For peer review only



For peer review only - http://bmjopen.bmj.com/site/about/guidelines.xhtml

# Abstract

*Objectives:* This study aimed to investigate whether a significant trend regarding inpatient falls in Swiss acute hospitals could be confirmed on a national level between 2011 and 2019, and whether the trend persists after risk adjustment for patient-related fall risk factors.

*Design:* A secondary data analysis was conducted based on annual multicentre cross-sectional studies carried out between 2011 and 2019.

*Setting:* All Swiss acute care hospitals were obliged to participate in the surveys. Except for emergency departments, outpatient wards and recovery rooms, all wards were included.

*Participants:* All inpatients aged 18 or older who had given their informed consent and whose data were complete and available were included.

*Main outcome measure:* Whether a patient had fallen in hospital was retrospectively determined on the survey day by asking patients the following question: Have you fallen in this institution in the last 30 days?

*Results:* Based on data from 110893 patients from 222 Swiss hospitals, a national inpatient fall rate of 3.7% was determined over the 9 survey years. A significant linear decreasing trend ($p$ = 0.004) was observed using the Cochran-Armitage trend test. After adjusting for patient-related fall risk factors in a two-level random intercept logistic regression model, a significant non-linear decreasing trend was found at the national level.

*Conclusions:* A significant decrease in fall rates in Swiss hospitals, indicating an improvement in the quality of care provided, could be confirmed both descriptively




For peer review only - http://bmjopen.bmj.com/site/about/guidelines.xhtml

For peer review only

and after risk adjustment. However, the non-linear trend, i.e. an initial decrease in inpatient falls that flattens out over time, also indicates a possible future increase in fall rates. Monitoring of falls in hospitals should be maintained at national level. Risk adjustment accounts for the observed increase in patient-related fall risk factors in hospitals, thus promoting a fairer comparison of the quality of care provided over time.

# Article summary

### Strengths and limitations of this study

- The internationally standardised primary data collection used in this study ensures high data quality in terms of completeness and uniformity, offering relevant advantages compared to the use of data generated for other purposes (e.g. a critical incidence reporting system).
- A comprehensive, nationally representative sample was used, comprising almost all Swiss hospitals with a somatic acute care mandate.
- The risk adjustment method used enables a fairer comparison of inpatient fall rates over time, as it controls for changes in patient-related fall risk factors.
- The cross-sectional survey design hinders consideration of the temporal sequence between inpatient fall risk factors and fall events, thereby limiting the recommended exclusive focus on pre-existing fall risk factors that cannot be influenced by hospital care in the risk adjustment model.

# Keywords

- Accidental falls
- Cross-Sectional Studies



For peer review only - http://bmjopen.bmj.com/site/about/guidelines.xhtml

72 • Hospitals
73 • Inpatients
74 • Quality Assurance
75 • Quality of Health Care
76 • Multilevel Analysis
77 • Risk Adjustment
78 • Trends

For peer review only



For peer review only - http://bmjopen.bmj.com/site/about/guidelines.xhtml

# Introduction

Regular national quality measurement in healthcare – the process of systematically measuring and reporting quality indicators at a national level to inform stakeholders at different levels of the health system – is assumed to drive quality improvement processes. This happens via two main pathways, as outlined by Berwick, et al. [1]. First, the "improvement by choice" pathway allows healthcare consumers to select providers based on publicly reported performance data. This incentivises providers to improve the quality of care due to market competition.[1 2] Second, the "improvement through change" pathway uses performance data to highlight areas where quality improvements are needed, fostering intrinsic motivation for providers to initiate improvements.[1 2] In addition, the fear of reputational damage from making performance data publicly available can be an additional incentive to improve the quality of healthcare services.[3 4]

National quality measurement is therefore a recognised strategy for monitoring and improving the quality of healthcare by national policy-makers.[3 5] Regular quality measurement provides the basis for initiating data-driven quality improvement strategies such as benchmarking and public reporting or the monitoring of achievements over time.[3 5] There is also some indication in the literature that these strategies can have a positive effect on the quality of care over time.[6 7] For example, a risk-adjusted trend analysis in the US between 2010 and 2019 showed a significant reduction in adverse events, particularly in hospitals that were affected by targeted quality improvement efforts during this period.[8]

One of the quality indicators that could be improved through regular national measurements and targeted quality improvement efforts is inpatient fall rate. Falls in hospital have serious consequences for both patients and the healthcare system as



For peer review only - http://bmjopen.bmj.com/site/about/guidelines.xhtml

a whole.[9 10] Based on data from Australia, a fall in hospital results, on average, in an 8-day longer hospital stay and (converted into international dollars according to the Organisation for Economic Co-operation and Development[11]) an additional cost of $4864.[12] Many inpatient falls, though not all, can be prevented with rigorous application of best-practice prevention measures.[13] Therefore, the number of falls in hospital is widely recognised as a relevant indicator of quality of care. Internationally, there are only a few organisations that we are aware of, such as the Press Ganey National Database of Nursing Quality Indicators (NDNQI)[14 15] or the International Prevalence Measurement of Care Quality (LPZ),[16 17] that provide a national infrastructure for collecting data on the quality indicator of falls in hospital.

Since 2011, the LPZ measurement has been conducted annually in Switzerland as a national mandatory quality measurement initiative, commissioned by the Swiss Association for Quality Development in Hospitals and Clinics (ANQ). Alongside other indicators, the prevalence of inpatient falls in all Swiss acute care hospitals is determined using cross-sectional primary data.[18] The aim is multifaceted: to inform a wide range of stakeholders – politicians, hospitals and the general public – about the findings and, particularly, to provide hospitals with the data needed to initiate quality improvement measures.[19] Data collected per measurement is forwarded to the Bern University of Applied Sciences (BFH) for comprehensive national analysis and reporting. The results will ultimately be published on the ANQ website (www.anq.ch), both in the form of a detailed report and a transparent national hospital comparison.[18-20] A recently submitted descriptive analysis based on this data from 2011 to 2019 showed that inpatient fall rates in Switzerland decreased substantially after the first survey and then stagnated over time.[21]



For peer review only - http://bmjopen.bmj.com/site/about/guidelines.xhtml

However, this putative trend could not be conclusively assessed purely on the basis of the descriptive analysis. A decreasing trend in fall rates at the national level might be an indication that the quality of care in hospitals with regard to fall prevention has improved over time. However, a stagnation of fall rates over time would contradict the hypothesis outlined by Berwick, et al. [1] that regular quality measurements favour the initiation of quality improvement processes and thus continuous quality improvement, which would be reflected in decreasing national inpatient fall rates over time. It is however also possible that there are other reasons that favour a stagnation of fall rates in the hospitals and thus possibly mask the effect of regular quality measurements on quality improvement in the hospitals.

- Not all falls are preventable: Even if fall prevention is continuously improved, the fall rates will remain constant at a certain level, as a reduction to zero will not be achievable.[13]
- Lack of resources to intensify fall prevention: Due to increasing financial constraints and staff shortages, fall prevention cannot be further strengthened.[22]
- Patient-related risk factors for falls are increasing: More and more often, older and multimorbid patients are being treated in the hospital, patients who carry a higher risk of falling. This means that more patients with a higher fall risk profile are being treated in hospital over time. As a result, falls in hospital do not decrease despite constant quality improvements.[21]

In the last scenario, keeping fall rates constant would still be a success.

In order to accurately compare inpatient fall rates over time while accounting for changing patient-related fall risk factors, it is usually recommended to conduct a risk-adjusted comparison.[23] Risk adjustment in this context means that a statistical



For peer review only - http://bmjopen.bmj.com/site/about/guidelines.xhtml

model is used to control for ongoing changes in patient-related fall risk factors that cannot be influenced by hospitals. By keeping patient-related fall risk factors constant over time, it can be ensured that positive/negative trends are actually due to quality improvements/deteriorations in care rather than to changes in patient-related risk factors.

The objectives of this study were therefore:

- First, to investigate whether a trend regarding the prevalence of inpatient falls in acute care hospitals can be statistically confirmed at a national level in Switzerland between 2011 and 2019.
- Second, whether an identified trend persists after risk adjustment for patient-related fall risk factors.

# Methods

## Study design

We conducted a secondary data analysis based on multicentre cross-sectional primary data collected annually between 2011 and 2019 as part of the LPZ measurement in Switzerland. This measurement is used to collect data on various quality indicators such as falls, pressure injuries and malnutrition in different countries (e.g. the Netherlands, Austria, Switzerland) during a defined data collection period. In Switzerland, usually on the Tuesday of the second week in November. The methods used in the so-called LPZ measurement are described in detail elsewhere.[17 18 24]

## Data collection and population



For peer review only - http://bmjopen.bmj.com/site/about/guidelines.xhtml

The LPZ questionnaire was used to collect the primary data.[17 18] This questionnaire emerged from an instrument developed on the basis of a literature review, a Delphi and a pilot study for the cross-setting and cross-institutional survey of pressure injuries,[25] which was assessed as reliable and valid.[25-27] Subsequently, other instruments were added to the initial questionnaire to capture further quality indicators, such as falls, by an international research group and based on evidence-based research. The LPZ questionnaire is divided into three parts: Institutional, ward and patient questionnaires. In 2016, it underwent a revision and was subsequently referred to as LPZ 2.0. This revision streamlined the questionnaire by reducing the number of structure and process indicators, while largely preserving the questions related to patient characteristics. Although the revision also affected the questions on quality indicators, these changes involved a reduction in the number of questions rather than a change in content. For instance, regarding the quality indicator on falls, specific context questions on the time and place of the fall and the main causes were deleted. Detailed descriptions of the adjustments made to the “inpatient fall” variable can be found in the paragraph “Outcome variable”.

Data collection in the hospitals was organised and coordinated by designated hospital coordinators, who were trained during annual national training events using the train-the-trainer principle, so that they could subsequently train their clinical data collection teams on site. To additionally ensure uniform data collection, a detailed measurement manual with definitions, instructions, explanations and examples was available to all persons involved. The clinical data collection teams each consisted of two nurses. They gathered the required data by directly questioning the patient or,



For peer review only - http://bmjopen.bmj.com/site/about/guidelines.xhtml

where permissible, from the patient's documentation on the survey day and then entered it into the web-based data entry programme.

In Switzerland, all acute care hospitals with an acute somatic service mandate have been obliged to collect data on at least pressure injuries and inpatient falls once a year in November since 2011. Other institutions were allowed to participate in the LPZ measurement on a voluntary basis. In 2020 and 2021, no LPZ measurement took place in Switzerland due to the Covid pandemic. At ward level, all wards were included, with the exception of emergency departments, outpatient wards and recovery rooms as of 2012, and additionally, maternity wards as of 2013. On the survey day, all inpatients were included in the LPZ measurement. In addition, written consent had to be obtained from patients or legal representatives for inclusion in 2011, and verbal consent from 2012 onwards (see ethics statements). The hospital coordinators were responsible for ensuring that all patients or, if they were unable to do so, their legal representatives were informed in advance in writing about the aim and procedure of the measurement. The written/oral consent of the patients was clarified directly at the time of the survey, that of the legal representatives in advance.

For the national analysis and reporting, the BFH adjusted the raw LPZ dataset for each measurement to fulfil the inclusion and exclusion criteria specified by ANQ for national reporting.

The following adjustments were made:

- Exclusion of institutions that do not have an acute somatic service mandate, such as psychiatric, rehabilitation and geriatric clinics.
- Exclusion of patients under the age of 18.



For peer review only - http://bmjopen.bmj.com/site/about/guidelines.xhtml

**Study population**

For the present study, we had full access to the national analysis datasets of the BFH from 2011 to 2019, to which the following general adjustments were made for the current secondary data analysis:

- Exclusion of conflicting cases.
- Exclusion of patients for whom it was unknown whether they had fallen in the institution in the last 30 days.
- Exclusion of non-participating patients from whom only the reason for non-participation was recorded.
- Exclusion of cases due to missing information on relevant patient-related fall risk variables.

See also supplementary figure S1 for a flowchart depicting the selection of cases for analysis.

**Outcome variable**

The outcome variable “inpatient fall” is defined in the LPZ measurement as “any unintentional change in position that results in the client coming to rest on the ground or other lower level, regardless of the reason”.[17 28]

To determine whether or not a fall occurred in the hospital, the assessment was carried out retrospectively by the clinical data collection teams on the day of the survey. The assessment process involved three steps: First, the patients were asked; second, the nurse in charge was consulted; and third, patient documentation was reviewed for any recorded falls.

The following question(s) with specified response categories had to be answered:



For peer review only - http://bmjopen.bmj.com/site/about/guidelines.xhtml

- From 2011 to 2015:

  "How often has the patient fallen in the last 30 days?" with responses categorized as once (1), twice (2), thrice (3), more than three times (4), unknown (5), and not fallen (6).

  "When was the most recent fall?" with responses categorized as before admission in current health care facility (1) or after admission (2).

- From 2016 to 2019, after revision of the LPZ questionnaire, the questions were simplified to:

  "Has the patient fallen in the last 30 days in this institution?" with responses being no (0), yes (1), and unknown (97).

Based on the raw variables, the outcome variable "inpatient fall" was calculated as follows for the present study:

- From 2011 to 2015:

  If participation equal to yes, the variable for the timing of the last fall was recoded as follows: Before admission (1) to 0, after admission (2) to 1 and missing responses to 0. In this way, a new variable was created indicating whether the patient had fallen (yes or no) in the last 30 days in this institution. In addition, due to the lack of information on whether the last fall occurred before or after admission to the institution, all cases with "unknown" (5) concerning the question on the frequency of falls in the last 30 days were excluded from the study sample.

- From 2016 to 2019:

  The new variable indicating whether the client had fallen (no or yes) in the last 30 days in this institution was created by recoding the original variable no (0) to 0, yes (1) to 1, and unknown (97) to missing. In addition, all cases with



For peer review only - http://bmjopen.bmj.com/site/about/guidelines.xhtml

missing values on the newly created variable were excluded from the study sample due to missing information on whether the patient had fallen in the last 30 days in this institution.

## Patient-related fall risk variables (covariates)

The LPZ questionnaires used between 2011 and 2019 were studied to identify potential patient-related characteristics that could be used for risk adjustment. To be considered as potential variables, they had to fulfil the following criteria:

- The variable was collected on all survey dates.
- The variable had not undergone any significant changes in content over the course of the survey years (in some cases, minor linguistic changes were accepted)

The following patient-related characteristics were identified as possible variables for risk adjustment: Age, sex, surgical procedure within 14 days prior to measurement day, care dependency according to the Care Dependency Scale (CDS), ICD-10 (International Statistical Classification of Diseases and Related Health Problems 10th Revision[29]) diagnosis groups. For more information on the variables used in this study, see Table 1.

*Table 1: Overview of the variables used in this study*

For peer review only



For peer review only - http://bmjopen.bmj.com/site/about/guidelines.xhtml

| **Outcome variable** | **Answer options** |
|---|---|
| Has the client fallen in the last 30 days in this institution | no [0] / yes [1] |
| **Patient-related variables** | **Answer options** |
| Age (in years) | scale |
| Sex | male [0] / female [1] |
| Surgical procedure within 14 days prior to measurement | no [0] / yes [1] |
| Care Dependency Scale (CDS)* | care independent (70–75) [0]<br>to a great extent independent (60–69) [1]<br>partially dependent (45–59) [2]<br>to a great extent dependent (25–44) [3]<br>completely dependent (15–24) [4] |
| ICD-10 – Diseases of the circulatory system | no [0] / yes [1] |
| ICD-10 – Diseases of the musculoskeletal system and connective tissue | no [0] / yes [1] |
| ICD-10 – Endocrine, nutritional and metabolic diseases | no [0] / yes [1] |
| ICD-10 – Diseases of the genitourinary system | no [0] / yes [1] |
| ICD-10 – Diseases of the digestive system | no [0] / yes [1] |
| ICD-10 – Diseases of the respiratory system | no [0] / yes [1] |
| ICD-10 – Neoplasms | no [0] / yes [1] |
| ICD-10 – Mental, behavioural and neurodevelopmental disorders | no [0] / yes [1] |
| ICD-10 – Diseases of the blood and blood-forming organs | no [0] / yes [1] |
| ICD-10 – Diseases of the nervous system | no [0] / yes [1] |
| ICD-10 – Certain infectious and parasitic diseases | no [0] / yes [1] |
| ICD-10 – Diseases of the skin and subcutaneous tissue | no [0] / yes [1] |
| ICD-10 – Injury, poisoning and certain other consequences of external causes | no [0] / yes [1] |
| ICD-10 – Diseases of the eye and adnexa AND ICD-10 – Diseases of the ear and mastoid process** | no [0] / yes [1] |
| ICD-10 – Congenital malformations, deformations and chromosomal abnormalities | no [0] / yes [1] |
| **Time variable** | **Values** |
| Time (survey year) | 2011 [1] / 2012 [2] / 2013 [3] / 2014 [4] / 2015 [5] / 2016 [6] / 2017 [7] / 2018 [8] / 2019 [9] |

For peer review only

* The Care Dependency Scale (CDS) is composed of 15 questions, which are rated on a scale from 1 to 5. The total score ranges from 15 to 75 points, whereby a lower score indicates higher care dependency.[30 31] The following five categories can be derived from the sum score: completely dependent on care from others, to a great extent dependent, partially dependent, to a great extent independent and care independent.[32 33]

** These ICD-10 diagnosis groups were only recorded separately from 2013 onwards and were therefore combined for the analyses in order to standardise them.

## Data analysis

The datasets from each of the nine survey years were prepared by standardizing the variable names, descriptions, and labels. Following this, case exclusion criteria were applied consistently as detailed in the flowchart in supplementary figure S1, and the outcome variable was calculated uniformly across all datasets. After merging the



For peer review only - http://bmjopen.bmj.com/site/about/guidelines.xhtml

datasets into one overall dataset by adding a time variable, the sample was first described by means of frequencies and percentages and age by means of median and interquartile range (IQR) due to the skewed distribution. Furthermore, the national prevalence of falls in hospital between 2011 and 2019 was estimated as described by Thomann, et al. [20]. For this purpose, based on the dataset for the corresponding year, the number of patients with a fall in hospital was divided by the total number of patients in the dataset and then multiplied by 100 to obtain the inpatient fall rate as a relative frequency in per cent.

Second, to investigate whether a trend in the prevalence of inpatient falls in acute care hospitals could be statistically confirmed at the national level in Switzerland between 2011 and 2019, the Cochrane-Armitage trend test was used. This test is suitable for identifying a linear trend in a binary variable, where the null hypothesis is that there is no trend,[34] i.e., that fall rates have remained the same over time. For purposes of visualisation, a linear trend line was plotted based on a linear regression.

Third, to determine whether an identified trend persists after risk adjustment for patient-related fall risk factors, we performed the following steps: a to d (risk adjustment model development) and e to f (reporting and visualisation).

a) As a starting point for setting up the risk adjustment model, we performed a logistic regression resulting in model "A", which included all available patient-related variables (see Table 1) as covariates and, to depict a trend, the time variable as a numerical covariate (coded from 1 to 9, where 1 denotes survey year 2011 and 9 survey year 2019). As it is rather unlikely that an increase or decrease will remain constant over the years, a quadratic time-effect was also included in model "A" in order to be able to take account of a non-linear



For peer review only - http://bmjopen.bmj.com/site/about/guidelines.xhtml

relationship. Additionally we tested for interaction effects between patient-related fall risk variables and the time variable.

b) A stepwise backward variable selection algorithm based on the Akaike Information Criterion (AIC)[35] was then applied to model "A" to determine the relevant patient-related fall risk factors to be included in the risk adjustment model and to check for the presence of a linear or a non-linear trend. The resulting model was designated as model "B".

c) Since the data have a hierarchical structure (patients grouped in hospitals), the selected variables according to model "B" were modelled as fixed effects and the hospitals as a random effect in a two-level random intercept logistic regression model (model "C"). Although the sample comprised almost the entire population of hospitals, a random effects approach is recommended as this allows the estimation of model parameters instead of finite population characteristics.[36]

d) To determine whether the model complexity of model "C" could be further reduced, a further two-level random intercept logistic regression model (model "D") was calculated. In model "D", the hospitals were modelled as a random effect and the statistically significant time-related factors as well as the patient-related fall risk factors of model "C" were included as fixed effects. If the ANOVA test revealed no significant difference in the model fit between model "C" and the reduced model "D", model "D" served as the final risk adjustment model that informed the subsequent steps e to f.

e) To determine whether the patient-related fall risk factors have changed over time and therefore to gain an idea of whether risk adjustment is necessary at all, the patient-related fall risk factors selected in model "D" (without time-



For peer review only - http://bmjopen.bmj.com/site/about/guidelines.xhtml

related factors) were included as covariates in a two-level random intercept logistic regression model (model “E”). The need for risk adjustment is indicated if the patient-related fall risk factors have increased or decreased over time. This can be recognised if the national inpatient fall rates per survey year increase/decrease if they are predicted solely on the basis of the selected patient-related fall risk factors. The risk-adjusted national inpatient fall rates predicted based on model “E” were graphically plotted over time for visualisation purposes.

f) Finally, to demonstrate the predicted risk-adjusted trend in national fall rates in hospitals over time, the predicted risk-adjusted trend was graphically plotted on the basis of model "D". By controlling for the average effect of patient-related fall risk factors over time, changes in these factors can be largely excluded as a reason for a significant increasing/decreasing trend in the predicted national fall rates. Therefore, an increase/decrease, if any, would more likely be due to a deterioration/improvement in the quality of care provided in hospitals over time.

Fourth, an additional analysis was conducted by repeating the analyses on a subsample of patients aged 65 years and older. This subsample was selected because older patients in particular are more often subject to falls in hospital.

Fifth, as part of a sensitivity analysis, we repeated the complete data analysis using only hospitals that provided data in all nine survey years to assess for potential bias in the results from hospitals that had joined or left the measurement during the study period. This approach was based on the assumption that the introduction of annual national quality measurement could trigger quality improvement initiatives in participating hospitals, potentially reducing fall rates over time. However, this trend



For peer review only - http://bmjopen.bmj.com/site/about/guidelines.xhtml

could be masked by newer hospitals participating in the measurement, as they may initially have higher fall rates compared to hospitals already participating. The aim of the sensitivity analysis was therefore to detect such a masking effect, if one existed.

Data cleaning and merging of the datasets was carried out with IBM© SPSS© Statistics (version 28). All analyses and graphics were carried out with the statistics programme R, version 4.1.0[37] and the packages “DescTools”,[38] “MASS”,[39] “lme4”,[40] “effects”[41 42] and “sjplot”[43]. In the analyses, a p-value < .05 was set as the statistical significance level.

# Results

*Sample description*

A total of 155782 inpatients were recorded in 224 Swiss acute care hospitals on the survey days over the 9 survey years. Of these, a total of 110893 patients were included in the present study sample, yielding an average case inclusion rate of 71.2%. The case inclusion rate was lowest in 2011 with 36.1% and highest in 2016 with 76.4%. The primary reasons for case exclusions were patient non-participation due to refusal, unavailability at the time of survey or cognitive impairment. Additionally, missing information on care dependency led to the exclusion of 4726 patients in 2011. Therefore, the results presented below are based on a total of 110893 cases from 222 hospitals. Patient characteristics of the sample are described overall in Table 2 and by survey year in the supplementary table S1.

*Table 2: Description of the patient characteristics of the sample*

| **Patient characteristics** | *n* = 110893 | % |
|---|---|---|
| Sex [female] | 56015 | 50.5 |
| Care dependency (CDS) | | |
| care independent [70-75] | 59875 | 54.0 |



For peer review only

For peer review only - http://bmjopen.bmj.com/site/about/guidelines.xhtml

| | | |
|---|---|---|
| to a great extent independent [60-69] | 25970 | 23.4 |
| partially dependent [45-59] | 15508 | 14.0 |
| to a great extent dependent [25-44] | 6992 | 6.3 |
| completely dependent [15-24] | 2548 | 2.3 |
| Surgical procedure within 14 days prior to measurement [yes] | 47233 | 42.6 |
| ICD-10 – Diseases of the circulatory system [yes] | 58987 | 53.2 |
| ICD-10 – Diseases of the musculoskeletal system and connective tissue [yes] | 42349 | 38.2 |
| ICD-10 – Endocrine, nutritional and metabolic diseases [yes] | 34244 | 30.9 |
| ICD-10 – Diseases of the genitourinary system [yes] | 32079 | 28.9 |
| ICD-10 – Diseases of the digestive system [yes] | 27884 | 25.1 |
| ICD-10 – Diseases of the respiratory system [yes] | 25600 | 23.1 |
| ICD-10 – Neoplasms [yes] | 21167 | 19.1 |
| ICD-10 – Mental, behavioural and neurodevelopmental disorders [yes] | 19827 | 17.9 |
| ICD-10 – Diseases of the blood and blood-forming organs [yes] | 15461 | 13.9 |
| ICD-10 – Diseases of the nervous system [yes] | 13494 | 12.2 |
| ICD-10 – Certain infectious and parasitic diseases [yes] | 13494 | 12.2 |
| ICD-10 – Diseases of the eye and adnexa OR ICD-10 – Diseases of the ear and mastoid process [yes] | 8148 | 7.3 |
| ICD-10 – Diseases of the skin and subcutaneous tissue [yes] | 7572 | 6.8 |
| ICD-10 – Injury, poisoning and certain other consequences of external causes [yes] | 7559 | 6.8 |
| ICD-10 – Congenital malformations, deformations and chromosomal abnormalities [yes] | 625 | 0.6 |
| | Median | IQR |
| Age [in years] | 70 | 24 |

*Unadjusted trend in national inpatient fall rates*

The national inpatient fall rate over the 9 years of the survey was 3.7%. As Figure 1 shows, the national inpatient fall rate was highest in 2011, at 4.6%, and lowest in 2015, at 3.0%. At the last survey in 2019, the fall rate was 3.7%. Using the Cochran-Armitage trend test, a significant linear decreasing trend over time was found (*p* for trend = 0.004). This means that the national fall rate decreased linearly over time when patient-related fall risk factors were not adjusted for. The estimated linear trend line (see also Figure 1) intersects the intercept y at a predicted value of 4.15% in 2011 and reaches a predicted value of 3.41% in 2019.



For peer review only - http://bmjopen.bmj.com/site/about/guidelines.xhtml

The national inpatient fall rate in 2015 deviates from the rates of the other survey years. In a sensitivity analysis we excluded the data from year 2015 and checked whether the results of the Cochran-Armitage trend test would become significantly different or not. Since a significant linear decreasing trend could be found both with and without the data from 2015, these were retained in the following analyses.

*Risk-adjusted trend of Swiss national inpatient fall rate*

When patient-related fall risk factors were controlled for, a statistically significant non-linear trend, indicated by the negative coefficient (coeff.) of the time variable (-0.10, *SE* 0.03, *p*=0.001) and the positive coefficient of the squared time variable (0.01, *SE* 0.00, *p*=0.043) in the model, was observed in national inpatient fall rates (Table 3). The model indicates that the relationship between time and inpatient falls at national level is not linear, as the decreasing trend flattens out over time. The calculated odds ratios (supplementary table S2) illustrate the observed non-linear trend in that, for example, at the national level, the odds of falling in hospital decreased by 8% in 2012 compared to 2011 (*OR* 0.92, 95% *CI* 0.88–0.96). In contrast, however, the odds neither decreased nor increased in 2019 relative to 2018 (*OR* 1.00, 95% *CI* 0.96–1.04).

The trend described above was observed while controlling for a total of ten patient-related fall risk variables in the risk adjustment model (Table 3): eight significantly *fall risk-increasing* patient-related variables, such as age (*OR* 1.02, 95% *CI* 1.01–1.02, $p < 0.001$), being heavily care dependent (*OR* 5.69, 95% *CI* 5.10–6.36, $p < 0.001$) or in the ICD-10 diagnosis group Mental and Behavioural disorders (*OR* 1.79, 95% *CI* 1.66–1.92, $p < 0.001$); two significantly *fall risk-reducing* patient-related characteristics, sex (*OR* 0.82, 95% *CI* 0.77–0.87, $p < 0.001$) and surgery in the last 14 days prior to measurement (*OR* 0.61, 95% *CI* 0.57–0.66, $p < 0.001$).



For peer review only - http://bmjopen.bmj.com/site/about/guidelines.xhtml

*Table 3: Overview of the two-level random intercept logistic regression model used to derive the risk-adjusted trend in Swiss national inpatient fall rates*

| | Risk-adjusted trend in national inpatient fall rates | | | | |
|---|---|---|---|---|---|
| *Predictors* | *coeff.* | *SE* | *p-value* | *OR* | *95% CI* |
| (Intercept) | -5.00 | 0.12 | **<0.001** | - | - |
| **Time related factors (trend)** | | | | | |
| Time | -0.10 | 0.03 | **0.001** | - | - |
| (Time)^2 | 0.01 | 0.00 | **0.043** | - | - |
| **Patient related fall risk factors** | | | | | |
| Age [in years] | 0.02 | 0.00 | **<0.001** | 1.02 | 1.01 – 1.02 |
| CDS [care independent (70-75)] | Ref. | | | | |
| CDS [to a great extent independent (60-69)] | 1.04 | 0.05 | **<0.001** | 2.83 | 2.57 – 3.11 |
| CDS [partially dependent (45-59)] | 1.42 | 0.05 | **<0.001** | 4.15 | 3.76 – 4.58 |
| CDS [to a great extent dependent (25-44)] | 1.74 | 0.06 | **<0.001** | 5.69 | 5.10 – 6.36 |
| CDS [completely dependent (15-24)] | 1.32 | 0.09 | **<0.001** | 3.73 | 3.15 – 4.42 |
| ICD-10 – Mental and Behavioural disorders [yes] | 0.58 | 0.04 | **<0.001** | 1.79 | 1.66 – 1.92 |
| ICD-10 – Neoplasms [yes] | 0.37 | 0.04 | **<0.001** | 1.45 | 1.35 – 1.56 |
| ICD-10 – Diseases of the nervous system [yes] | 0.28 | 0.04 | **<0.001** | 1.32 | 1.22 – 1.44 |
| ICD-10 – Diseases of the blood and blood-forming organs [yes] | 0.21 | 0.04 | **<0.001** | 1.23 | 1.13 – 1.33 |
| ICD-10 – Injury, poisoning, other consequences of external causes [yes] | 0.15 | 0.06 | **0.008** | 1.16 | 1.04 – 1.30 |
| ICD-10 – Endocrine, nutritional and metabolic diseases [yes] | 0.13 | 0.03 | **<0.001** | 1.13 | 1.06 – 1.21 |
| Sex [female] | -0.20 | 0.03 | **<0.001** | 0.82 | 0.77 – 0.87 |
| Surgical procedure within 14 days prior to measurement [yes] | -0.49 | 0.04 | **<0.001** | 0.61 | 0.57 – 0.66 |
| **Random Effects** | | | | | |
| $\tau_{00}$ [variability in hospital intercepts] | | | 0.08 | | |
| N [hospitals] | | | 222 | | |
| Observations | | | 110893 | | |

Significant *p*-values are highlighted in bold.

Figure 2 illustrates the predicted national fall rates per survey year when the following are adjusted for: a) only patient-related fall risk factors (red line), or, b) patient-related fall risk factors as well as a non-linear time trend (green line). The red line shows that patient-related fall risk factors have tended to increase over time, which is reflected in increasing predicted risk-adjusted national fall rates. These are estimated to be 3.65% in year 2011 and 4.04% in year 2019. The increase in patient-related fall risk factors over time is also evident from the supplementary table S1. For 8 of the 10 variables included in the risk adjustment model, it was shown descriptively that the patient-related fall risk variables have increased over the years, reaching the highest values in 2019 and the lowest values for female sex and



For peer review only - http://bmjopen.bmj.com/site/about/guidelines.xhtml

percentage of completely care-independent patients in 2019. For example, the ICD-10 diagnosis group “Mental, behavioural and neurodevelopmental disorders” increased from 15.6% in 2011 to 20.5% in 2019. This underscores the necessity of risk adjustment. The green line shows a decrease followed by a slight increase in predicted national inpatient fall rates over time when changes in patient-related fall risk factors as well as a non-linear time trend are adjusted for. The predicted fall rates based on this model are highest at 4.44% in 2011, lowest at 3.43% in 2016 and are 3.73% in 2019. As none of the 95% confidence intervals of the calculated odds ratios (supplementary table S2) are above 1, the slight increase observed visually is not statistically significant.

In the additional analysis, a subsample consisting of 67336 patients aged 65 years or older from 221 hospitals was considered. The results based on this subsample do not differ significantly from the overall sample: Although the national fall rates are descriptively consistently higher than in the overall sample, the same patient-related fall risk variables were selected into the risk adjustment model and the coefficients of the risk variables in the model were not substantially different (see supplementary table S3, table S4, figure S2 and figure S3).

*Sensitivity analysis*

The sensitivity analysis included 93 hospitals that provided complete data over all nine survey years, with a total of 64038 patients. In contrast to the overall sample, the results based on this subsample show a significant linear decreasing trend in the inpatient fall rates over time, both descriptively and risk-adjusted (supplementary figure S4, supplementary table S5 and supplementary figure S5). Although the quadratic time effect was included in the model during AIC selection, the effect was not significant and was therefore omitted from the final model. In the risk adjustment



For peer review only - http://bmjopen.bmj.com/site/about/guidelines.xhtml

For peer review only

model, with the exception of the additionally selected variable "ICD-10 - diseases of the circulatory system", the same patient-related fall risk variables were included in the model, and the coefficients of the risk variables in the model did not differ significantly from the model based on the overall sample (supplementary table S5).

# Discussion

In this study, we investigated whether inpatient fall rates in acute care hospitals in Switzerland have changed at the national level between 2011 and 2019, and whether these changes persist when patient-related fall risk factors are adjusted for. In the unadjusted analysis, a linear decreasing trend in inpatient fall rates at the national level was found across the years surveyed. In contrast, the risk-adjusted analysis (which controlled for the average effect of patient-related factors) revealed a significant non-linear decreasing trend across the same years. In other words, the effect appears to flatten out over time. Similar results, i.e., a non-adjusted decreasing trend in fall rates, were found in studies based on National Database of Nursing Quality Indicators (NDNQI) data in US hospitals over a 27-month as well as six-year time period.[44 45] More recently, another study found that combined inpatient falls and pressure injury rates in the US decreased statistically significantly from 2010 to 2019 after risk adjustment.[8]

It is assumed that the negative trend in risk-adjusted national fall rates observed in our study between 2011 and 2019 is largely due to continuous quality improvement of structures and processes for fall prevention in hospitals. This assumption is reinforced by a study which showed that improvements designed to prevent falls were implemented at both the structural and process level in Swiss hospitals during the same time period.[21] For example, the average relative frequency of the



For peer review only - http://bmjopen.bmj.com/site/about/guidelines.xhtml

number of fall prevention measures applied per patient, such as the evaluation of current medication or the evaluation of aid devices, increased significantly from 12.9% in 2011 to 23.1% in 2019.[21] The impetus for the improvement measures put in place by hospitals is likely to have been facilitated by the well-known quality improvement mechanisms associated with the introduction of national inpatient falls measurement in 2011. Although regular quality measurement alone does not automatically lead to quality improvement,[5] accompanying measures such as public reporting,[6 46] comparative benchmarking[7] and performance feedback via quality dashboard[47 48] are described as effective in triggering quality improvement activities in hospitals. The results of the present sensitivity analysis could also fit into this picture, in that hospitals that provided data on all nine survey years were more likely to have succeeded in continuously reducing their fall rates over time compared to the overall sample. It is possible that the regularity of quality measurement has led to a steady impetus to implement internal quality improvement measures. At least anecdotally, we are aware of some internal hospital projects aimed at reducing falls, which were initiated based on the results of the national quality measurement. Although it cannot be ruled out that other large-scale initiatives or programmes besides the introduction of the national quality measurement may have contributed to the reduction of falls in hospitals in Switzerland between 2011 and 2019, we are only aware of two fall prevention programmes of national scope implemented either before 2011 or after 2019 led by national organisations.[49 50]

Nevertheless, despite the quality improvement measures implemented by hospitals to prevent falls, the risk-adjusted national fall rates did not, as a whole, decline linearly over time. Instead, they showed a greater decline at the beginning of the surveys and then gradually levelled off, as demonstrated by the significant non-



For peer review only - http://bmjopen.bmj.com/site/about/guidelines.xhtml

linear effect observed in our study. This pattern was also observed for nosocomial pressure injuries, another quality indicator of patient safety, at least descriptively over time.[16 21 51 52] For example, a decreasing trend that levelled off over time was observed in hospitals in the USA between 2006 and 2019[51] and in Switzerland between 2011 and 2019.[21 52] Therefore, the trend found in our study may not be specific to falls, but may reflect a more general pattern. In principle, however, we can only speculate about the reasons for the non-linear trend found. Generally, it can be assumed that the lower fall rates get, the more difficult it becomes to reduce them further, as not all fall events are preventable.[13] Another explanation could be that, given the increase in patient-related fall risk factors over time, more and more effort is required on the part of hospitals just to keep fall rates constant and, accordingly, it becomes increasingly challenging to reduce them further.[21] This possible link is particularly emphasised by the results of the present study, which showed an increase in patient-related fall risk factors over time based on increasing predicted fall rates at national level when prediction is based on these patient-related fall risk factors (as shown by the red line in Figure 2). The observation that medical progress may unintentionally be contributing to a rise in risk factors and a concomitant levelling off of positive trends by enabling certain multimorbid patients to survive has already been made with regard to pressure injuries.[21 51]

Almost more important than identifying the reasons for the non-linear trend is the fact that it indicates a potential increase in fall rates at national level in the coming years if the observed trend continues. This development could be exacerbated by a further increase in fall risk factors and patient complexity in hospital, as well as by a shortage of nursing staff, especially registered nurses, which is expected to worsen



For peer review only - http://bmjopen.bmj.com/site/about/guidelines.xhtml

in the coming years both internationally[22] and in Switzerland[53]. A shortage of registered nurses has already been linked in a meta-analysis to poorer overall patient outcomes[54] and, in an observational study, to higher fall rates in hospital.[55] Under these circumstances, it is strongly recommended that inpatient fall rates should be continuously monitored. On the one hand, to inform decision-makers at all levels and health professionals about potential deteriorations in the quality of care provided and to enable initiation of appropriate data-driven countermeasures, and on the other hand, to investigate whether the non-linear effect found is confirmed in the future or whether it represents a sample-specific observation.

It should be noted that due to the large sample in our study even small differences become statistically significant. Therefore, as in the study by Bouldin, et al. [44], the question arises whether the observed decline in fall rates over time is also value-based. In our study, we found an absolute reduction in predicted risk-adjusted fall rates of 0.71% between 2011 and 2019. Extrapolated to the slightly more than 1.2 million cases in general hospitals in 2019 in Switzerland,[56] this means that the quality improvement measures implemented over the years prevented 8520 fall events in 2019 compared to 2011. This decrease may seem small, but considering the possible impact of a fall on the individual, such as injury or death,[57] this decrease is nevertheless remarkable and of great value to patients. The decrease is also valuable from an economic point of view. If we multiply the prevented fall events by 4864 international dollars, the average cost of a fall calculated by Morello, et al. [12], it is estimated that more than 41 million international dollars in healthcare costs could have been saved nationwide in 2019. These savings underscore the economic benefits of investing in fall prevention strategies, for which a good return



For peer review only - http://bmjopen.bmj.com/site/about/guidelines.xhtml

For peer review only

on investment is assumed.[10] Therefore, the implementation of evidence-based interventions, such as patient and staff education, could help to reduce fall rates, or at least keep them constant.[58] This would not only have a positive impact on the direct cost savings already mentioned, but also on the possibility of reallocating resources to other important areas of the healthcare system.

*Strength and Limitations*

A strength of our study is that the data used was collected using a highly standardised, internationally proven method and based on a census survey of all Swiss hospitals; it thus accurately reflects the situation at the national level. However, due to the average case inclusion rate of around 71% and the comparatively low rate of 36.1% in 2011, a possible selection bias in the LPZ measurement and in our study cannot be ruled out. Likewise, a recall bias cannot be ruled out either, since the patients were asked whether they had fallen in hospital in the last 30 days. As with the selection bias, the recall bias could have led to a potential underestimation of the fall rates observed. Since it can be assumed that the underestimation, if present, has remained constant over time, i.e. a systematic underestimation, this would have no influence on the trend found. In general, it should be noted that adjustments can only be made for measurable risk factors for which data are available. Due to the limited number of patient-related fall risk factors collected with the LPZ questionnaire in Switzerland, not all potentially relevant risk factors could be considered in the risk adjustment model. For instance, it should be investigated whether it is possible to reliably collect further data, for example, on frailty,[59] sarcopenia,[60] malnutrition,[61] impaired mobility,[62] urinary incontinence,[63] polypharmacy,[64] or use of cardiovascular,[65] psychotropic,[66] opioid,[64] antiepileptic[64] medication at national level without great effort. The



For peer review only - http://bmjopen.bmj.com/site/about/guidelines.xhtml

effects of integrating some of these additional risk factors on the risk adjustment model would also need to be investigated. In addition, due to adjustments in the LPZ questionnaire over time, two relevant fall risk variables, a fall in the last 12 months or the use of sedatives/psychotropic medication (only collected from 2016 onwards) could not be taken into account in the risk adjustment model. Although these variables were not available for the present analyses, on the whole, the same patient-related characteristics with similar coefficients were identified as fall risk variables as in our previous studies based on LPZ data.[18 67] Due to the cross-sectional survey design, a temporal link between the fall risk factors and the outcome is not possible. To enable this link and reduce selection bias, it is recommended that future trend analyses should look for opportunities to draw on data from the entire patient population where possible. Ideally, this data should cover the entire period from admission to discharge and, where possible, the variables used for risk adjustment should relate to patient-related fall risk factors that were already present on admission. In this respect, electronic medical record data could in future provide a suitable data basis for national monitoring of inpatient falls over time, including risk adjustment.[68] However, further research is required.

## Conclusions

In our study, we found, descriptively, a significant linear decreasing trend in fall rates in Swiss hospitals and, after adjustment for patient-related fall risk factors, a significant non-linear decreasing trend, i.e., a decrease that flattens out over time. It can be assumed that the introduction of the annual national measurement of inpatient falls in 2011 triggered improvement measures in hospitals in Switzerland, which in turn manifested in lower fall rates over time. The quality improvement

For peer review only



For peer review only - http://bmjopen.bmj.com/site/about/guidelines.xhtml

achieved has had positive effects both for patients and for society as a whole by improving health and saving costs. However, the identified non-linear effect also showed that the fall rates decreased more at the beginning of the measurements than later on. This could be an indication that fall rates may increase in the future, especially in light of future challenges such as increasing fall risk factors, as shown in our study, and the staff shortages that hospitals are facing. The extent to which the observed non-linear trend will be confirmed in the future requires further investigation. In this context, it is recommended that national policy makers continue to collect and monitor the fall rates in hospitals, on the one hand because this enables data-based quality development in the hospitals, and on the other so that a possible increase in national fall rates can be recognised at an early stage and appropriate targeted quality-improvement measures can be planned and implemented at national level. For this, robust, reliable and fair comparisons over time are important. Risk adjustment can be recommended as a method for improving the comparability of results over time. To address the limitations of the present study, it is also recommended to further investigate to what extent routine clinical data are suitable as a basis for national comparisons of fall rates, including risk adjustment for patient-related fall risk factors to ensure comparability over time.

For peer review only

## Acknowledgements

We would like to thank all hospitals and patients who participated in the surveys.

## Competing interests

None declared.



For peer review only - http://bmjopen.bmj.com/site/about/guidelines.xhtml

# Funding

The annual national measurement of inpatient falls in Switzerland and thus the data collection was financed by the Swiss National Association for Quality Development in Hospitals and Clinics (ANQ) and carried out under mandate by the Bern University of Applied Sciences (BFH). The present data analysis was also financed by ANQ (Contract no. "Kompensationsleistungen 2020").

# Data availability statement

The data on which the present results are based can be obtained on reasonable request from the Swiss National Association for Quality Development in Hospitals and Clinics (ANQ) (see www.anq.ch). However, the availability of these data may be subject to restrictions in accordance with the "Empfehlungen Verwendung von ANQ-Daten zu Forschungszwecken [Recommendations for the use of ANQ data for research purposes]", which can be found at www.anq.ch.

# Ethics statements

## Patient consent for publication

Not applicable.

## Ethics approval

This study was conducted in accordance with the ethical principles of the Declaration of Helsinki. Full Research Ethics Committee approval for the primary data collection was granted by the lead Cantonal Ethics Committee Bern (Kantonale Ethikkommission Bern) on 4 October 2011 (application no. 122/11) in agreement with the other twelve local cantonal ethics committees:



For peer review only - http://bmjopen.bmj.com/site/about/guidelines.xhtml

- Kantonale Ethikkommission Thurgau
- Kantonale Ethikkommission Luzern
- Kantonale Ethikkommission Aargau
- Kantonale Ethikkommission Zürich
- Ethikkommission beider Basel
- Ethikkommission des Kantons St. Gallen
- Ethikkommission Appenzell Ausserhoden (AR)
- Commission d'éthique pour la recherche clinique en ambulatoire dans le canton de Genève
- Commission d'éthique de recherche du canton de Fribourg
- Commission cantonale valaisanne d'éthique médicale
- Commission cantonale d'éthique de la recherche sur l'être humain
- Comitato etico cantonale Ticino.

As of 2012, Swiss ethics (Swiss Association of Research Ethics Committees) and the cantonal ethics committees declared the measurement as a quality measurement, which does not require ethical approval because it is not subjected to the Swiss Human Research Act. In the course of the reclassification of the measurement as a quality measurement, the ethics committees also agreed that written patient consent is no longer required and therefore written patient information followed by oral consent from the patients or their legal representatives is sufficient. Therefore, all patients or their legal representatives gave at least their verbal informed consent to participate in the measurement (see Bernet, et al. [18] for more details). Furthermore, in 2022, the Ethics Advisory Board of the Bern University of Applied Sciences declared that the anonymised data from the LPZ measurement



For peer review only - http://bmjopen.bmj.com/site/about/guidelines.xhtml

could be used again without further approval from a cantonal ethics committee (Ref. EAB2022_012). Only fully anonymised data were used in the present study.

## Contributors

Conceptualization of the measurement, NB, IE, SH, JS; conceptualization of the manuscript, NB, MM; data collection and curation, NB, MM; data analysis, NB, MM; data interpretation, NB, IE, SH, MM, JS; writing – original draft preparation, NB; writing – review and editing, IE, SH, MM, JS.

NB is responsible for the overall content of the manuscript as guarantor.

## Patient and public involvement

Patients and/or the public were not involved in the design, or conduct, or reporting or dissemination plans of this research.

## References

1. Berwick DM, James B, Coye MJ. Connections Between Quality Measurement and Improvement. *Medical Care* 2003;41(1):I-30-I-38. doi: https://doi.org/10.1097/00005650-200301001-00004
2. Smith PC, Mossialos E, Papanicolas I, et al. Performance Measurement for Health System Improvement: Experiences, Challenges and Prospects. Cambridge: Cambridge University Press 2010.
3. Busse R, Klazinga N, Panteli D, et al. *Improving healthcare quality in Europe: Characteristics, effectiveness and implementation of different strategies.* OECD WHOa, 2019. Accessed May 17, 2021. https://apps.who.int/iris/bitstream/handle/10665/327356/9789289051750-eng.pdf?sequence=1&isAllowed=y.
4. Hibbard JH, Stockard J, Tusler M. Hospital Performance Reports: Impact On Quality, Market Share, And Reputation. *Health Affairs* 2005;24(4):1150-60. doi: https://doi.org/10.1377/hlthaff.24.4.1150
5. Agweyu A, Hill K, Diaz T, et al. Regular measurement is essential but insufficient to improve quality of healthcare. *BMJ* 2023;380:e073412. doi: https://doi.org/10.1136/bmj-2022-073412
6. Metcalfe D, Rios Diaz AJ, Olufajo OA, et al. Impact of public release of performance data on the behaviour of healthcare consumers and providers. *Cochrane Database of Systematic Reviews* 2018(9) doi: https://doi.org/10.1002/14651858.CD004538.pub3



For peer review only - http://bmjopen.bmj.com/site/about/guidelines.xhtml

7. Willmington C, Belardi P, Murante AM, et al. The contribution of benchmarking to quality improvement in healthcare. A systematic literature review. *BMC Health Services Research* 2022;22(1):139. doi: https://doi.org/10.1186/s12913-022-07467-8
8. Eldridge N, Wang Y, Metersky M, et al. Trends in Adverse Event Rates in Hospitalized Patients, 2010-2019. *Jama* 2022;328(2):173-83. doi: https://doi.org/10.1001/jama.2022.9600
9. LeLaurin JH, Shorr RI. Preventing Falls in Hospitalized Patients: State of the Science. *Clinics in geriatric medicine* 2019;35(2):273-83. doi: https://doi.org/10.1016/j.cger.2019.01.007
10. Slawomirski L, Klazinga N. *The economics of patient safety.* Paris: OECD Publishing, 2022. Accessed February 23, 2024. https://doi.org/10.1787/761f2da8-en.
11. Organisation for Economic Co-operation and Development (OECD). Purchasing power parities (PPP) (indicator) 2024 [Available from: https://doi.org/10.1787/1290ee5a-en accessed February 18, 2024.
12. Morello RT, Barker AL, Watts JJ, et al. The extra resource burden of in-hospital falls: a cost of falls study. *Medical Journal of Australia* 2015;203(9):367. doi: https://doi.org/10.5694/mja15.00296
13. Registered Nurses' Association of Ontario. *Preventing Falls and Reducing Injury from Falls.* Toronto, ON: Registered Nurses' Association of Ontario, 2017. Accessed June 07, 2021. https://rnao.ca/sites/rnao-ca/files/bpg/FALL_PREVENTION_WEB_1207-17.pdf.
14. NDNQI - Nursing excellence made simple: PressGaney; 2023 [Available from: https://www.pressganey.com/platform/ndnqi/ accessed July 11, 2023.
15. Staggs VS, Davidson J, Dunton N, et al. Challenges in defining and categorizing falls on diverse unit types: lessons from expansion of the NDNQI Falls Indicator. *Journal of Nursing Care Quality* 2015;30(2):106-12. doi: https://doi.org/10.1097/ncq.0000000000000085
16. Halfens RJG, Meesterberends E, van Nie NC, et al. International prevalence measurement of care problems: results. *Journal of Advanced Nursing* 2013;69(9):e5-c17. doi: https://doi.org/10.1111/jan.12189
17. Van Nie NC, Schols JMGA, Meesterberends E, et al. An international prevalence measurement of care problems: study protocol. *Journal of Advanced Nursing* 2013;69(9):c18-c29. doi: https://doi.org/10.1111/jan.12190
18. Bernet NS, Everink IHJ, Schols JMGA, et al. Hospital performance comparison of inpatient fall rates; the impact of risk adjusting for patient-related factors: a multicentre cross-sectional survey. *BMC Health Services Research* 2022;22(1):225. doi: https://doi.org/10.1186/s12913-022-07638-7
19. Busch P. ANQ-Qualitätsmessungen in Spitälern und Kliniken – quo vadis? *saez* 2019;100(41):1350-52. doi: https://doi.org/10.4414/saez.2019.18271
20. Thomann S, Röösli R, Richter D, et al. *Auswertungskonzept ANQ: Nationale Prävalenzmessung Sturz und Dekubitus.* Bern: Nationaler Verein für Qualitätsentwicklung in Spitälern und Kliniken (ANQ) & Berner Fachhochschule (BFH), 2020. Accessed December 14, 2021. https://www.anq.ch/wp-content/uploads/2017/12/ANQ_Sturz_Dekubitus_Auswertungskonzept.pdf.
21. Thomann S, Zimmermann R, Riedweg JS, et al. National developments in falls and pressure ulcers in Swiss hospitals from 2011-2019: a secondary data analysis of the national quality measurement. *Manuscript submitted for publication* 2023,
22. World Health Organization (WHO). Regional Office for Europe. *Health and care workforce in Europe: time to act.* Copenhagen: World Health Organization. Regional

For peer review only



For peer review only - http://bmjopen.bmj.com/site/about/guidelines.xhtml

Office for Europe, 2022. Accessed February 23, 2024. https://apps.who.int/iris/handle/10665/362379.

23. Lane-Fall MB, Neuman MD. Outcomes measures and risk adjustment. *International Anesthesiology Clinics* 2013;51(4):10-21. doi: https://doi.org/10.1097/AIA.0b013e3182a70a52

24. Everink I, Gordon A, Rijcken S, et al. Learning and improving in long-term care internationally: the International Prevalence Measurement of Care Quality (LPZ). *Geriatrics, Gerontology and Aging* 2021;15(2021) doi: http://dx.doi.org/10.53886/gga.e0210056

25. Bours GJ, Halfens RJG, Lubbers M, et al. The development of a national registration form to measure the prevalence of pressure ulcers in the Netherlands. *Ostomy Wound Management* 1999;45(11):28-33, 36-8, 40.

26. Gorecki C, Brown J, Cano S, et al. Development and validation of a new patient-reported outcome measure for patients with pressure ulcers: the PU-QOL instrument. *Health and Quality of Life Outcomes* 2013;11(1):95. doi: https://doi.org/10.1186/1477-7525-11-95

27. Halfens RJG, Meesterberends E, Meijers JMM, et al. *Landelijke Prevalentiemeting Zorgproblemen. Rapportage resultaten 2011.* Maastricht: 2011. Accessed June 03, 2021. https://nl.lpz-um.eu/Content/Public/NL/Publications/LPZ%20Rapport%202011.pdf.

28. Kellogg International Work Group on the Prevention of Falls by the Elderly. The prevention of falls in later life. A report of the Kellogg International Work Group on the Prevention of Falls by the Elderly. *Danish medical bulletin* 1987;34(Supplement 4):1-24.

29. World Health Organization. International Statistical Classification of Diseases and Related Health Problems 10th Revision 2016 [Available from: https://icd.who.int/browse10/2016/en accessed January 19, 2023.

30. Dijkstra A, Smith J, White M. *Manual Care Dependency Scale. Measuring care dependency with the Care Dependency Scale (CDS). A manual.* 2006. Accessed December 02, 2019. https://www.care2share.eu/dbfiles/download/29.

31. Lohrmann C, Dijkstra A, Dassen T. The Care Dependency Scale: an assessment instrument for elderly patients in German hospitals. *Geriatric Nursing* 2003 doi: https://doi.org/10.1067/mgn.2003.8

32. Eglseer D, Halfens RJG, Schols JMGA, et al. Dysphagia in Hospitalized Older Patients: Associated Factors and Nutritional Interventions. *Journal of Nutrition, Health and Aging* 2018;22(1):103-10. doi: https://doi.org/10.1007/s12603-017-0928-x

33. Schüttengruber G, Halfens RJG, Lohrmann C. Care dependency of patients and residents at the end of life: A secondary data analysis of data from a cross-sectional study in hospitals and geriatric institutions. *Journal of Clinical Nursing* 2022;31(5-6):657-68. doi: https://doi.org/10.1111/jocn.15925

34. Armitage P. Tests for Linear Trends in Proportions and Frequencies. *Biometrics* 1955;11(3):375-86. doi: https://www.doi.org/10.2307/3001775

35. Akaike H. A new look at the statistical model identification. *IEEE Transactions on Automatic Control* 1974;19(6):716-23. doi: https://doi.org/10.1109/TAC.1974.1100705

36. Rabe-Hesketh S, Skrondal A. Multilevel and Longitudinal Modeling Using Stata, Volumes I and II. 4 ed. College Station, Texas: Stata Press 2022.

37. R: A Language and Environment for Statistical Computing [program]. Vienna, Austria: R Foundation for Statistical Computing, 2021.



For peer review only - http://bmjopen.bmj.com/site/about/guidelines.xhtml

38. DescTools: Tools for Descriptive Statistics [program]. R package version 0.99.48 version, 2023.

39. Venables WN, Ripley BD. Modern Applied Statistics with S. Fourth ed. New York: Springer 2002.

40. Bates D, Mächler M, Bolker B, et al. Fitting Linear Mixed-Effects Models Using lme4. *Journal of Statistical Software* 2015;67(1):1-48. doi: https://doi.org/10.18637/jss.v067.i01

41. Fox J. Effect Displays in {R} for Generalised Linear Models. *Journal of Statistical Software* 2003;8(15):1–27. doi: https://doi.org/10.18637/jss.v008.i15

42. Fox J, Weisberg S. An R Companion to Applied Regression. 3rd ed. Thousand Oaks CA: Sage 2019.

43. sjPlot: Data Visualization for Statistics in Social Science [program], 2021.

44. Bouldin ELD, Andresen EM, Dunton NE, et al. Falls among adult patients hospitalized in the United States: prevalence and trends. *Journal of Patient Safety* 2013;9(1):13-17. doi: https://doi.org/10.1097/PTS.0b013e3182699b64

45. He J, Dunton N, Staggs V. Unit-level Time Trends in Inpatient Fall Rates of US Hospitals. *Medical Care* 2012;50(9):801-07. doi: https://doi.org/10.1097/mlr.0b013e31825a8b88

46. Campanella P, Vukovic V, Parente P, et al. The impact of Public Reporting on clinical outcomes: a systematic review and meta-analysis. *BMC Health Services Research* 2016;16(1):296. doi: https://doi.org/10.1186/s12913-016-1543-y

47. Brown B, Gude WT, Blakeman T, et al. Clinical Performance Feedback Intervention Theory (CP-FIT): a new theory for designing, implementing, and evaluating feedback in health care based on a systematic review and meta-synthesis of qualitative research. *Implementation Science* 2019;14(1):40. doi: https://doi.org/10.1186/s13012-019-0883-5

48. Dowding D, Randell R, Gardner P, et al. Dashboards for improving patient care: Review of the literature. *International Journal of Medical Informatics* 2015;84(2):87-100. doi: https://doi.org/10.1016/j.ijmedinf.2014.10.001

49. Patientensicherheit Schweiz. *Sturzprävention.* Basel/Zürich: Stiftung für Patientensicherheit, 2008. Accessed February 18, 2024. https://patientensicherheit.ch/wp/wp-content/uploads/2023/03/2_SR_2_Sturzpra__vention_D_160725.pdf.

50. PHS Public Health Services. *StoppSturz / Parachutes (2019-2022) – Sturzprävention in der ambulanten Gesundheitsversorgung. Projekt-Schlussbericht der Gesamtprojektleitung.* Bern: PHS Public Health Services, 2023. Accessed February 18, 2024. https://www.bfu.ch/media/13unxwu3/230314-stoppsturz-schlussbericht_pa.pdf.

51. VanGilder CA, Cox J, Edsberg LE, et al. Pressure Injury Prevalence in Acute Care Hospitals With Unit-Specific Analysis: Results From the International Pressure Ulcer Prevalence (IPUP) Survey Database. *J Wound Ostomy Continence Nurs* 2021;48(6):492-503. doi: https://doi.org/10.1097/won.0000000000000817

52. Thomann S, Schlunegger M, Röösli R, et al. *Sturz und Dekubitus Akutsomatik Erwachsene - Nationaler Vergleichsbericht Messung 2019.* Bern: Nationaler Verein für Qualitätsentwicklung in Spitälern und Kliniken (ANQ) & Berner Fachhochschule (BFH), 2020. Accessed February 01, 2024. https://www.anq.ch/wp-content/uploads/2020/09/ANQakut_Sturz_Dekubitus_Erwachsene_Nationaler-Vergleichsbericht_2019.pdf.

53. Merçay C, Grünig A, Dolder P. *Gesundheitspersonal in der Schweiz – Nationaler Versorgungsbericht 2021. Bestand, Bedarf, Angebot und Massnahmen zur*




For peer review only - http://bmjopen.bmj.com/site/about/guidelines.xhtml

*Personalsicherung*. Neuchâtel: Schweizerisches Gesundheitsobservatorium (Obsan), 2021. Accessed February 23, 2024. https://www.obsan.admin.ch/sites/default/files/2021-10/Obsan_03_2021_BERICHT_0.pdf.

54. Dall'Ora C, Saville C, Rubbo B, et al. Nurse staffing levels and patient outcomes: A systematic review of longitudinal studies. *International Journal of Nursing Studies* 2022;134:104311. doi: https://doi.org/10.1016/j.ijnurstu.2022.104311
55. Kim J, Lee E, Jung Y, et al. Patient-level and organizational-level factors influencing in-hospital falls. *Journal of Advanced Nursing* 2022;78(11):3641-51. doi: https://doi.org/10.1111/jan.15254
56. Bundesamt für Gesundheit BAG. *Kennzahlen der Schweizer Spitäler 2021*. Bern: Bundesamt für Gesundheit (BAG), 2023. Accessed February 23, 2024. https://spitalstatistik.bagapps.ch/data/download/kzp21_publication.pdf?v=1679384362.
57. Agency for Healthcare Research and Quality. *Network of Patient Safety Databases Chartbook, 2022*. Rockville, MD: AHRQ Pub, 2022. Accessed February 23, 2024. https://www.ahrq.gov/sites/default/files/wysiwyg/npsd/data/npsd-chartbook-2022.pdf#FallSupp.
58. Morris ME, Webster K, Jones C, et al. Interventions to reduce falls in hospitals: a systematic review and meta-analysis. *Age Ageing* 2022;51(5) doi: https://doi.org/10.1093/ageing/afac077
59. Lan X, Li H, Wang Z, et al. Frailty as a predictor of future falls in hospitalized patients: A systematic review and meta-analysis. *Geriatric Nursing* 2020;41(2):69-74. doi: https://doi.org/10.1016/j.gerinurse.2019.01.004
60. Yeung SSY, Reijnierse EM, Pham VK, et al. Sarcopenia and its association with falls and fractures in older adults: A systematic review and meta-analysis. *Journal of Cachexia, Sarcopenia and Muscle* 2019;10(3):485-500. doi: https://doi.org/10.1002/jcsm.12411
61. Ishida Y, Maeda K, Nonogaki T, et al. Malnutrition at Admission Predicts In-Hospital Falls in Hospitalized Older Adults. *Nutrients* 2020;12(2) doi: https://doi.org/10.3390/nu12020541
62. Zhao YL, Kim H. Older Adult Inpatient Falls in Acute Care Hospitals: Intrinsic, Extrinsic, and Environmental Factors. *Journal of Gerontological Nursing* 2015;41(7):29-43; quiz 44-5. doi: https://doi.org/10.3928/00989134-20150616-05
63. Moon S, Chung HS, Kim YJ, et al. The impact of urinary incontinence on falls: A systematic review and meta-analysis. *PLOS ONE* 2021;16(5):e0251711. doi: https://doi.org/10.1371/journal.pone.0251711
64. Seppala LJ, van de Glind EMM, Daams JG, et al. Fall-Risk-Increasing Drugs: A Systematic Review and Meta-analysis: III. Others. *Journal of the American Medical Directors Association* 2018;19(4):372.e1-72.e8. doi: https://doi.org/10.1016/j.jamda.2017.12.099
65. de Vries M, Seppala LJ, Daams JG, et al. Fall-Risk-Increasing Drugs: A Systematic Review and Meta-Analysis: I. Cardiovascular Drugs. *Journal of the American Medical Directors Association* 2018;19(4):371.e1-71.e9. doi: https://doi.org/10.1016/j.jamda.2017.12.013
66. Seppala LJ, Wermelink AMAT, de Vries M, et al. Fall-Risk-Increasing Drugs: A Systematic Review and Meta-Analysis: II. Psychotropics. *Journal of the American Medical Directors Association* 2018;19(4):371.e11-71.e17. doi: https://doi.org/10.1016/j.jamda.2017.12.098



For peer review only - http://bmjopen.bmj.com/site/about/guidelines.xhtml

67. Bernet NS, Everink IH, Hahn S, et al. Comparing risk-adjusted fall rates internationally: Validation of a risk-adjustment model using multicentre cross-sectional data from hospitals in Switzerland and Austria. *Manuscript submitted for publication* 2023,
68. Bernet NS, Thomann S, Kurpicz-Briki M, et al. Potential of Electronic Medical Record Data for National Quality Measurement. *Studies in Health Technology and Informatics* 2022;292:51-56. doi: https://doi.org/10.3233/shti220320

For peer review only



For peer review only - http://bmjopen.bmj.com/site/about/guidelines.xhtml

*Figure 1: Visualisation of the descriptive Swiss national inpatient fall rates from 2011 to 2019 and the estimated linear trend over time*

*Figure 2: Visualisation of predicted risk-adjusted (without and with time trend) Swiss national inpatient fall rates from 2011 to 2019*

For peer review only



For peer review only - http://bmjopen.bmj.com/site/about/guidelines.xhtml

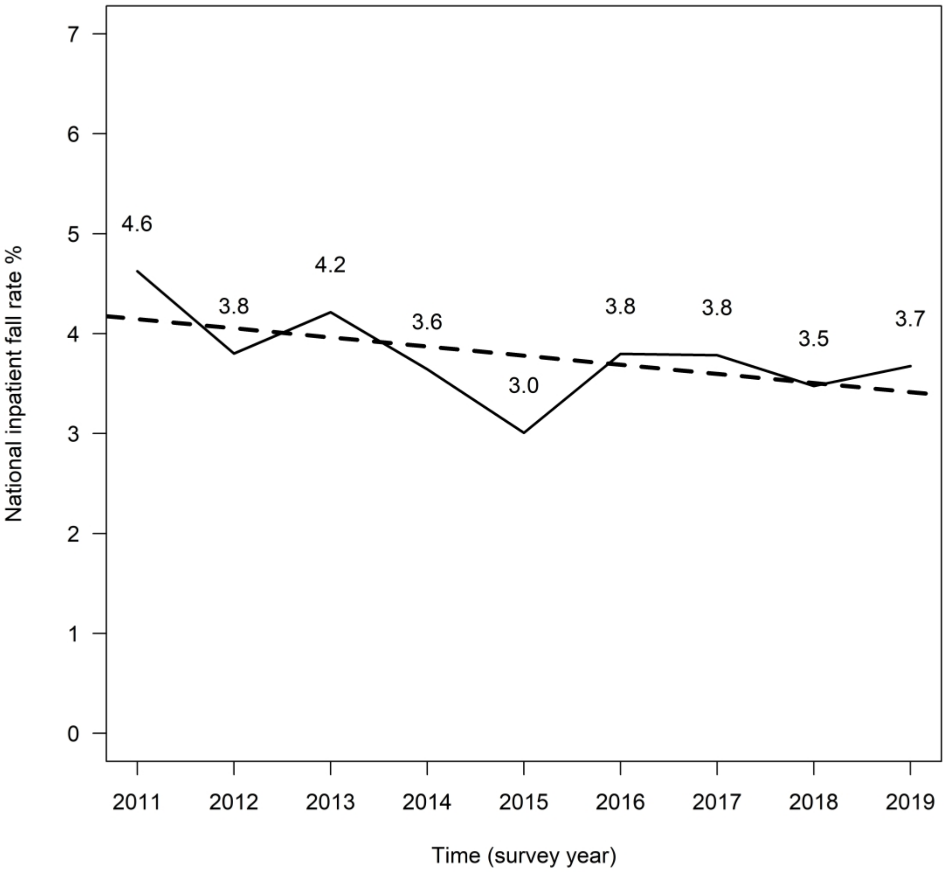


Figure 1: Visualisation of the descriptive Swiss national inpatient fall rates from 2011 to 2019 and the estimated linear trend over time.

482x482mm (315 x 315 DPI)

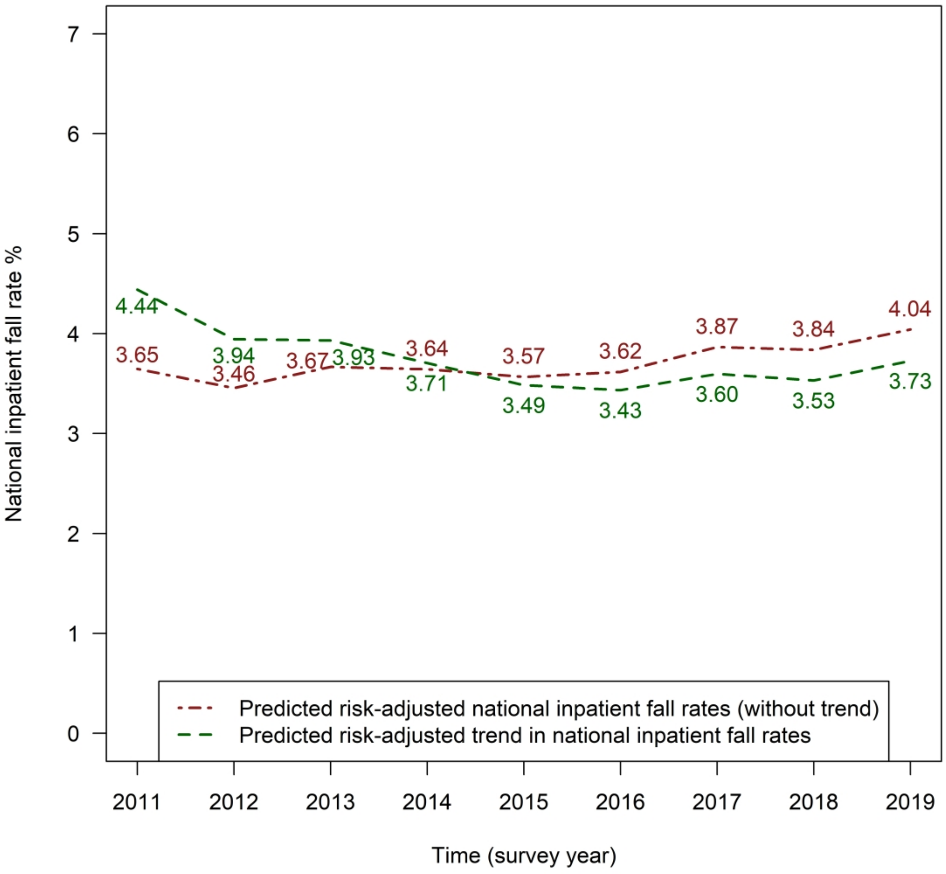


Figure 2: Visualisation of predicted risk-adjusted (without and with time trend) Swiss national inpatient fall rates from 2011 to 2019

482x482mm (300 x 300 DPI)

# Supplementary file

| BFH national analysis dataset | | 2011 | 2012 | 2013 | 2014 | 2015 | 2016 | 2017 | 2018 | 2019 |
|---|---|---|---|---|---|---|---|---|---|---|
| *Survey year* | | **2011** | **2012** | **2013** | **2014** | **2015** | **2016** | **2017** | **2018** | **2019** |
| | *n=* | 15637 | 18105 | 17486 | 17550 | 17233 | 17619 | 17438 | 17250 | 17464 |
| **Exclusion**: Non-comparable hospital/ward types | *n=* | 42 | - | - | - | - | - | - | - | - |
| Patients younger than 18 years | *n=* | 71 | - | - | - | - | - | - | - | - |
| Conflicting cases: Non-participation, but data available; participation, but missings on mandatory variables | *n=* | 44 | 5 | 3 | 1 | - | - | - | - | - |
| Unknown on outcome variable | *n=* | 172 | 203 | 209 | 167 | 158 | 11 | 41 | 78 | 71 |
| Non-participation | *n=* | 4939 | 4453 | 4580 | 4233 | 4070 | 4154 | 4211 | 4023 | 4224 |
| No data available on care dependency* | *n=* | 4726 | - | - | - | - | - | - | - | - |
| **Study population** | *n=* | 5643 | 13444 | 12694 | 13149 | 13005 | 13454 | 13186 | 13149 | 13169 |
| | *%* | 36.1% | 74.3% | 72.6% | 74.9% | 75.5% | 76.4% | 75.6% | 76.2% | 75.4% |
| **Merged analysis dataset** | *n=* | | | | | 110893 | | | | |
| | *%* | | | | | 71.2% | | | | |

*In 2011, the hospitals w ere free to decide w hether to collect the Care Dependency Scale (CDS). In the analyses, 4726 patients from the survey year 2011 therefore had to be excluded due to missing values. No significant group difference w as found in the frequency of inpatient falls in the excluded group (no information on CDS available) compared to the included group (information on CDS available) (Chi-Square *p* =0.107).

*Supplementary figure S1: Flowchart depicting the selection of cases for analysis*



For peer review only - http://bmjopen.bmj.com/site/about/guidelines.xhtml

*Supplementary table S1: Description of patient characteristics in the sample across the survey years*

| | **2011** *n* = 5643 | **2012** *n* = 13444 | **2013** *n* = 12694 | **2014** *n* = 13149 | **2015** *n* = 13005 | **2016** *n* = 13454 | **2017** *n* = 13186 | **2018** *n* = 13149 | **2019** *n* = 13169 | **Total** *n* = 110893 |
|---|---|---|---|---|---|---|---|---|---|---|
| **Patient characteristics** | *n* (%) | *n* (%) | *n* (%) | *n* (%) | *n* (%) | *n* (%) | *n* (%) | *n* (%) | *n* (%) | *n* (%) |
| Sex [female] | 3000 (53.2) | 7301 (54.3) | 6400 (50.4) | 6693 (50.9) | 6519 (50.1) | 6646 (49.4) | 6527 (49.5) | 6458 (49.1) | 6471 (49.1) | 56015 (50.5) |
| Care dependency (CDS) | | | | | | | | | | |
| care independent [70-75] | 3106 (55.0) | 7534 (56.0) | 6790 (53.5) | 7182 (54.6) | 7080 (54.4) | 7340 (54.6) | 7036 (53.4) | 7000 (53.2) | 6807 (51.7) | 59875 (54.0) |
| to a great extent independent [60-69] | 1288 (22.8) | 2893 (21.5) | 2963 (23.3) | 2989 (22.7) | 3072 (23.6) | 3236 (24.1) | 3130 (23.7) | 3174 (24.1) | 3225 (24.5) | 25970 (23.4) |
| partially dependent [45-59] | 707 (12.5) | 1844 (13.7) | 1813 (14.3) | 1879 (14.3) | 1767 (13.6) | 1760 (13.1) | 1890 (14.3) | 1888 (14.4) | 1960 (14.9) | 15508 (14.0) |
| to a great extent dependent [25-44] | 408 (7.2) | 883 (6.6) | 850 (6.7) | 814 (6.2) | 783 (6.0) | 776 (5.8) | 817 (6.2) | 792 (6.0) | 869 (6.6) | 6992 (6.3) |
| completely dependent [15-24] | 134 (2.4) | 290 (2.2) | 278 (2.2) | 285 (2.2) | 303 (2.3) | 342 (2.5) | 313 (2.4) | 295 (2.2) | 308 (2.3) | 2548 (2.3) |
| Surgical procedure within 14 days prior to measurement [yes] | 2111 (37.4) | 5336 (39.7) | 5218 (41.1) | 5469 (41.6) | 5708 (43.9) | 5981 (44.5) | 5863 (44.5) | 5774 (43.9) | 5773 (43.8) | 47233 (42.6) |
| ICD-10 – Diseases of the circulatory system [yes] | 2949 (52.3) | 6608 (49.2) | 6453 (50.8) | 6622 (50.4) | 6755 (51.9) | 7275 (54.1) | 7368 (55.9) | 7397 (56.3) | 7560 (57.4) | 58987 (53.2) |
| ICD-10 – Diseases of the musculoskeletal system and connective tissue [yes] | 1876 (33.2) | 4317 (32.1) | 4632 (36.5) | 4919 (37.4) | 5041 (38.8) | 5550 (41.3) | 5373 (40.7) | 5357 (40.7) | 5284 (40.1) | 42349 (38.2) |
| ICD-10 – Endocrine, nutritional and metabolic diseases [yes] | 1476 (26.2) | 3277 (24.4) | 3569 (28.1) | 3788 (28.8) | 4047 (31.1) | 4359 (32.4) | 4522 (34.3) | 4430 (33.7) | 4776 (36.3) | 34244 (30.9) |
| ICD-10 – Diseases of the genitourinary system [yes] | 1578 (28.0) | 3519 (26.2) | 3387 (26.7) | 3522 (26.8) | 3509 (27.0) | 4086 (30.4) | 4085 (31.0) | 4028 (30.6) | 4365 (33.1) | 32079 (28.9) |
| ICD-10 – Diseases of the digestive system [yes] | 1538 (27.3) | 3191 (23.7) | 2991 (23.6) | 3155 (24.0) | 3096 (23.8) | 3417 (25.4) | 3443 (26.1) | 3385 (25.7) | 3668 (27.9) | 27884 (25.1) |
| ICD-10 – Diseases of the respiratory system [yes] | 1175 (20.8) | 2592 (19.3) | 2805 (22.1) | 2837 (21.6) | 3038 (23.4) | 3259 (24.2) | 3241 (24.6) | 3181 (24.2) | 3472 (26.4) | 25600 (23.1) |
| ICD-10 – Neoplasms [yes] | 1014 (18.0) | 2204 (16.4) | 1739 (13.7) | 2426 (18.5) | 2450 (18.8) | 2716 (20.2) | 2836 (21.5) | 2792 (21.2) | 2990 (22.7) | 21167 (19.1) |
| ICD-10 – Mental, behavioural and neurodevelopmental disorders [yes] | 878 (15.6) | 2019 (15.0) | 2222 (17.5) | 2196 (16.7) | 2182 (16.8) | 2485 (18.5) | 2609 (19.8) | 2531 (19.2) | 2705 (20.5) | 19827 (17.9) |
| ICD-10 – Diseases of the blood and blood-forming organs [yes] | 494 (8.8) | 1089 (8.1) | 1663 (13.1) | 1739 (13.2) | 1785 (13.7) | 2083 (15.5) | 2145 (16.3) | 2092 (15.9) | 2371 (18.0) | 15461 (13.9) |
| ICD-10 – Diseases of the nervous system [yes] | 465 (8.2) | 1139 (8.5) | 1333 (10.5) | 1598 (12.2) | 1496 (11.5) | 1813 (13.5) | 1897 (14.4) | 1823 (13.9) | 1930 (14.7) | 13494 (12.2) |
| ICD-10 – Certain infectious and parasitic diseases [yes] | 752 (13.3) | 1672 (12.4) | 1325 (10.4) | 1365 (10.4) | 1386 (10.7) | 1695 (12.6) | 1823 (13.8) | 1663 (12.6) | 1813 (13.8) | 13494 (12.2) |
| ICD-10 – Diseases of the eye and adnexa OR ICD-10 – Diseases of the ear and mastoid process [yes] | 390 (6.9) | 745 (5.5) | 871 (6.9) | 904 (6.9) | 919 (7.1) | 1046 (7.8) | 1091 (8.3) | 1046 (8.0) | 1136 (8.6) | 8148 (7.3) |
| ICD-10 – Diseases of the skin and subcutaneous tissue [yes] | 272 (4.8) | 603 (4.5) | 812 (6.4) | 848 (6.4) | 909 (7.0) | 988 (7.3) | 1031 (7.8) | 1021 (7.8) | 1088 (8.3) | 7572 (6.8) |
| ICD-10 – Injury, poisoning and certain other consequences of external causes [yes] | 631 (11.2) | 1207 (9.0) | 922 (7.3) | 802 (6.1) | 762 (5.9) | 808 (6.0) | 708 (5.4) | 875 (6.7) | 844 (6.4) | 7559 (6.8) |
| ICD-10 – Congenital malformations, deformations and chromosomal abnormalities [yes] | 34 (0.6) | 72 (0.5) | 82 (0.6) | 67 (0.5) | 77 (0.6) | 70 (0.5) | 64 (0.5) | 81 (0.6) | 78 (0.6) | 625 (0.6) |
| | M (IQR) | M (IQR) | M (IQR) | M (IQR) | M (IQR) | M (IQR) | M (IQR) | M (IQR) | M (IQR) | M (IQR) |
| Age [in years] | 69 (25) | 68 (27) | 69 (24) | 69 (24) | 69 (23) | 70 (24) | 70 (24) | 70 (23) | 71 (23) | 70 (24) |

M=Median; IQR=interquartile range.



For peer review only - http://bmjopen.bmj.com/site/about/guidelines.xhtml

For peer review only

*Supplementary table S2: Illustration of the estimated non-linear trend using odds ratios, each of which describes the decrease in Swiss national fall rates in relation to the previous survey year*

| **Survey year** | **2011/2012** | **2012/2013** | **2013/2014** | **2014/2015** | **2015/2016** | **2016/2017** | **2017/2018** | **2018/2019** |
|---|---|---|---|---|---|---|---|---|
| *OR* | 0.92 | 0.93 | 0.94 | 0.95 | 0.97 | 0.98 | 0.99 | 1.00 |
| 95% *CI* | 0.88 – 0.96 | 0.90 – 0.96 | 0.92 – 0.96 | 0.94 – 0.97 | 0.95 – 0.98 | 0.96 – 1.00 | 0.96 – 1.02 | 0.96 – 1.04 |

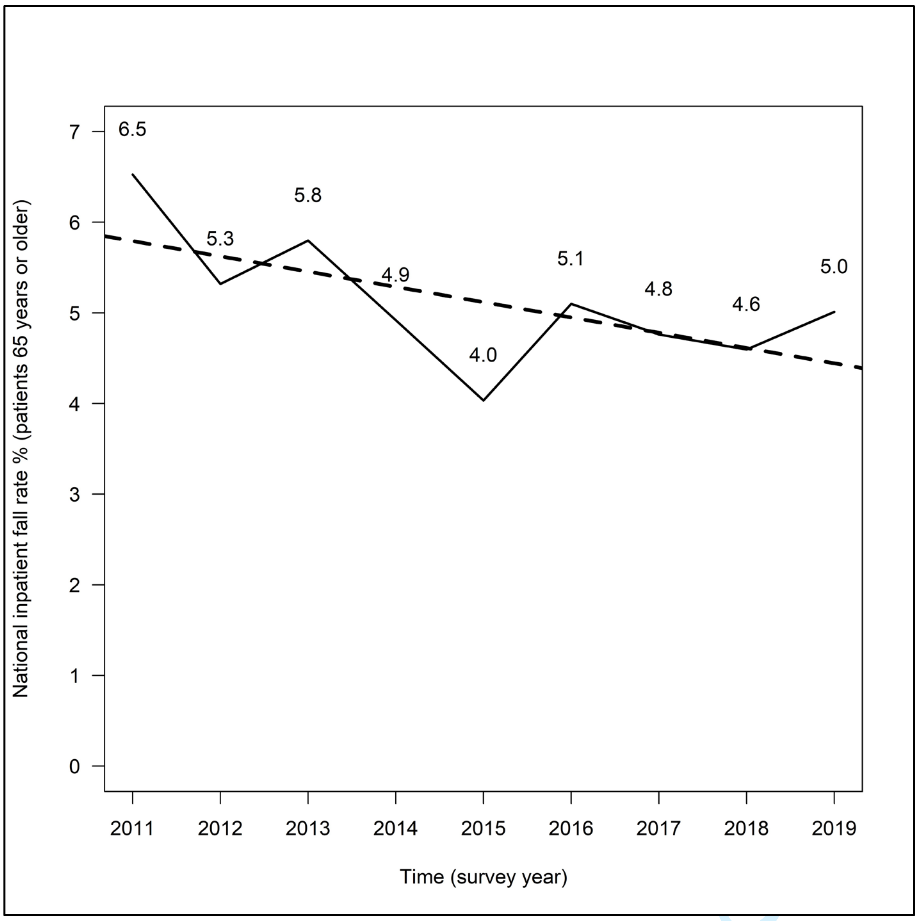


*Supplementary figure S2: Visualisation of the descriptive Swiss national inpatient fall rates from 2011 to 2019 and the estimated linear trend over time, taking into account only patients 65 years or older*



For peer review only - http://bmjopen.bmj.com/site/about/guidelines.xhtml

*Supplementary table S3: Overview of the two-level random intercept logistic regression model used to derive the risk-adjusted trend in Swiss national inpatient fall rates, taking into account only patients 65 years or older*

| | **Risk-adjusted trend in national inpatient fall rates** | | | | |
|---|---|---|---|---|---|
| *Predictors* | *coeff.* | *SE* | *p-value* | *OR* | *95% CI* |
| (Intercept) | -4.52 | 0.21 | **<0.001** | - | - |
| **Time related factors (trend)** | | | | | |
| Time | -0.12 | 0.04 | **0.001** | - | - |
| (Time)^2 | 0.01 | 0.00 | **0.020** | - | - |
| **Patient related fall risk factors** | | | | | |
| Age [in years] | 0.01 | 0.00 | **<0.001** | 1.01 | 1.01 – 1.02 |
| CDS [care independent (70-75)] | Ref. | | | | |
| CDS [to a great extent independent (60-69)] | 0.97 | 0.06 | **<0.001** | 2.64 | 2.36 – 2.95 |
| CDS [partially dependent (45-59)] | 1.38 | 0.06 | **<0.001** | 3.96 | 3.54 – 4.43 |
| CDS [to a great extent dependent (25-44)] | 1.66 | 0.06 | **<0.001** | 5.25 | 4.63 – 5.94 |
| CDS [completely dependent (15-24)] | 1.30 | 0.09 | **<0.001** | 3.68 | 3.05 – 4.43 |
| ICD-10 – Mental and Behavioural disorders [yes] | 0.53 | 0.04 | **<0.001** | 1.71 | 1.58 – 1.85 |
| ICD-10 – Neoplasms [yes] | 0.32 | 0.04 | **<0.001** | 1.38 | 1.27 – 1.50 |
| ICD-10 – Diseases of the nervous system [yes] | 0.21 | 0.05 | **<0.001** | 1.24 | 1.13 – 1.36 |
| ICD-10 – Diseases of the blood and blood-forming organs [yes] | 0.21 | 0.04 | **<0.001** | 1.24 | 1.13 – 1.35 |
| ICD-10 – Injury, poisoning, other consequences of external causes [yes] | 0.18 | 0.06 | **0.003** | 1.20 | 1.06 – 1.36 |
| ICD-10 – Endocrine, nutritional and metabolic diseases [yes] | 0.09 | 0.04 | **0.015** | 1.09 | 1.02 – 1.18 |
| Sex [female] | -0.27 | 0.04 | **<0.001** | 0.76 | 0.71 – 0.82 |
| Surgical procedure within 14 days prior to measurement [yes] | -0.40 | 0.04 | **<0.001** | 0.67 | 0.62 – 0.73 |
| **Random Effects** | | | | | |
| $\tau_{00}$ [variability in hospital intercepts] | | | 0.09 | | |
| N [hospitals] | | | 221 | | |
| Observations | | | 67336 | | |

Significant *p*-values are highlighted in bold.



For peer review only - http://bmjopen.bmj.com/site/about/guidelines.xhtml

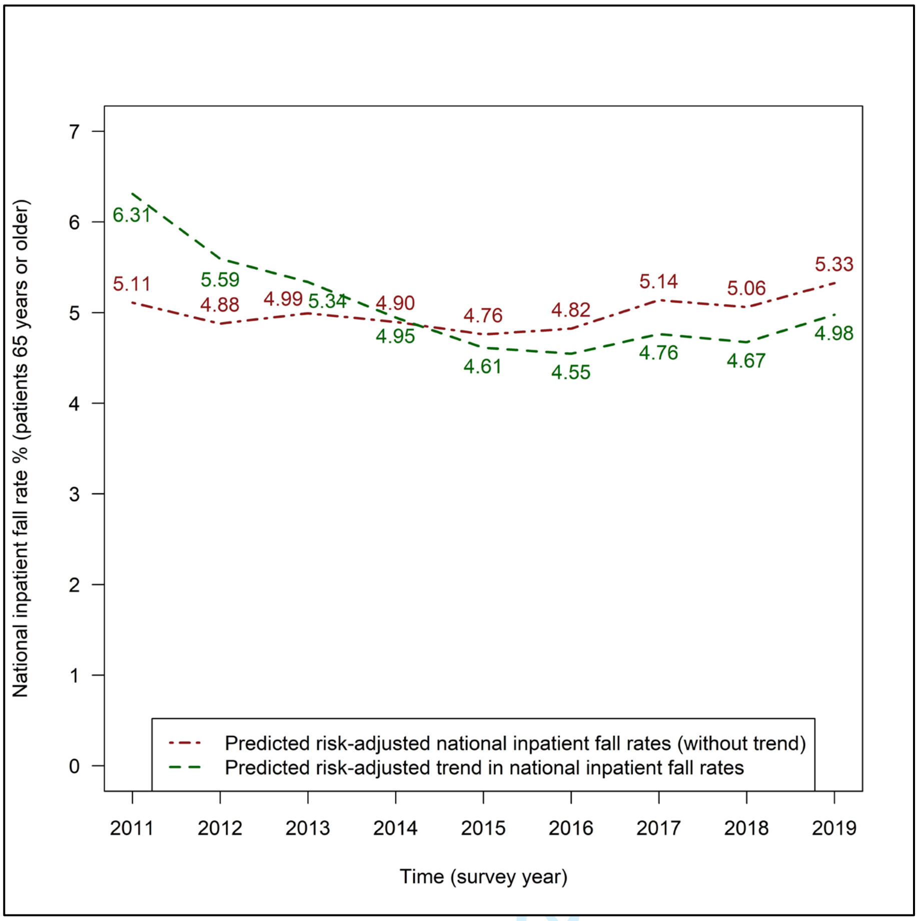


*Supplementary figure S3: Visualisation of predicted risk-adjusted (without and with time trend) Swiss national inpatient fall rates from 2011 to 2019, taking into account only patients 65 years or older*

*Supplementary table S4: Illustration of the estimated non-linear trend using odds ratios, each of which describes the decrease in Swiss national fall rates in relation to the previous survey year, taking into account only patients 65 years or older*

| **Survey year** | **2011/2012** | **2012/2013** | **2013/2014** | **2014/2015** | **2015/2016** | **2016/2017** | **2017/2018** | **2018/2019** |
|---|---|---|---|---|---|---|---|---|
| ***OR*** | 0.91 | 0.92 | 0.94 | 0.95 | 0.97 | 0.98 | 1.00 | 1.01 |
| ***95% CI*** | 0.86 – 0.96 | 0.89 – 0.96 | 0.91 – 0.96 | 0.93 – 0.97 | 0.95 – 0.98 | 0.96 – 1.00 | 0.96 – 1.03 | 0.97 – 1.06 |



For peer review only - http://bmjopen.bmj.com/site/about/guidelines.xhtml

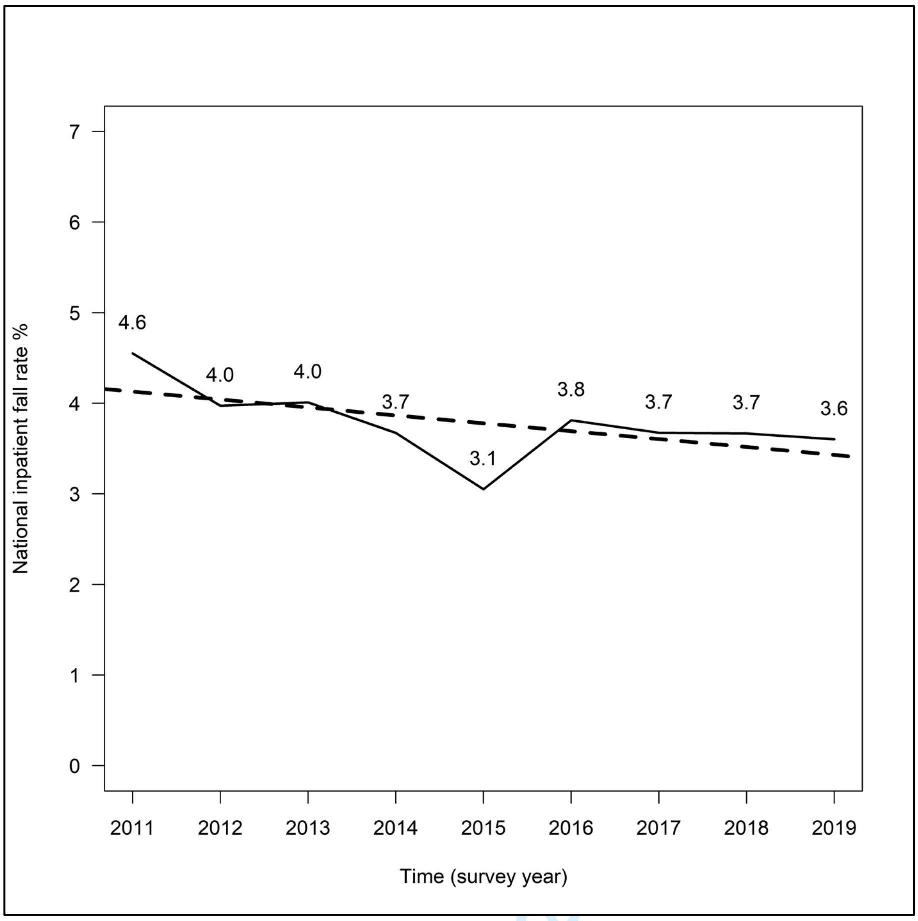


*Supplementary figure S4: Visualisation of the descriptive Swiss national inpatient fall rates from 2011 to 2019 and the estimated linear trend over time, taking into account only hospitals that provided data in all survey years*

For peer review only

*Supplementary table S5: Overview of the two-level random intercept logistic regression model used to derive the risk-adjusted trend in Swiss national inpatient fall rates, taking into account only hospitals that provided data in all survey years*

| | **Risk-adjusted trend in national inpatient fall rates** | | | | |
|---|---|---|---|---|---|
| *Predictors* | *coeff.* | *SE* | *p-value* | *OR* | *95% CI* |
| (Intercept) | -5.16 | 0.13 | **<0.001** | - | - |
| **Time related factors (trend)** | | | | | |
| Time | -0.05 | 0.01 | **<0.001** | - | - |
| **Patient related fall risk factors** | | | | | |
| Age [in years] | 0.01 | 0.00 | **<0.001** | 1.02 | 1.01 – 1.02 |
| CDS [care independent (70-75)] | Ref. | | | | |
| CDS [to a great extent independent (60-69)] | 1.04 | 0.06 | **<0.001** | 2.82 | 2.49 – 3.20 |
| CDS [partially dependent (45-59)] | 1.45 | 0.07 | **<0.001** | 4.25 | 3.73 – 4.84 |
| CDS [to a great extent dependent (25-44)] | 1.75 | 0.07 | **<0.001** | 5.76 | 4.99 – 6.66 |
| CDS [completely dependent (15-24)] | 1.28 | 0.11 | **<0.001** | 3.59 | 2.88 – 4.46 |
| ICD-10 – Mental and Behavioural disorders [yes] | 0.58 | 0.05 | **<0.001** | 1.79 | 1.64 – 1.96 |
| ICD-10 – Neoplasms [yes] | 0.35 | 0.05 | **<0.001** | 1.42 | 1.29 – 1.56 |
| ICD-10 – Diseases of the nervous system [yes] | 0.26 | 0.06 | **<0.001** | 1.30 | 1.16 – 1.45 |
| ICD-10 – Diseases of the blood and blood-forming organs [yes] | 0.22 | 0.05 | **<0.001** | 1.24 | 1.12 – 1.38 |
| ICD-10 – Endocrine, nutritional and metabolic diseases [yes] | 0.11 | 0.04 | **0.011** | 1.12 | 1.03 – 1.22 |
| ICD-10 – Diseases of the circulatory system [yes] | 0.12 | 0.05 | **0.021** | 1.12 | 1.02 – 1.24 |
| Sex [female] | -0.17 | 0.04 | **<0.001** | 0.85 | 0.78 – 0.92 |
| Surgical procedure within 14 days prior to measurement [yes] | -0.50 | 0.05 | **<0.001** | 0.60 | 0.55 – 0.67 |
| **Random Effects** | | | | | |
| $\tau_{00}$ [variability in hospital intercepts] | | | 0.06 | | |
| N [hospitals] | | | 93 | | |
| Observations | | | 64038 | | |

Significant *p*-values are highlighted in bold.



For peer review only - http://bmjopen.bmj.com/site/about/guidelines.xhtml

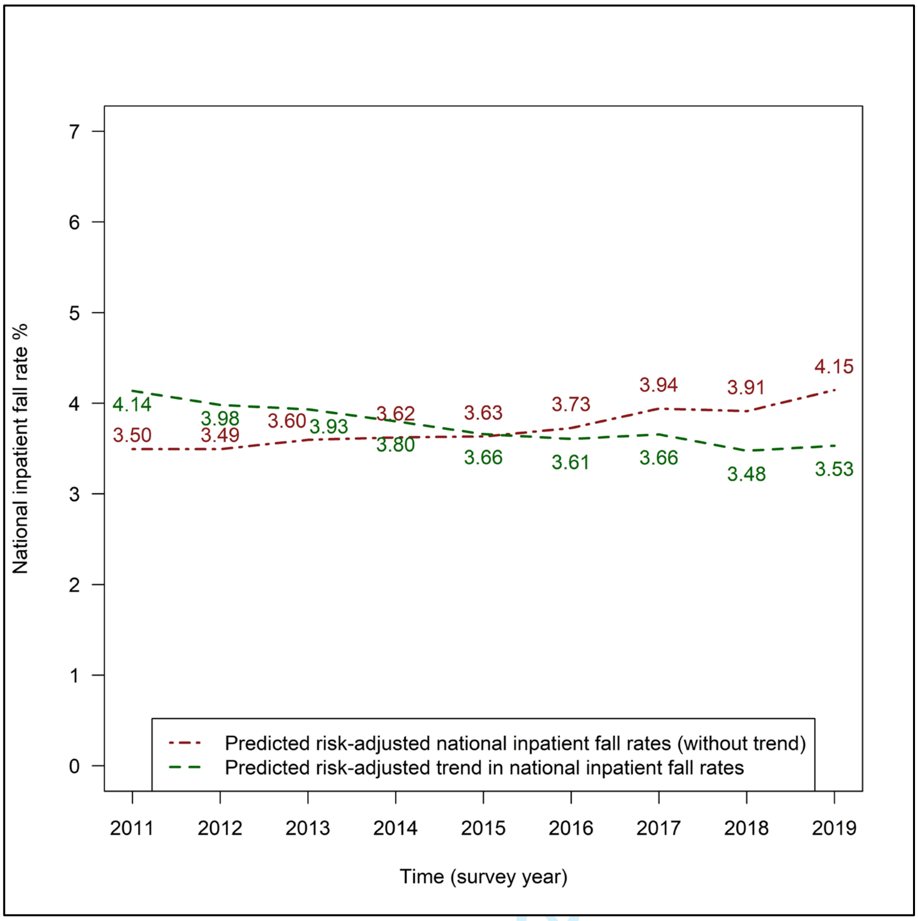


*Supplementary figure S5: Visualisation of predicted risk-adjusted (without and with time trend) Swiss national inpatient fall rates from 2011 to 2019, taking into account only hospitals that provided complete data in all survey years*



For peer review only - http://bmjopen.bmj.com/site/about/guidelines.xhtml

**STROBE 2007 (v4) Statement—Checklist of items that should be included in reports of *cross-sectional studies***

| **Section/Topic** | **Item #** | **Recommendation** | **Reported on page #** |
|---|---|---|---|
| **Title and abstract** | 1 | (*a*) Indicate the study's design with a commonly used term in the title or the abstract | 1 |
| | | (*b*) Provide in the abstract an informative and balanced summary of what was done and what was found | 2 |
| **Introduction** | | | |
| Background/rationale | 2 | Explain the scientific background and rationale for the investigation being reported | 5-7 |
| Objectives | 3 | State specific objectives, including any prespecified hypotheses | 7 |
| **Methods** | | | |
| Study design | 4 | Present key elements of study design early in the paper | 7 |
| Setting | 5 | Describe the setting, locations, and relevant dates, including periods of recruitment, exposure, follow-up, and data collection | 7-9 |
| Participants | 6 | (*a*) Give the eligibility criteria, and the sources and methods of selection of participants | 8-9 |
| Variables | 7 | Clearly define all outcomes, exposures, predictors, potential confounders, and effect modifiers. Give diagnostic criteria, if applicable | 9 |
| Data sources/ measurement | 8* | For each variable of interest, give sources of data and details of methods of assessment (measurement). Describe comparability of assessment methods if there is more than one group | 9 |
| Bias | 9 | Describe any efforts to address potential sources of bias | 8 |
| Study size | 10 | Explain how the study size was arrived at | 13 |
| Quantitative variables | 11 | Explain how quantitative variables were handled in the analyses. If applicable, describe which groupings were chosen and why | 10, 11 |
| Statistical methods | 12 | (*a*) Describe all statistical methods, including those used to control for confounding | 10-13 |
| | | (*b*) Describe any methods used to examine subgroups and interactions | 12 |
| | | (*c*) Explain how missing data were addressed | 13 |
| | | (*d*) If applicable, describe analytical methods taking account of sampling strategy | - |
| | | (*e*) Describe any sensitivity analyses | 12, 15 |
| **Results** | | | |

For peer review only

| | | | |
|---|---|---|---|
| Participants | 13* | (a) Report numbers of individuals at each stage of study—eg numbers potentially eligible, examined for eligibility, confirmed eligible, included in the study, completing follow-up, and analysed | 13 |
| | | (b) Give reasons for non-participation at each stage | 13 |
| | | (c) Consider use of a flow diagram | - |
| Descriptive data | 14* | (a) Give characteristics of study participants (eg demographic, clinical, social) and information on exposures and potential confounders | 13-14 |
| | | (b) Indicate number of participants with missing data for each variable of interest | 13 |
| Outcome data | 15* | Report numbers of outcome events or summary measures | 14-15 |
| Main results | 16 | (*a*) Give unadjusted estimates and, if applicable, confounder-adjusted estimates and their precision (eg, 95% confidence interval). Make clear which confounders were adjusted for and why they were included | 14-18 |
| | | (*b*) Report category boundaries when continuous variables were categorized | - |
| | | (*c*) If relevant, consider translating estimates of relative risk into absolute risk for a meaningful time period | - |
| Other analyses | 17 | Report other analyses done—eg analyses of subgroups and interactions, and sensitivity analyses | 18-19 |
| **Discussion** | | | |
| Key results | 18 | Summarise key results with reference to study objectives | 19-20 |
| Limitations | 19 | Discuss limitations of the study, taking into account sources of potential bias or imprecision. Discuss both direction and magnitude of any potential bias | 22 |
| Interpretation | 20 | Give a cautious overall interpretation of results considering objectives, limitations, multiplicity of analyses, results from similar studies, and other relevant evidence | 20-22 |
| Generalisability | 21 | Discuss the generalisability (external validity) of the study results | 22 |
| **Other information** | | | |
| Funding | 22 | Give the source of funding and the role of the funders for the present study and, if applicable, for the original study on which the present article is based | 32 |

*Give information separately for cases and controls in case-control studies and, if applicable, for exposed and unexposed groups in cohort and cross-sectional studies.

**Note:** An Explanation and Elaboration article discusses each checklist item and gives methodological background and published examples of transparent reporting. The STROBE checklist is best used in conjunction with this article (freely available on the Web sites of PLoS Medicine at http://www.plosmedicine.org/, Annals of Internal Medicine at http://www.annals.org/, and Epidemiology at http://www.epidem.com/). Information on the STROBE Initiative is available at www.strobe-statement.org.

For peer review only

# Risk-adjusted trend in national inpatient fall rates observed from 2011 to 2019 in acute care hospitals in Switzerland: a repeated multicentre cross-sectional study

| | |
|---|---|
| Journal: | *BMJ Open* |
| Manuscript ID | bmjopen-2023-082417.R2 |
| Article Type: | Original research |
| Date Submitted by the Author: | 22-Mar-2024 |
| Complete List of Authors: | Bernet, Niklaus; Bern University of Applied Sciences, School of Health Professions<br>Everink, Irma; Maastricht University Care and Public Health Research Institute, Department of Health Services Research<br>Hahn, Sabine; Bern University of Applied Sciences, School of Health Professions<br>Müller, Marianne; Bern University of Applied Sciences, School of Health Professions<br>Schols, Jos; Maastricht University Care and Public Health Research Institute, Department of Health Services Research |
| <b>Primary Subject Heading</b>: | Health services research |
| Secondary Subject Heading: | Nursing, Health policy, Health services research |
| Keywords: | Hospitals, Nursing Care, Quality in health care < HEALTH SERVICES ADMINISTRATION & MANAGEMENT, Risk Factors |
| | |

SCHOLARONE™
Manuscripts

*I, the Submitting Author has the right to grant and does grant on behalf of all authors of the Work (as defined in the below author licence), an exclusive licence and/or a non-exclusive licence for contributions from authors who are: i) UK Crown employees; ii) where BMJ has agreed a CC-BY licence shall apply, and/or iii) in accordance with the terms applicable for US Federal Government officers or employees acting as part of their official duties; on a worldwide, perpetual, irrevocable, royalty-free basis to BMJ Publishing Group Ltd ("BMJ") its licensees and where the relevant Journal is co-owned by BMJ to the co-owners of the Journal, to publish the Work in this journal and any other BMJ products and to exploit all rights, as set out in our licence.*

*The Submitting Author accepts and understands that any supply made under these terms is made by BMJ to the Submitting Author unless you are acting as an employee on behalf of your employer or a postgraduate student of an affiliated institution which is paying any applicable article publishing charge ("APC") for Open Access articles. Where the Submitting Author wishes to make the Work available on an Open Access basis (and intends to pay the relevant APC), the terms of reuse of such Open Access shall be governed by a Creative Commons licence – details of these licences and which Creative Commons licence will apply to this Work are set out in our licence referred to above.*

*Other than as permitted in any relevant BMJ Author's Self Archiving Policies, I confirm this Work has not been accepted for publication elsewhere, is not being considered for publication elsewhere and does not duplicate material already published. I confirm all authors consent to publication of this Work and authorise the granting of this licence.*

For peer review only

# Risk-adjusted trend in national inpatient fall rates observed from 2011 to 2019 in acute care hospitals in Switzerland: a repeated multicentre cross-sectional study

**Author names:**

Niklaus S Bernet[a], Irma HJ Everink[b], Sabine Hahn[a], Marianne Müller[a], Jos MGA Schols[b]

**Affiliations**:

[a] Bern University of Applied Sciences, School of Health Professions, Applied Research & Development in Nursing, Murtenstrasse 10, 3008 Bern, Switzerland

niklausstefan.bernet@bfh.ch, sabine.hahn@bfh.ch, marianne.mueller@bfh.ch

[b] Maastricht University, Care and Public Health Research Institute, Department of Health Services Research, PO BOX 616, 6200 MD, Maastricht, the Netherlands

i.everink@maastrichtuniversity.nl, jos.schols@maastrichtuniversity.nl

**Correspondence to:**

Niklaus S Bernet, Bern University of Applied Sciences, School of Health Professions, Applied Research & Development in Nursing, Murtenstrasse 10, 3008 Bern, Switzerland, +41318484585, niklausstefan.bernet@bfh.ch

Word count of the main manuscript (introduction, methods, results, discussion, conclusions): 4488



For peer review only - http://bmjopen.bmj.com/site/about/guidelines.xhtml

For peer review only

# Abstract

***Objectives:*** This study aimed to investigate whether a significant trend regarding inpatient falls in Swiss acute hospitals between 2011 and 2019 could be confirmed on a national level, and whether the trend persists after risk adjustment for patient-related fall risk factors.

***Design:*** A secondary data analysis was conducted based on annual multicentre cross-sectional studies carried out between 2011 and 2019.

***Setting:*** All Swiss acute care hospitals were obliged to participate in the surveys. Except for emergency departments, outpatient wards and recovery rooms, all wards were included.

***Participants:*** All inpatients aged 18 or older who had given their informed consent and whose data were complete and available were included.

***Outcome measure:*** Whether a patient had fallen in hospital was retrospectively determined on the survey day by asking patients the following question: Have you fallen in this institution in the last 30 days?

***Results:*** Based on data from 110892 patients from 222 Swiss hospitals, a national inpatient fall rate of 3.7% was determined over the nine survey years. A significant linear decreasing trend ($p$ = 0.004) was observed using the Cochran-Armitage trend test. After adjusting for patient-related fall risk factors in a two-level random intercept logistic regression model, a significant non-linear decreasing trend was found at the national level.

***Conclusions:*** A significant decrease in fall rates in Swiss hospitals, indicating an improvement in the quality of care provided, could be confirmed both descriptively




For peer review only - http://bmjopen.bmj.com/site/about/guidelines.xhtml

and after risk adjustment. However, the non-linear trend, i.e. an initial decrease in inpatient falls that flattens out over time, also indicates a possible future increase in fall rates. Monitoring of falls in hospitals should be maintained at national level. Risk adjustment accounts for the observed increase in patient-related fall risk factors in hospitals, thus promoting a fairer comparison of the quality of care provided over time.

**Strengths and limitations of this study**

- The internationally standardised primary data collection used in this study ensures high data quality in terms of completeness and uniformity, offering relevant advantages compared to the use of data generated for other purposes (e.g. a critical incidence reporting system).
- A comprehensive, nationally representative sample was used, comprising almost all Swiss hospitals with a somatic acute care mandate.
- The risk adjustment method used enables a fairer comparison of inpatient fall rates over time, as it controls for changes in patient-related fall risk factors.
- The cross-sectional survey design hinders consideration of the temporal sequence between inpatient fall risk factors and fall events, thereby limiting the recommended exclusive focus on pre-existing fall risk factors that cannot be influenced by hospital care in the risk adjustment model.

## Keywords

- Accidental falls
- Cross-Sectional Studies



For peer review only - http://bmjopen.bmj.com/site/about/guidelines.xhtml

- Hospitals
- Inpatients
- Quality Assurance
- Quality of Health Care
- Multilevel Analysis
- Risk Adjustment
- Trends

For peer review only



For peer review only - http://bmjopen.bmj.com/site/about/guidelines.xhtml

# INTRODUCTION

Regular national quality measurement in healthcare – the process of systematically measuring and reporting quality indicators at a national level to inform stakeholders at different levels of the health system – is assumed to drive quality improvement processes. This happens via two main pathways, as outlined by Berwick, et al. [1]. First, the "improvement by choice" pathway allows healthcare consumers to select providers based on publicly reported performance data. This incentivises providers to improve the quality of care due to market competition.[1 2] Second, the "improvement through change" pathway uses performance data to highlight areas where quality improvements are needed, fostering intrinsic motivation for providers to initiate improvements.[1 2] In addition, the fear of reputational damage from making performance data publicly available can be a further incentive to improve the quality of healthcare services.[3 4]

National quality measurement is therefore a recognised strategy for monitoring and improving the quality of healthcare by national policy-makers.[3 5] Regular quality measurement provides the basis for initiating data-driven quality improvement strategies, such as benchmarking and public reporting or the monitoring of achievements over time.[3 5] There is also some indication in the literature that these strategies can have a positive effect on the quality of care over time.[6 7] For example, a risk-adjusted trend analysis in the US between 2010 and 2019 showed a significant reduction in adverse events, particularly in hospitals that were affected by targeted quality improvement efforts during this period.[8]

One of the quality indicators that could be improved through regular national measurements and targeted quality improvement efforts is inpatient fall rate. Falls in hospital have serious consequences for both patients and the healthcare system as



For peer review only - http://bmjopen.bmj.com/site/about/guidelines.xhtml

a whole.[9 10] Based on data from Australia, a fall in hospital results, on average, in an 8-day longer hospital stay and an additional cost, in purchasing power parity adjusted international dollars,[11] of $4864.[12] Many inpatient falls, though not all, can be prevented with rigorous application of best-practice prevention measures.[13] Therefore, the number of falls in hospital is widely recognised as a relevant indicator of quality of care. Internationally, there are only a few organisations that we are aware of, such as the Press Ganey National Database of Nursing Quality Indicators (NDNQI)[14 15] or the International Prevalence Measurement of Care Quality (LPZ),[16 17] that provide a national infrastructure for collecting data on the quality indicator of falls in hospital.

Since 2011, the LPZ measurement has been conducted annually in Switzerland as a national mandatory quality measurement initiative, commissioned by the Swiss Association for Quality Development in Hospitals and Clinics (ANQ). Alongside other indicators, the prevalence of inpatient falls in all Swiss acute care hospitals is determined using cross-sectional primary data.[18] The aim is multifaceted: to inform a wide range of stakeholders – politicians, hospitals and the general public – about the findings and, in particular, to provide hospitals with the data needed to initiate quality improvement measures.[19] Data collected per measurement is forwarded to the Bern University of Applied Sciences (BFH) for comprehensive national analysis and reporting. The results will ultimately be published on the ANQ website (www.anq.ch), both in the form of a detailed report and a transparent national hospital comparison.[18-20] A recently submitted descriptive analysis based on these data from 2011 to 2019 showed that inpatient fall rates in Switzerland decreased substantially after the first survey and then stagnated over time.[21] However, this putative trend could not be conclusively assessed purely on the basis



For peer review only - http://bmjopen.bmj.com/site/about/guidelines.xhtml

of the descriptive analysis. A decreasing trend in fall rates at the national level might be an indication that the quality of care in hospitals with regard to fall prevention has improved over time. However, a stagnation of fall rates over time would contradict the hypothesis outlined by Berwick, et al. [1] that regular quality measurements favour the initiation of quality improvement processes and thus continuous quality improvement, which would be reflected in decreasing national inpatient fall rates over time. It is however also possible that there are other reasons that favour a stagnation of fall rates in the hospitals and thus possibly mask the effect of regular quality measurements on quality improvement in the hospitals.

- Not all falls are preventable: Even if fall prevention is continuously improved, the fall rates will remain constant at a certain level, as a reduction to zero will not be achievable.[13]
- Lack of resources to intensify fall prevention: Due to increasing financial constraints and staff shortages, fall prevention cannot be further strengthened.[22]
- Patient-related risk factors for falls are increasing: More and more often, older and multimorbid patients are being treated in the hospital, i.e. patients who carry a higher risk of falling. This means that more patients with a higher fall risk profile are being treated in hospital over time. As a result, falls in hospital do not decrease despite constant quality improvements.[21]

In the last scenario, keeping fall rates constant would still be a success.

In order to accurately compare inpatient fall rates over time while accounting for changing patient-related fall risk factors, it is usually recommended to conduct a risk-adjusted comparison.[23] Risk adjustment in this context means that a statistical model is used to control for ongoing changes in patient-related fall risk factors that



For peer review only - http://bmjopen.bmj.com/site/about/guidelines.xhtml

cannot be influenced by hospitals. If patient-related fall risk factors are kept constant over time, it can be ensured that positive/negative trends are actually due to quality improvements/deteriorations in care rather than to changes in patient-related risk factors.

The objectives of this study were therefore:

- First, to investigate whether a trend regarding the prevalence of inpatient falls in acute care hospitals between 2011 and 2019 can be statistically confirmed on a national level in Switzerland.
- Second, whether an identified trend persists after risk adjustment for patient-related fall risk factors.

# METHODS

## Study design

We conducted a secondary data analysis based on multicentre cross-sectional primary data collected annually between 2011 and 2019 as part of the LPZ measurement in Switzerland. This measurement is used to collect data on various quality indicators, such as falls, pressure injuries and malnutrition, in different countries (e.g. the Netherlands, Austria, Switzerland) during a defined data collection period. In Switzerland, usually on the Tuesday of the second week in November. The methods used in the so-called LPZ measurement are described in detail elsewhere.[17 18 24]

## Data collection and population

The LPZ questionnaire was used to collect the primary data.[17 18] This questionnaire emerged from an instrument developed on the basis of a literature



For peer review only - http://bmjopen.bmj.com/site/about/guidelines.xhtml

review, a Delphi and a pilot study for the cross-setting and cross-institutional survey of pressure injuries,[25] which was assessed as reliable and valid.[25-27] Subsequently, other instruments were added to the initial questionnaire to capture further quality indicators, such as falls, by an international research group and based on evidence-based research. The LPZ questionnaire is divided into three parts: institutional, ward and patient questionnaires. In 2016, it underwent a revision and was subsequently referred to as LPZ 2.0. This revision streamlined the questionnaire by reducing the number of structure and process indicators, while largely preserving the questions related to patient characteristics. Although the revision also affected the questions on quality indicators, these changes involved a reduction in the number of questions rather than a change in content. For instance, regarding the quality indicator on falls, specific context questions on the time and place of the fall and the main causes were deleted. Detailed descriptions of the adjustments made to the "inpatient fall" variable can be found in the paragraph "Outcome variable".

Data collection in the hospitals was organised and coordinated by designated hospital coordinators, who were trained during annual national training events using the train-the-trainer principle, so that they could subsequently train their clinical data collection teams on site. To additionally ensure uniform data collection, a detailed measurement manual with definitions, instructions, explanations and examples was available to all persons involved. The clinical data collection teams each consisted of two nurses. They gathered the required data by directly questioning the patient or, where permissible, from the patient's documentation on the survey day and then entered it into the web-based data entry programme.



For peer review only - http://bmjopen.bmj.com/site/about/guidelines.xhtml

In Switzerland, all acute care hospitals with an acute somatic service mandate have been obliged to collect data on at least pressure injuries and inpatient falls once a year in November since 2011. Other institutions were allowed to participate in the LPZ measurement on a voluntary basis. In 2020 and 2021, no LPZ measurement took place in Switzerland due to the Covid pandemic. At ward level, all wards were included, with the exception of emergency departments, outpatient wards and recovery rooms as of 2012, and additionally, maternity wards as of 2013. On the survey day, all inpatients were included in the LPZ measurement. In addition, written consent had to be obtained from patients or their legal representatives for inclusion in 2011, and verbal consent from 2012 onwards (see ethics statements). The hospital coordinators were responsible for ensuring that all patients or, if they were incapacitated, their legal representatives were informed in advance, in writing, about the aim and procedure of the measurement. The written/oral consent of the patients was clarified directly at the time of the survey, that of the legal representatives in advance.

For the national analysis and reporting, the BFH adjusted the raw LPZ dataset for each measurement to fulfil the inclusion and exclusion criteria specified by ANQ for national reporting.

The following adjustments were made:

- Exclusion of institutions that do not have an acute somatic service mandate, such as psychiatric, rehabilitation and geriatric clinics.
- Exclusion of patients under the age of 18.

**Study population**



For peer review only - http://bmjopen.bmj.com/site/about/guidelines.xhtml

For the present study, we had full access to the national analysis datasets of the BFH from 2011 to 2019, to which the following general adjustments were made for the current secondary data analysis:

- Exclusion of conflicting cases.
- Exclusion of patients for whom it was unknown whether they had fallen in the institution in the last 30 days.
- Exclusion of non-participating patients from whom only the reason for non-participation was recorded.
- Exclusion of cases due to missing information on relevant patient-related fall risk variables.

See also supplementary figure S1 for a flowchart depicting the selection of cases for analysis.

**Outcome variable**

The outcome variable “inpatient fall” is defined in the LPZ measurement as “any unintentional change in position that results in the client coming to rest on the ground or other lower level, regardless of the reason”.[17 28]

To determine whether or not a fall occurred in the hospital, the assessment was carried out retrospectively by the clinical data collection teams on the day of the survey. The assessment process involved three steps: first, the patients were asked; second, the nurse in charge was consulted; and third, patient documentation was reviewed for any recorded falls.

The following question(s) with specified response categories had to be answered:

- From 2011 to 2015:



For peer review only - http://bmjopen.bmj.com/site/about/guidelines.xhtml

"How often has the patient fallen in the last 30 days?" with responses categorized as once (1), twice (2), thrice (3), more than three times (4), unknown (5), and not fallen (6).

"When was the most recent fall?" with responses categorized as before admission in current health care facility (1) or after admission (2).

- From 2016 to 2019, after revision of the LPZ questionnaire, the questions were simplified to:

  "Has the patient fallen in the last 30 days in this institution?" with responses being no (0), yes (1), and unknown (97).

Based on the raw variables, the outcome variable "inpatient fall" was calculated as follows for the present study:

- From 2011 to 2015:

  The variable on the frequency of falls in the last 30 days was recoded as follows: 6 (no fall) to 0; 1-4 to 0 if variable on timing of the last fall equals 1 (before admission); 1-4 to 1 if variable for timing of the last fall equals 2 (after admission). In this way, a new variable was created indicating whether the patient had fallen (yes [1] or no [0]) in the last 30 days in this institution. In addition, due to the lack of information, all cases with "unknown" (5) concerning the question on the frequency of falls in the last 30 days were excluded from the study sample.

- From 2016 to 2019:

  The new variable indicating whether the client had fallen (no or yes) in the last 30 days in this institution was created by recoding the original variable no (0) to 0, yes (1) to 1, and unknown (97) to missing. In addition, all cases with missing values on the newly created variable were excluded from the study



For peer review only - http://bmjopen.bmj.com/site/about/guidelines.xhtml

sample due to missing information on whether the patient had fallen in the last 30 days in this institution.

### Patient-related fall risk variables (covariates)

The LPZ questionnaires used between 2011 and 2019 were studied to identify potential patient-related characteristics that could be used for risk adjustment. To be considered as potential variables, they had to fulfil the following criteria:

- The variable was collected on all survey dates.
- The variable had not undergone any significant changes in content over the course of the survey years (in some cases, minor linguistic changes were accepted)

The following patient-related characteristics were identified as possible variables for risk adjustment: Age, sex, surgical procedure within 14 days prior to measurement day, care dependency according to the Care Dependency Scale (CDS), ICD-10 (International Statistical Classification of Diseases and Related Health Problems 10th Revision[29]) diagnosis groups. For more information on the variables used in this study, see **Table 1**.

**Table 1.** Overview of the variables used in the study



For peer review only - http://bmjopen.bmj.com/site/about/guidelines.xhtml

| Outcome variable | Answer options |
|---|---|
| Has the client fallen in the last 30 days in this institution | no [0] / yes [1] |
| **Patient-related variables** | **Answer options** |
| Age (in years) | scale |
| Sex | male [0] / female [1] |
| Surgical procedure within 14 days prior to measurement | no [0] / yes [1] |
| Care Dependency Scale (CDS)* | care independent (70–75) [0]<br>to a great extent independent (60–69) [1]<br>partially dependent (45–59) [2]<br>to a great extent dependent (25–44) [3]<br>completely dependent (15–24) [4] |
| ICD-10 – Diseases of the circulatory system | no [0] / yes [1] |
| ICD-10 – Diseases of the musculoskeletal system and connective tissue | no [0] / yes [1] |
| ICD-10 – Endocrine, nutritional and metabolic diseases | no [0] / yes [1] |
| ICD-10 – Diseases of the genitourinary system | no [0] / yes [1] |
| ICD-10 – Diseases of the digestive system | no [0] / yes [1] |
| ICD-10 – Diseases of the respiratory system | no [0] / yes [1] |
| ICD-10 – Neoplasms | no [0] / yes [1] |
| ICD-10 – Mental, behavioural and neurodevelopmental disorders | no [0] / yes [1] |
| ICD-10 – Diseases of the blood and blood-forming organs | no [0] / yes [1] |
| ICD-10 – Diseases of the nervous system | no [0] / yes [1] |
| ICD-10 – Certain infectious and parasitic diseases | no [0] / yes [1] |
| ICD-10 – Diseases of the skin and subcutaneous tissue | no [0] / yes [1] |
| ICD-10 – Injury, poisoning and certain other consequences of external causes | no [0] / yes [1] |
| ICD-10 – Diseases of the eye and adnexa AND ICD-10 – Diseases of the ear and mastoid process** | no [0] / yes [1] |
| ICD-10 – Congenital malformations, deformations and chromosomal abnormalities | no [0] / yes [1] |
| **Time variable** | **Values** |
| Time (survey year) | 2011 [1] / 2012 [2] / 2013 [3] / 2014 [4] / 2015 [5] / 2016 [6] / 2017 [7] / 2018 [8] / 2019 [9] |

*The Care Dependency Scale (CDS) is composed of 15 questions that are rated on a scale from 1 to 5. The total score ranges from 15 to 75 points, with a lower score indicating higher care dependency.[30 31] The following five categories can be derived from the sum score: completely dependent on care from others, to a great extent dependent, partially dependent, to a great extent independent and care independent.[32 33]

**These ICD-10 diagnosis groups were only recorded separately from 2013 onwards and were therefore combined for the analyses in order to standardise them.

## Data analysis

The datasets from each of the nine survey years were prepared by standardizing the variable names, descriptions, and labels. Following this, case exclusion criteria were applied consistently, as detailed in the flowchart in supplementary figure S1, and the outcome variable was calculated uniformly across all datasets. After merging the



For peer review only - http://bmjopen.bmj.com/site/about/guidelines.xhtml

For peer review only

datasets into one overall dataset by adding a time variable, the sample was first described by means of frequencies and percentages, and age by means of median and interquartile range (IQR) due to the skewed distribution. Furthermore, the national prevalence of falls in hospital between 2011 and 2019 was estimated as described by Thomann, et al. [20]. For this purpose, using the dataset for the corresponding year, the number of patients with a fall in hospital was divided by the total number of patients in the dataset and then multiplied by 100 to obtain the inpatient fall rate as a relative frequency in per cent.

Second, to investigate whether a trend in the prevalence of inpatient falls in acute care hospitals could be statistically confirmed at the national level in Switzerland between 2011 and 2019, we started the analysis with the simple and still frequently used Cochrane-Armitage trend test. This test is suitable for identifying a linear trend or association between an ordinal dependent variable (time treated as an ordinal variable here) and a binary dependent variable, where the null hypothesis is that there is no trend,[34] i.e., that fall rates have remained the same over time. For purposes of visualisation, a linear trend line was plotted based on a linear regression.

Third, to determine whether an identified trend persists after risk adjustment for patient-related fall risk factors, we performed steps a to f, with a to d covering risk adjustment model development and e to f describing reporting and visualisation:

a) As a starting point for setting up the risk adjustment model, we performed a logistic regression resulting in model "A", which included all available patient-related variables (see **Table 1**) as covariates and, to depict a trend, the time variable as a numerical covariate (coded from 1 to 9, where 1 denotes survey year 2011 and 9 survey year 2019). As it is rather unlikely that an increase or



For peer review only - http://bmjopen.bmj.com/site/about/guidelines.xhtml

decrease will remain constant over the years, a quadratic time-effect was also included in model "A" in order to be able to take account of a non-linear relationship. Additionally we tested for interaction effects between patient-related fall risk variables and the time variable.

b) A stepwise backward variable selection algorithm based on the Akaike Information Criterion (AIC)[35] was then applied to model "A" to determine the relevant patient-related fall risk factors to be included in the risk adjustment model and to check for the presence of a linear or a non-linear trend. The resulting model was designated as model "B".

c) Since the data have a hierarchical structure (patients grouped in hospitals), the selected variables according to model "B" were modelled as fixed effects and the hospitals as a random effect in a two-level random intercept logistic regression model (model "C"). We wanted to make inferences regarding the model parameters of a random-effects model, not any finite population characteristics. This model-based approach, in contrast to a design-based approach, allowed us to generalise to a hypothetical population beyond the data set under consideration.[36]

d) To determine whether the model complexity of model "C" could be further reduced, another two-level random intercept logistic regression model (model "D") was calculated. In model "D", the hospitals were modelled as a random effect and the statistically significant time-related factors as well as the patient-related fall risk factors of model "C" were included as fixed effects. If the ANOVA test revealed no significant difference in the model fit between model "C" and the reduced model "D", model "D" served as the final risk adjustment model that informed the subsequent steps e to f.



For peer review only - http://bmjopen.bmj.com/site/about/guidelines.xhtml

e) To determine whether the patient-related fall risk factors changed over time and therefore to get an idea of whether risk adjustment was necessary at all, the patient-related fall risk factors selected in model "D" (without time-related factors) were included as covariates in a two-level random intercept logistic regression model (model "E"). The need for risk adjustment is indicated if the patient-related fall risk factors have increased or decreased over time. Such an increase/decrease can be confirmed if the national inpatient fall rates increase/decrease over time when predicted solely on the basis of the selected patient-related fall risk factors. The risk-adjusted national inpatient fall rates predicted based on model "E" were graphically plotted over time for visualisation purposes.

f) Finally, to demonstrate the predicted risk-adjusted trend in national fall rates in hospitals over time, the predicted risk-adjusted trend was graphically plotted on the basis of model "D". By controlling for the average effect of patient-related fall risk factors over time, changes in these factors can be largely excluded as a reason for a significant increasing/decreasing trend in the predicted national fall rates. Therefore, an increase/decrease, if any, would more likely be due to a deterioration/improvement in the quality of care provided in hospitals over time.

Fourth, an additional analysis was conducted by repeating the analyses on a subsample of patients aged 65 years and older. This subsample was selected because older patients in particular are more often subject to falls in hospital.

Fifth, as part of a sensitivity analysis, we repeated the complete data analysis using only hospitals that provided data in all nine survey years to assess for potential bias in the results from hospitals that had joined or left the measurement during the study



For peer review only - http://bmjopen.bmj.com/site/about/guidelines.xhtml

period. This approach was based on the assumption that the introduction of annual national quality measurement could trigger quality improvement initiatives in participating hospitals, potentially reducing fall rates over time. However, this trend could be masked by newer hospitals participating in the measurement, as they may initially have higher fall rates compared to hospitals already participating. The aim of the sensitivity analysis was therefore to detect such a masking effect, if one existed.

Data cleaning and merging of the datasets was carried out with IBM© SPSS© Statistics (version 28). All analyses and graphics were carried out with the statistics programme R, version 4.1.0[37] and the packages "DescTools",[38] "MASS",[39] "lme4",[40] "effects"[41 42] and "sjplot"[43]. In the analyses, a p-value < .05 was set as the statistical significance level.

For peer review only

### Patient and public involvement

None.

## RESULTS

### Sample description

A total of 155782 inpatients were recorded in 224 Swiss acute care hospitals on the survey days over the 9 survey years. Of these, 110892 patients were included in the present study sample, yielding an average case inclusion rate of 71.2%. The case inclusion rate was lowest in 2011, with 36.1%, and highest in 2016, with 76.4%. The primary reasons for case exclusions were patient non-participation due to refusal, unavailability at the time of survey, or cognitive impairment. Additionally, missing information on care dependency led to the exclusion of 4726 patients in 2011. Therefore, the results presented below are based on a total of 110892 cases from



For peer review only - http://bmjopen.bmj.com/site/about/guidelines.xhtml

398 222 hospitals. Patient characteristics of the whole sample are described in **Table 2**
399 and by survey year in the supplementary table S1.

400 **Table 2.** Description of the patient characteristics of the sample

| **Patient characteristics** | *n* = 110892 | % |
|---|---|---|
| Sex [female] | 56014 | 50.5 |
| Care dependency (CDS) | | |
| care independent [70-75] | 59874 | 54.0 |
| to a great extent independent [60-69] | 25970 | 23.4 |
| partially dependent [45-59] | 15508 | 14.0 |
| to a great extent dependent [25-44] | 6992 | 6.3 |
| completely dependent [15-24] | 2548 | 2.3 |
| Surgical procedure within 14 days prior to measurement [yes] | 47233 | 42.6 |
| ICD-10 – Diseases of the circulatory system [yes] | 58986 | 53.2 |
| ICD-10 – Diseases of the musculoskeletal system and connective tissue [yes] | 42349 | 38.2 |
| ICD-10 – Endocrine, nutritional and metabolic diseases [yes] | 34244 | 30.9 |
| ICD-10 – Diseases of the genitourinary system [yes] | 32079 | 28.9 |
| ICD-10 – Diseases of the digestive system [yes] | 27884 | 25.1 |
| ICD-10 – Diseases of the respiratory system [yes] | 25600 | 23.1 |
| ICD-10 – Neoplasms [yes] | 21167 | 19.1 |
| ICD-10 – Mental, behavioural and neurodevelopmental disorders [yes] | 19827 | 17.9 |
| ICD-10 – Diseases of the blood and blood-forming organs [yes] | 15461 | 13.9 |
| ICD-10 – Diseases of the nervous system [yes] | 13494 | 12.2 |
| ICD-10 – Certain infectious and parasitic diseases [yes] | 13494 | 12.2 |
| ICD-10 – Diseases of the eye and adnexa OR ICD-10 – Diseases of the ear and mastoid process [yes] | 8148 | 7.3 |
| ICD-10 – Diseases of the skin and subcutaneous tissue [yes] | 7572 | 6.8 |
| ICD-10 – Injury, poisoning and certain other consequences of external causes [yes] | 7559 | 6.8 |
| ICD-10 – Congenital malformations, deformations and chromosomal abnormalities [yes] | 625 | 0.6 |
| | Median | IQR |
| Age [in years] | 70 | 24 |

401

402 **Unadjusted trend in national inpatient fall rates**



For peer review only - http://bmjopen.bmj.com/site/about/guidelines.xhtml

The national inpatient fall rate over the 9 years of the survey was 3.7%. As **Figure 1** shows, the national inpatient fall rate was highest in 2011, at 4.6%, and lowest in 2015, at 3.0%. At the last survey in 2019, the fall rate was 3.7%. Using the Cochran-Armitage trend test, a significant linear decreasing trend over time was found (*p* for trend = 0.004). This means that the national fall rate decreased linearly over time when patient-related fall risk factors were not adjusted for. The estimated linear trend line (see also **Figure 1**) intersects the intercept y at a predicted value of 4.15% in 2011 and reaches a predicted value of 3.41% in 2019.

The national inpatient fall rate in 2015 deviates from the rates of the other survey years. In a sensitivity analysis we excluded the data from year 2015 and checked whether the results of the Cochran-Armitage trend test would become significantly different or not. Since a significant linear decreasing trend could be found both with and without the data from 2015, these were retained in the following analyses.

**Risk-adjusted trend of Swiss national inpatient fall rate**

When patient-related fall risk factors were controlled for, a statistically significant non-linear trend, indicated by the negative coefficient (coeff.) of the time variable (-0.10, *SE* 0.03, *p*=0.001) and the positive coefficient of the squared time variable (0.01, *SE* 0.00, *p*=0.044) in the model, was observed in national inpatient fall rates (**Table 3**). The model indicates that the relationship between time and inpatient falls at national level is not linear, as the decreasing trend flattens out over time. The calculated odds ratios (supplementary table S2) illustrate the observed non-linear trend in that, for example, at the national level, the odds of falling in hospital decreased by 8% in 2012 compared to 2011 (*OR* 0.92, 95% *CI* 0.88–0.96). In contrast, however, the odds neither decreased nor increased in 2019 relative to 2018 (*OR* 1.00, 95% *CI* 0.96–1.04).



For peer review only - http://bmjopen.bmj.com/site/about/guidelines.xhtml

The trend described above was observed while controlling for a total of ten patient-related fall risk variables in the risk adjustment model (**Table 3**): eight significantly *fall risk-increasing* patient-related variables, such as age (*OR* 1.02, 95% *CI* 1.01–1.02, $p < 0.001$), being heavily care dependent (*OR* 5.69, 95% *CI* 5.10–6.36, $p < 0.001$) or in the ICD-10 diagnosis group Mental and Behavioural disorders (*OR* 1.79, 95% *CI* 1.66–1.92, $p < 0.001$); two significantly *fall risk-reducing* patient-related characteristics, sex (*OR* 0.82, 95% *CI* 0.77–0.87, $p < 0.001$) and surgery in the last 14 days prior to measurement (*OR* 0.61, 95% *CI* 0.57–0.66, $p < 0.001$).

For peer review only

**Table 3.** Overview of the two-level random intercept logistic regression model used to derive the risk-adjusted trend in Swiss national inpatient fall rates

| | **Risk-adjusted trend in national inpatient fall rates** | | | | |
|---|---|---|---|---|---|
| *Predictors* | *coeff.* | *SE* | *p-value* | *OR* | *95% CI* |
| (Intercept) | -5.00 | 0.12 | **<0.001** | - | - |
| **Time related factors (trend)** | | | | | |
| Time | -0.10 | 0.03 | **0.001** | - | - |
| (Time)^2 | 0.01 | 0.00 | **0.044** | - | - |
| **Patient related fall risk factors** | | | | | |
| Age [in years] | 0.02 | 0.00 | **<0.001** | 1.02 | 1.01 – 1.02 |
| CDS [care independent (70-75)] | Ref. | | | | |
| CDS [to a great extent independent (60-69)] | 1.04 | 0.05 | **<0.001** | 2.83 | 2.57 – 3.11 |
| CDS [partially dependent (45-59)] | 1.42 | 0.05 | **<0.001** | 4.15 | 3.76 – 4.58 |
| CDS [to a great extent dependent (25-44)] | 1.74 | 0.06 | **<0.001** | 5.69 | 5.10 – 6.36 |
| CDS [completely dependent (15-24)] | 1.32 | 0.09 | **<0.001** | 3.73 | 3.15 – 4.42 |
| ICD-10 – Mental and Behavioural disorders [yes] | 0.58 | 0.04 | **<0.001** | 1.79 | 1.66 – 1.92 |
| ICD-10 – Neoplasms [yes] | 0.37 | 0.04 | **<0.001** | 1.45 | 1.35 – 1.56 |
| ICD-10 – Diseases of the nervous system [yes] | 0.28 | 0.04 | **<0.001** | 1.32 | 1.22 – 1.44 |
| ICD-10 – Diseases of the blood and blood-forming organs [yes] | 0.21 | 0.04 | **<0.001** | 1.23 | 1.13 – 1.33 |
| ICD-10 – Injury, poisoning, other consequences of external causes [yes] | 0.15 | 0.06 | **0.007** | 1.16 | 1.04 – 1.30 |
| ICD-10 – Endocrine, nutritional and metabolic diseases [yes] | 0.13 | 0.03 | **<0.001** | 1.13 | 1.06 – 1.21 |
| Sex [female] | -0.20 | 0.03 | **<0.001** | 0.82 | 0.77 – 0.87 |
| Surgical procedure within 14 days prior to measurement [yes] | -0.49 | 0.04 | **<0.001** | 0.61 | 0.57 – 0.66 |
| **Random Effects** | | | | | |
| $\tau_{00}$ [variability in hospital intercepts] | | | 0.08 | | |
| N [hospitals] | | | 222 | | |
| Observations | | | 110892 | | |

Significant *p*-values are highlighted in bold.

**Figure 2** illustrates the predicted national fall rates per survey year when the following are adjusted for: a) only patient-related fall risk factors (red line), or, b)



For peer review only - http://bmjopen.bmj.com/site/about/guidelines.xhtml

patient-related fall risk factors as well as a non-linear time trend (green line). The red line shows that patient-related fall risk factors have tended to increase over time, which is reflected in increasing predicted risk-adjusted national fall rates. These are estimated to be 3.65% in year 2011 and 4.04% in year 2019. The increase in patient-related fall risk factors over time is also evident from the supplementary table S1. For 8 of the 10 variables included in the risk adjustment model, it was shown descriptively that the patient-related fall risk variables increased over the years, reaching their highest values in 2019, and concurrently the lowest values for female sex and percentage of completely care-independent patients in 2019. For example, the ICD-10 diagnosis group "Mental, behavioural and neurodevelopmental disorders" increased from 15.6% in 2011 to 20.5% in 2019. This underscores the necessity of risk adjustment. The green line shows a decrease followed by a slight increase in predicted national inpatient fall rates over time when changes in patient-related fall risk factors as well as a non-linear time trend are adjusted for. The predicted fall rates based on this model are highest at 4.44% in 2011, lowest at 3.43% in 2016, and 3.73% in 2019. As none of the 95% confidence intervals of the calculated odds ratios (supplementary table S2) is above 1, the slight increase observed visually is not statistically significant.

In the additional analysis, a subsample consisting of 67336 patients aged 65 years or older from 221 hospitals was considered. The results based on this subsample do not differ significantly from the overall sample: Although the national fall rates are descriptively consistently higher than in the overall sample, the same patient-related fall risk variables were selected into the risk adjustment model and the coefficients of the risk variables in the model were not substantially different (see supplementary table S3, table S4, figure S2 and figure S3).



For peer review only - http://bmjopen.bmj.com/site/about/guidelines.xhtml

### Sensitivity analysis

The sensitivity analysis included 93 hospitals that provided complete data over all nine survey years, with a total of 64038 patients. In contrast to the overall sample, the results based on this subsample show a significant linear decreasing trend in the inpatient fall rates over time, both descriptively and risk-adjusted (supplementary figure S4, supplementary table S5 and supplementary figure S5). Although the quadratic time effect was included in the model during AIC selection, the effect was not significant and was therefore omitted from the final model. In the risk adjustment model, with the exception of the additionally selected variable “ICD-10 - diseases of the circulatory system”, the same patient-related fall risk variables were included in the model, and the coefficients of the risk variables in the model did not differ significantly from the model based on the overall sample (supplementary table S5).

## DISCUSSION

In this study, we investigated whether inpatient fall rates in acute care hospitals in Switzerland have changed at the national level between 2011 and 2019, and whether these changes persist when patient-related fall risk factors are adjusted for. In the unadjusted analysis, a linear decreasing trend in inpatient fall rates at the national level was found across the years surveyed. In contrast, the risk-adjusted analysis (which controlled for the average effect of patient-related factors) revealed a significant non-linear decreasing trend across the same years. In other words, the effect appears to flatten out over time. Similar results, i.e. a non-adjusted decreasing trend in fall rates, were found in studies based on National Database of Nursing Quality Indicators (NDNQI) data in US hospitals over a 27-month as well as six-year time period.[44 45] More recently, another study found that combined inpatient falls



For peer review only - http://bmjopen.bmj.com/site/about/guidelines.xhtml

For peer review only

and pressure injury rates in the US decreased statistically significantly from 2010 to 2019 after risk adjustment.[8]

It is assumed that the negative trend in risk-adjusted national fall rates observed in our study between 2011 and 2019 is largely due to continuous quality improvement of structures and processes for fall prevention in hospitals. This assumption is reinforced by a study which showed that improvements designed to prevent falls were implemented at both the structural and process level in Swiss hospitals during the same time period.[21] For example, the average relative frequency of the number of fall prevention measures applied per patient, such as the evaluation of current medication or the evaluation of aid devices, increased significantly from 12.9% in 2011 to 23.1% in 2019.[21] The impetus for the improvement measures put in place by hospitals is likely to have been facilitated by the well-known quality improvement mechanisms associated with the introduction of national inpatient falls measurement in 2011. Although regular quality measurement alone does not automatically lead to quality improvement,[5] accompanying measures, such as public reporting,[6 46] comparative benchmarking[7] and performance feedback via quality dashboard,[47 48] are described as effective in triggering quality improvement activities in hospitals. The results of the present sensitivity analysis could also fit into this picture, in that hospitals that provided data on all nine survey years were more likely to have succeeded in continuously reducing their fall rates over time compared with the overall sample. It is possible that the regularity of quality measurement has led to a steady impetus to implement internal quality improvement measures. At least anecdotally, we are aware of some internal hospital projects aimed at reducing falls, which were initiated based on the results of the national quality measurement. Although it cannot be ruled out that other large-scale



For peer review only - http://bmjopen.bmj.com/site/about/guidelines.xhtml

initiatives or programmes besides the introduction of the national quality measurement may have contributed to the reduction of falls in hospitals in Switzerland between 2011 and 2019, we are only aware of two fall prevention programmes of national scope implemented either before 2011 or after 2019 led by national organisations.[49 50]

Nevertheless, despite the quality improvement measures implemented by hospitals to prevent falls, the risk-adjusted national fall rates did not, as a whole, decline linearly over time. Instead, they showed a greater decline at the beginning of the surveys and then gradually levelled off, as demonstrated by the significant non-linear effect observed in our study. This pattern was also observed for nosocomial pressure injuries, another quality indicator of patient safety, at least descriptively over time.[16 21 51 52] For example, a decreasing trend that levelled off over time was observed in hospitals in the USA between 2006 and 2019[51] and in Switzerland between 2011 and 2019.[21 52] Therefore, the trend found in our study may not be specific to falls, but may reflect a more general pattern. In principle, however, we can only speculate about the reasons for the non-linear trend found. Generally, it can be assumed that the lower fall rates get, the more difficult it becomes to reduce them further, as not all fall events are preventable.[13] Another explanation could be that, given the increase in patient-related fall risk factors over time, more and more effort is required on the part of hospitals just to keep fall rates constant and, accordingly, it becomes increasingly challenging to reduce them further.[21] This possible link is particularly emphasised by the results of the present study, which showed an increase in patient-related fall risk factors over time based on increasing predicted fall rates at national level when prediction is based on these patient-related fall risk factors (as shown by the red line in **Figure 2**). The



For peer review only - http://bmjopen.bmj.com/site/about/guidelines.xhtml

observation that medical progress may unintentionally be contributing to a rise in risk factors and a concomitant levelling off of positive trends by enabling certain multimorbid patients to survive has already been made with regard to pressure injuries.[21 51]

Almost more important than identifying the reasons for the non-linear trend is the fact that it indicates a potential increase in fall rates at national level in the coming years if the observed trend continues. This development could be exacerbated by a further increase in fall risk factors and patient complexity in hospital, as well as by a shortage of nursing staff, especially registered nurses, which is expected to worsen in the coming years both internationally[22] and in Switzerland[53]. A shortage of registered nurses has already been linked in a meta-analysis to poorer overall patient outcomes[54] and, in an observational study, to higher fall rates in hospital.[55] Under these circumstances, it is strongly recommended that inpatient fall rates should be continuously monitored. Such monitoring, on the one hand, would inform decision-makers at all levels and health professionals about potential deteriorations in the quality of care provided and enable initiation of appropriate data-driven countermeasures; on the other, it would determine whether the non-linear effect found here is confirmed in the future or whether it represents a sample-specific observation.

It should be noted that due to the large sample in our study even small differences become statistically significant. Therefore, as in the study by Bouldin, et al. [44], the question arises whether the observed decline in fall rates over time is also value-based. In our study, we found an absolute reduction in predicted risk-adjusted fall rates of 0.71% between 2011 and 2019. Extrapolated to the slightly more than 1.2 million cases in general hospitals in 2019 in Switzerland,[56] this means that the



For peer review only - http://bmjopen.bmj.com/site/about/guidelines.xhtml

quality improvement measures implemented over the years prevented 8520 fall events in 2019 compared to 2011. This decrease may seem small, but considering the possible impact of a fall on the individual, such as injury or death,[57] this decrease is nevertheless remarkable and of great value to patients. The decrease is also valuable from an economic point of view. If we multiply the prevented fall events by 4864 purchasing power parity adjusted international dollars, the average cost of a fall calculated by Morello, et al. [12], it is estimated that more than 41 million international dollars in healthcare costs could have been saved nationwide in 2019. These savings underscore the economic benefits of investing in fall prevention strategies, for which a good return on investment is assumed.[10] Therefore, the implementation of evidence-based interventions, such as patient and staff education, could help to reduce fall rates, or at least keep them constant.[58] This would not only have a positive impact on the direct cost savings already mentioned, but also on the possibility of reallocating resources to other important areas of the healthcare system.

**Strength and limitations**

A strength of our study is that the data used was collected using a highly standardised, internationally proven method and based on a census survey of all Swiss hospitals; it thus accurately reflects the situation at the national level. However, due to the average case inclusion rate of around 71% and the comparatively low rate of 36.1% in 2011, a possible selection bias in the LPZ measurement and in our study cannot be ruled out. A recall bias cannot be ruled out either, since the patients were asked whether they had fallen in hospital in the last 30 days. As with the selection bias, the recall bias could have led to a potential underestimation of the fall rates observed. Since it can be assumed that the



For peer review only - http://bmjopen.bmj.com/site/about/guidelines.xhtml

underestimation, if present, has remained constant over time, i.e. a systematic underestimation, this would have no influence on the trend found. In general, it should be noted that adjustments can only be made for measurable risk factors for which data are available. Due to the limited number of patient-related fall risk factors collected with the LPZ questionnaire in Switzerland, not all potentially relevant risk factors could be considered in the risk adjustment model. For instance, it should be investigated whether it is possible to reliably collect further data, e.g. on frailty,[59] sarcopenia,[60] malnutrition,[61] impaired mobility,[62] urinary incontinence,[63] polypharmacy,[64] or use of cardiovascular,[65] psychotropic,[66] opioid,[64] antiepileptic[64] medication at national level without great effort. The effects of integrating some of these additional risk factors on the risk adjustment model would also need to be investigated. In addition, due to adjustments in the LPZ questionnaire over time, two relevant fall risk variables, a fall in the last 12 months or the use of sedatives/psychotropic medication (only collected from 2016 onwards) could not be taken into account in the risk adjustment model. Although these variables were not available for the present analyses, on the whole, the same patient-related characteristics with similar coefficients were identified as fall risk variables as in our previous studies based on LPZ data.[18 67] Due to the cross-sectional survey design, establishing a temporal link between the fall risk factors and the outcome is not possible. To enable this link and reduce selection bias, it is recommended that future trend analyses should look for opportunities to draw on data from the entire patient population, where possible. Ideally, this data should cover the entire period from admission to discharge and, where possible, the variables used for risk adjustment should relate to patient-related fall risk factors that were already present on admission. In this respect, electronic medical record data



For peer review only - http://bmjopen.bmj.com/site/about/guidelines.xhtml

could in future provide a suitable data basis for national monitoring of inpatient falls over time, including risk adjustment.[68] However, further research is required.

# CONCLUSIONS

In our study, we found, descriptively, a significant linear decreasing trend in fall rates in Swiss hospitals and, after adjustment for patient-related fall risk factors, a significant non-linear decreasing trend, i.e., a decrease that flattens out over time. It can be assumed that the introduction of the annual national measurement of inpatient falls in 2011 triggered improvement measures in hospitals in Switzerland, which in turn manifested in lower fall rates over time. The quality improvement achieved has had positive effects both for patients and for society as a whole by improving health and saving costs. However, the identified non-linear effect also showed that the fall rates decreased more at the beginning of the measurements than later on. This could be an indication that fall rates may increase in the future, especially in light of future challenges such as increasing fall risk factors, as shown in our study, and the staff shortages that hospitals are facing. The extent to which the observed non-linear trend will be confirmed in the future requires further investigation. In this context, it is recommended that national policy makers continue to collect and monitor the fall rates in hospitals, on the one hand because this enables data-based quality development in the hospitals, and on the other so that a possible increase in national fall rates can be recognised at an early stage and appropriate targeted quality-improvement measures can be planned and implemented at national level. For this, robust, reliable and fair comparisons over time are important. Risk adjustment can be recommended as a method for improving the comparability of results over time. To address the limitations of the



For peer review only - http://bmjopen.bmj.com/site/about/guidelines.xhtml

present study, it is also recommended to further investigate to what extent routine clinical data are suitable as a basis for national comparisons of fall rates, including risk adjustment for patient-related fall risk factors to ensure comparability over time.

## Acknowledgements

We would like to thank all hospitals and patients who participated in the surveys.

## Competing interests

None declared.

## Funding

The annual national measurement of inpatient falls in Switzerland and thus the data collection was financed by the Swiss National Association for Quality Development in Hospitals and Clinics (ANQ) and carried out under mandate by the Bern University of Applied Sciences (BFH). The present data analysis was also financed by ANQ (Contract no. "Kompensationsleistungen 2020").

## Data availability statement

The data on which the present results are based can be obtained on reasonable request from the Swiss National Association for Quality Development in Hospitals and Clinics (ANQ) (see www.anq.ch). However, the availability of these data may be subject to restrictions in accordance with the "Empfehlungen Verwendung von ANQ-Daten zu Forschungszwecken [Recommendations for the use of ANQ data for research purposes]", which can be found at www.anq.ch.



For peer review only - http://bmjopen.bmj.com/site/about/guidelines.xhtml

# Ethics statements

## Patient consent for publication

Not applicable.

## Ethics approval

This study was conducted in accordance with the ethical principles of the Declaration of Helsinki. Full Research Ethics Committee approval for the primary data collection was granted by the lead Cantonal Ethics Committee Bern (Kantonale Ethikkommission Bern) on 4 October 2011 (application no. 122/11) in agreement with the other twelve local cantonal ethics committees:

- Kantonale Ethikkommission Thurgau
- Kantonale Ethikkommission Luzern
- Kantonale Ethikkommission Aargau
- Kantonale Ethikkommission Zürich
- Ethikkommission beider Basel
- Ethikkommission des Kantons St. Gallen
- Ethikkommission Appenzell Ausserhoden (AR)
- Commission d'éthique pour la recherche clinique en ambulatoire dans le canton de Genève
- Commission d'éthique de recherche du canton de Fribourg
- Commission cantonale valaisanne d'éthique médicale
- Commission cantonale d'éthique de la recherche sur l'être humain
- Comitato etico cantonale Ticino.



For peer review only - http://bmjopen.bmj.com/site/about/guidelines.xhtml

681 As of 2012, Swiss ethics (Swiss Association of Research Ethics Committees) and the cantonal ethics committees declared the measurement as a quality measurement, which does not require ethical approval because it is not subjected to the Swiss Human Research Act. In the course of the reclassification of the measurement as a quality measurement, the ethics committees also agreed that written patient consent is no longer required and therefore written patient information followed by oral consent from the patients or their legal representatives is sufficient. Therefore, all patients or their legal representatives gave at least their verbal informed consent to participate in the measurement (see Bernet, et al. [18] for more details). Furthermore, in 2022, the Ethics Advisory Board of the Bern University of Applied Sciences declared that the anonymised data from the LPZ measurement could be used again without further approval from a cantonal ethics committee (Ref. EAB2022_012). Only fully anonymised data were used in the present study.

# Contributors

Conceptualization of the measurement: NB, IE, SH, JS; conceptualization of the manuscript: NB, MM; data collection and curation: NB, MM; data analysis: NB, MM; data interpretation: NB, IE, SH, MM, JS; writing – original draft preparation: NB; writing – review and editing: IE, SH, MM, JS. NB is responsible for the overall content of the manuscript as guarantor.

# References

1. Berwick DM, James B, Coye MJ. Connections Between Quality Measurement and Improvement. *Medical Care* 2003;41(1):I-30-I-38. doi: https://doi.org/10.1097/00005650-200301001-00004
2. Smith PC, Mossialos E, Papanicolas I, et al. Performance Measurement for Health System Improvement: Experiences, Challenges and Prospects. Cambridge: Cambridge University Press 2010.



For peer review only - http://bmjopen.bmj.com/site/about/guidelines.xhtml

3. Busse R, Klazinga N, Panteli D, et al. *Improving healthcare quality in Europe: Characteristics, effectiveness and implementation of different strategies.* OECD WHOa, 2019. Accessed May 17, 2021. https://apps.who.int/iris/bitstream/handle/10665/327356/9789289051750-eng.pdf?sequence=1&isAllowed=y.

4. Hibbard JH, Stockard J, Tusler M. Hospital Performance Reports: Impact On Quality, Market Share, And Reputation. *Health Affairs* 2005;24(4):1150-60. doi: https://doi.org/10.1377/hlthaff.24.4.1150

5. Agweyu A, Hill K, Diaz T, et al. Regular measurement is essential but insufficient to improve quality of healthcare. *BMJ* 2023;380:e073412. doi: https://doi.org/10.1136/bmj-2022-073412

6. Metcalfe D, Rios Diaz AJ, Olufajo OA, et al. Impact of public release of performance data on the behaviour of healthcare consumers and providers. *Cochrane Database of Systematic Reviews* 2018(9) doi: https://doi.org/10.1002/14651858.CD004538.pub3

7. Willmington C, Belardi P, Murante AM, et al. The contribution of benchmarking to quality improvement in healthcare. A systematic literature review. *BMC Health Services Research* 2022;22(1):139. doi: https://doi.org/10.1186/s12913-022-07467-8

8. Eldridge N, Wang Y, Metersky M, et al. Trends in Adverse Event Rates in Hospitalized Patients, 2010-2019. *Jama* 2022;328(2):173-83. doi: https://doi.org/10.1001/jama.2022.9600

9. LeLaurin JH, Shorr RI. Preventing Falls in Hospitalized Patients: State of the Science. *Clinics in geriatric medicine* 2019;35(2):273-83. doi: https://doi.org/10.1016/j.cger.2019.01.007

10. Slawomirski L, Klazinga N. *The economics of patient safety.* Paris: OECD Publishing, 2022. Accessed February 23, 2024. https://doi.org/10.1787/761f2da8-en.

11. Organisation for Economic Co-operation and Development (OECD). Purchasing power parities (PPP) (indicator) 2024 [Available from: https://doi.org/10.1787/1290ee5a-en accessed February 18, 2024.

12. Morello RT, Barker AL, Watts JJ, et al. The extra resource burden of in-hospital falls: a cost of falls study. *Medical Journal of Australia* 2015;203(9):367. doi: https://doi.org/10.5694/mja15.00296

13. Registered Nurses' Association of Ontario. *Preventing Falls and Reducing Injury from Falls.* Toronto, ON: Registered Nurses' Association of Ontario, 2017. Accessed June 07, 2021. https://rnao.ca/sites/rnao-ca/files/bpg/FALL_PREVENTION_WEB_1207-17.pdf.

14. NDNQI - Nursing excellence made simple: PressGaney; 2023 [Available from: https://www.pressganey.com/platform/ndnqi/ accessed July 11, 2023.

15. Staggs VS, Davidson J, Dunton N, et al. Challenges in defining and categorizing falls on diverse unit types: lessons from expansion of the NDNQI Falls Indicator. *Journal of Nursing Care Quality* 2015;30(2):106-12. doi: https://doi.org/10.1097/ncq.0000000000000085

16. Halfens RJG, Meesterberends E, van Nie NC, et al. International prevalence measurement of care problems: results. *Journal of Advanced Nursing* 2013;69(9):e5-c17. doi: https://doi.org/10.1111/jan.12189

17. Van Nie NC, Schols JMGA, Meesterberends E, et al. An international prevalence measurement of care problems: study protocol. *Journal of Advanced Nursing* 2013;69(9):c18-c29. doi: https://doi.org/10.1111/jan.12190

18. Bernet NS, Everink IHJ, Schols JMGA, et al. Hospital performance comparison of inpatient fall rates; the impact of risk adjusting for patient-related factors: a

For peer review only



For peer review only - http://bmjopen.bmj.com/site/about/guidelines.xhtml

multicentre cross-sectional survey. *BMC Health Services Research* 2022;22(1):225. doi: https://doi.org/10.1186/s12913-022-07638-7

19. Busch P. ANQ-Qualitätsmessungen in Spitälern und Kliniken – quo vadis? *saez* 2019;100(41):1350-52. doi: https://doi.org/10.4414/saez.2019.18271

20. Thomann S, Röösli R, Richter D, et al. *Auswertungskonzept ANQ: Nationale Prävalenzmessung Sturz und Dekubitus.* Bern: Nationaler Verein für Qualitätsentwicklung in Spitälern und Kliniken (ANQ) & Berner Fachhochschule (BFH), 2020. Accessed December 14, 2021. https://www.anq.ch/wp-content/uploads/2017/12/ANQ_Sturz_Dekubitus_Auswertungskonzept.pdf.

21. Thomann S, Zimmermann R, Riedweg JS, et al. National developments in falls and pressure ulcers in Swiss hospitals from 2011-2019: a secondary data analysis of the national quality measurement. *Manuscript submitted for publication* 2023,

22. World Health Organization (WHO). Regional Office for Europe. *Health and care workforce in Europe: time to act.* Copenhagen: World Health Organization. Regional Office for Europe, 2022. Accessed February 23, 2024. https://apps.who.int/iris/handle/10665/362379.

23. Lane-Fall MB, Neuman MD. Outcomes measures and risk adjustment. *International Anesthesiology Clinics* 2013;51(4):10-21. doi: https://doi.org/10.1097/AIA.0b013e3182a70a52

24. Everink I, Gordon A, Rijcken S, et al. Learning and improving in long-term care internationally: the International Prevalence Measurement of Care Quality (LPZ). *Geriatrics, Gerontology and Aging* 2021;15(2021) doi: http://dx.doi.org/10.53886/gga.e0210056

25. Bours GJ, Halfens RJG, Lubbers M, et al. The development of a national registration form to measure the prevalence of pressure ulcers in the Netherlands. *Ostomy Wound Management* 1999;45(11):28-33, 36-8, 40.

26. Gorecki C, Brown J, Cano S, et al. Development and validation of a new patient-reported outcome measure for patients with pressure ulcers: the PU-QOL instrument. *Health and Quality of Life Outcomes* 2013;11(1):95. doi: https://doi.org/10.1186/1477-7525-11-95

27. Halfens RJG, Meesterberends E, Meijers JMM, et al. *Landelijke Prevalentiemeting Zorgproblemen. Rapportage resultaten 2011.* Maastricht: 2011. Accessed June 03, 2021. https://nl.lpz-um.eu/Content/Public/NL/Publications/LPZ%20Rapport%202011.pdf.

28. Kellogg International Work Group on the Prevention of Falls by the Elderly. The prevention of falls in later life. A report of the Kellogg International Work Group on the Prevention of Falls by the Elderly. *Danish medical bulletin* 1987;34(Supplement 4):1-24.

29. World Health Organization. International Statistical Classification of Diseases and Related Health Problems 10th Revision 2016 [Available from: https://icd.who.int/browse10/2016/en accessed January 19, 2023.

30. Dijkstra A, Smith J, White M. *Manual Care Dependency Scale. Measuring care dependency with the Care Dependency Scale (CDS). A manual.* 2006. Accessed December 02, 2019. https://www.care2share.eu/dbfiles/download/29.

31. Lohrmann C, Dijkstra A, Dassen T. The Care Dependency Scale: an assessment instrument for elderly patients in German hospitals. *Geriatric Nursing* 2003 doi: https://doi.org/10.1067/mgn.2003.8

32. Eglseer D, Halfens RJG, Schols JMGA, et al. Dysphagia in Hospitalized Older Patients: Associated Factors and Nutritional Interventions. *Journal of Nutrition, Health and Aging* 2018;22(1):103-10. doi: https://doi.org/10.1007/s12603-017-0928-x



For peer review only - http://bmjopen.bmj.com/site/about/guidelines.xhtml

33. Schüttengruber G, Halfens RJG, Lohrmann C. Care dependency of patients and residents at the end of life: A secondary data analysis of data from a cross-sectional study in hospitals and geriatric institutions. *Journal of Clinical Nursing* 2022;31(5-6):657-68. doi: https://doi.org/10.1111/jocn.15925
34. Armitage P. Tests for Linear Trends in Proportions and Frequencies. *Biometrics* 1955;11(3):375-86. doi: https://www.doi.org/10.2307/3001775
35. Akaike H. A new look at the statistical model identification. *IEEE Transactions on Automatic Control* 1974;19(6):716-23. doi: https://doi.org/10.1109/TAC.1974.1100705
36. Rabe-Hesketh S, Skrondal A. Multilevel and Longitudinal Modeling Using Stata, Volumes I and II. 4 ed. College Station, Texas: Stata Press 2022.
37. R: A Language and Environment for Statistical Computing [program]. Vienna, Austria: R Foundation for Statistical Computing, 2021.
38. DescTools: Tools for Descriptive Statistics [program]. R package version 0.99.48 version, 2023.
39. Venables WN, Ripley BD. Modern Applied Statistics with S. Fourth ed. New York: Springer 2002.
40. Bates D, Mächler M, Bolker B, et al. Fitting Linear Mixed-Effects Models Using lme4. *Journal of Statistical Software* 2015;67(1):1-48. doi: https://doi.org/10.18637/jss.v067.i01
41. Fox J. Effect Displays in {R} for Generalised Linear Models. *Journal of Statistical Software* 2003;8(15):1–27. doi: https://doi.org/10.18637/jss.v008.i15
42. Fox J, Weisberg S. An R Companion to Applied Regression. 3rd ed. Thousand Oaks CA: Sage 2019.
43. sjPlot: Data Visualization for Statistics in Social Science [program], 2021.
44. Bouldin ELD, Andresen EM, Dunton NE, et al. Falls among adult patients hospitalized in the United States: prevalence and trends. *Journal of Patient Safety* 2013;9(1):13-17. doi: https://doi.org/10.1097/PTS.0b013e3182699b64
45. He J, Dunton N, Staggs V. Unit-level Time Trends in Inpatient Fall Rates of US Hospitals. *Medical Care* 2012;50(9):801-07. doi: https://doi.org/10.1097/mlr.0b013e31825a8b88
46. Campanella P, Vukovic V, Parente P, et al. The impact of Public Reporting on clinical outcomes: a systematic review and meta-analysis. *BMC Health Services Research* 2016;16(1):296. doi: https://doi.org/10.1186/s12913-016-1543-y
47. Brown B, Gude WT, Blakeman T, et al. Clinical Performance Feedback Intervention Theory (CP-FIT): a new theory for designing, implementing, and evaluating feedback in health care based on a systematic review and meta-synthesis of qualitative research. *Implementation Science* 2019;14(1):40. doi: https://doi.org/10.1186/s13012-019-0883-5
48. Dowding D, Randell R, Gardner P, et al. Dashboards for improving patient care: Review of the literature. *International Journal of Medical Informatics* 2015;84(2):87-100. doi: https://doi.org/10.1016/j.ijmedinf.2014.10.001
49. Patientensicherheit Schweiz. *Sturzprävention.* Basel/Zürich: Stiftung für Patientensicherheit, 2008. Accessed February 18, 2024. https://patientensicherheit.ch/wp/wp-content/uploads/2023/03/2_SR_2_Sturzpra__vention_D_160725.pdf.
50. PHS Public Health Services. *StoppSturz / Parachutes (2019-2022) – Sturzprävention in der ambulanten Gesundheitsversorgung. Projekt-Schlussbericht der Gesamtprojektleitung.* Bern: PHS Public Health Services, 2023. Accessed February




For peer review only - http://bmjopen.bmj.com/site/about/guidelines.xhtml

18, 2024. https://www.bfu.ch/media/13unxwu3/230314-stoppsturz-schlussbericht_pa.pdf.
51. VanGilder CA, Cox J, Edsberg LE, et al. Pressure Injury Prevalence in Acute Care Hospitals With Unit-Specific Analysis: Results From the International Pressure Ulcer Prevalence (IPUP) Survey Database. *J Wound Ostomy Continence Nurs* 2021;48(6):492-503. doi: https://doi.org/10.1097/won.0000000000000817
52. Thomann S, Schlunegger M, Röösli R, et al. *Sturz und Dekubitus Akutsomatik Erwachsene - Nationaler Vergleichsbericht Messung 2019*. Bern: Nationaler Verein für Qualitätsentwicklung in Spitälern und Kliniken (ANQ) & Berner Fachhochschule (BFH), 2020. Accessed February 01, 2024. https://www.anq.ch/wp-content/uploads/2020/09/ANQakut_Sturz_Dekubitus_Erwachsene_Nationaler-Vergleichsbericht_2019.pdf.
53. Merçay C, Grünig A, Dolder P. *Gesundheitspersonal in der Schweiz – Nationaler Versorgungsbericht 2021. Bestand, Bedarf, Angebot und Massnahmen zur Personalsicherung*. Neuchâtel: Schweizerisches Gesundheitsobservatorium (Obsan), 2021. Accessed February 23, 2024. https://www.obsan.admin.ch/sites/default/files/2021-10/Obsan_03_2021_BERICHT_0.pdf.
54. Dall'Ora C, Saville C, Rubbo B, et al. Nurse staffing levels and patient outcomes: A systematic review of longitudinal studies. *International Journal of Nursing Studies* 2022;134:104311. doi: https://doi.org/10.1016/j.ijnurstu.2022.104311
55. Kim J, Lee E, Jung Y, et al. Patient-level and organizational-level factors influencing in-hospital falls. *Journal of Advanced Nursing* 2022;78(11):3641-51. doi: https://doi.org/10.1111/jan.15254
56. Bundesamt für Gesundheit BAG. *Kennzahlen der Schweizer Spitäler 2021*. Bern: Bundesamt für Gesundheit (BAG), 2023. Accessed February 23, 2024. https://spitalstatistik.bagapps.ch/data/download/kzp21_publication.pdf?v=1679384362.
57. Agency for Healthcare Research and Quality. *Network of Patient Safety Databases Chartbook, 2022*. Rockville, MD: AHRQ Pub, 2022. Accessed February 23, 2024. https://www.ahrq.gov/sites/default/files/wysiwyg/npsd/data/npsd-chartbook-2022.pdf#FallSupp.
58. Morris ME, Webster K, Jones C, et al. Interventions to reduce falls in hospitals: a systematic review and meta-analysis. *Age Ageing* 2022;51(5) doi: https://doi.org/10.1093/ageing/afac077
59. Lan X, Li H, Wang Z, et al. Frailty as a predictor of future falls in hospitalized patients: A systematic review and meta-analysis. *Geriatric Nursing* 2020;41(2):69-74. doi: https://doi.org/10.1016/j.gerinurse.2019.01.004
60. Yeung SSY, Reijnierse EM, Pham VK, et al. Sarcopenia and its association with falls and fractures in older adults: A systematic review and meta-analysis. *Journal of Cachexia, Sarcopenia and Muscle* 2019;10(3):485-500. doi: https://doi.org/10.1002/jcsm.12411
61. Ishida Y, Maeda K, Nonogaki T, et al. Malnutrition at Admission Predicts In-Hospital Falls in Hospitalized Older Adults. *Nutrients* 2020;12(2) doi: https://doi.org/10.3390/nu12020541
62. Zhao YL, Kim H. Older Adult Inpatient Falls in Acute Care Hospitals: Intrinsic, Extrinsic, and Environmental Factors. *Journal of Gerontological Nursing* 2015;41(7):29-43; quiz 44-5. doi: https://doi.org/10.3928/00989134-20150616-05



For peer review only - http://bmjopen.bmj.com/site/about/guidelines.xhtml

63. Moon S, Chung HS, Kim YJ, et al. The impact of urinary incontinence on falls: A systematic review and meta-analysis. *PLOS ONE* 2021;16(5):e0251711. doi: https://doi.org/10.1371/journal.pone.0251711
64. Seppala LJ, van de Glind EMM, Daams JG, et al. Fall-Risk-Increasing Drugs: A Systematic Review and Meta-analysis: III. Others. *Journal of the American Medical Directors Association* 2018;19(4):372.e1-72.e8. doi: https://doi.org/10.1016/j.jamda.2017.12.099
65. de Vries M, Seppala LJ, Daams JG, et al. Fall-Risk-Increasing Drugs: A Systematic Review and Meta-Analysis: I. Cardiovascular Drugs. *Journal of the American Medical Directors Association* 2018;19(4):371.e1-71.e9. doi: https://doi.org/10.1016/j.jamda.2017.12.013
66. Seppala LJ, Wermelink AMAT, de Vries M, et al. Fall-Risk-Increasing Drugs: A Systematic Review and Meta-Analysis: II. Psychotropics. *Journal of the American Medical Directors Association* 2018;19(4):371.e11-71.e17. doi: https://doi.org/10.1016/j.jamda.2017.12.098
67. Bernet NS, Everink IHJ, Hahn S, et al. Comparing risk-adjusted inpatient fall rates internationally: validation of a risk-adjustment model using multicentre cross-sectional data from hospitals in Switzerland and Austria. *BMC Health Services Research* 2024;24(1):331. doi: https://doi.org/10.1186/s12913-024-10839-x
68. Bernet NS, Thomann S, Kurpicz-Briki M, et al. Potential of Electronic Medical Record Data for National Quality Measurement. *Studies in Health Technology and Informatics* 2022;292:51-56. doi: https://doi.org/10.3233/shti220320

For peer review only



For peer review only - http://bmjopen.bmj.com/site/about/guidelines.xhtml

**Figure 1.** Visualisation of the descriptive Swiss national inpatient fall rates from 2011 to 2019 and the estimated linear trend over time

**Figure 2.** Visualisation of predicted risk-adjusted (with and without time trend) Swiss national inpatient fall rates from 2011 to 2019

For peer review only



For peer review only - http://bmjopen.bmj.com/site/about/guidelines.xhtml

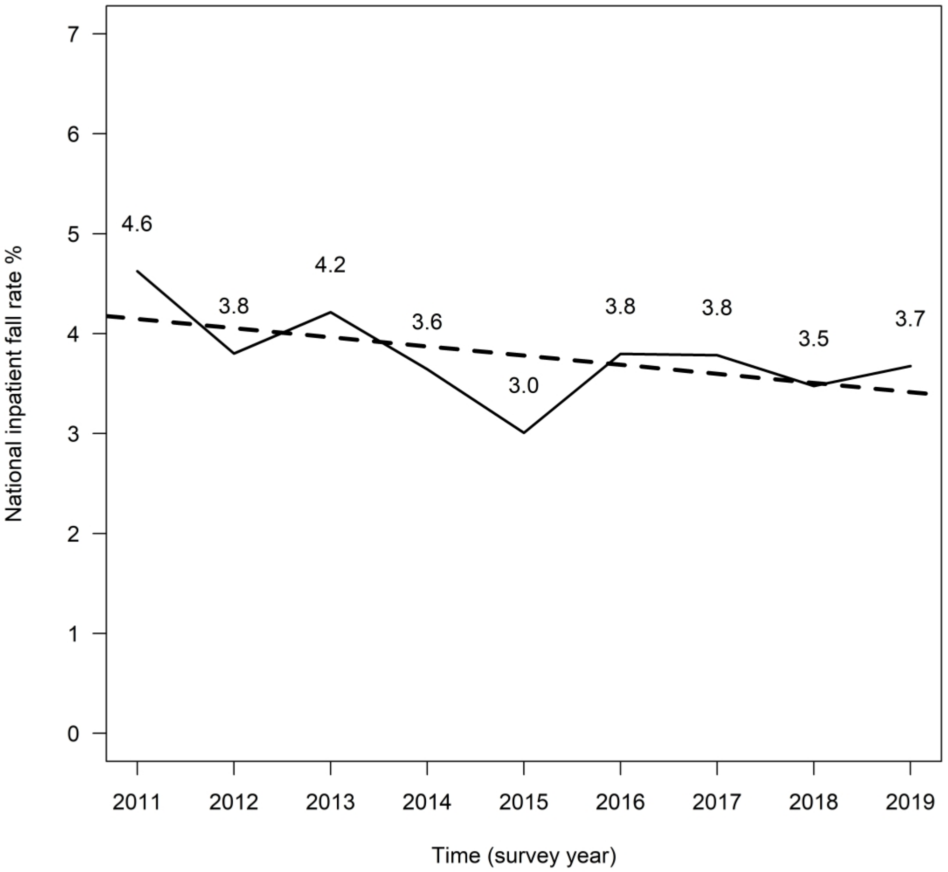


Figure 1: Visualisation of the descriptive Swiss national inpatient fall rates from 2011 to 2019 and the estimated linear trend over time

482x482mm (315 x 315 DPI)

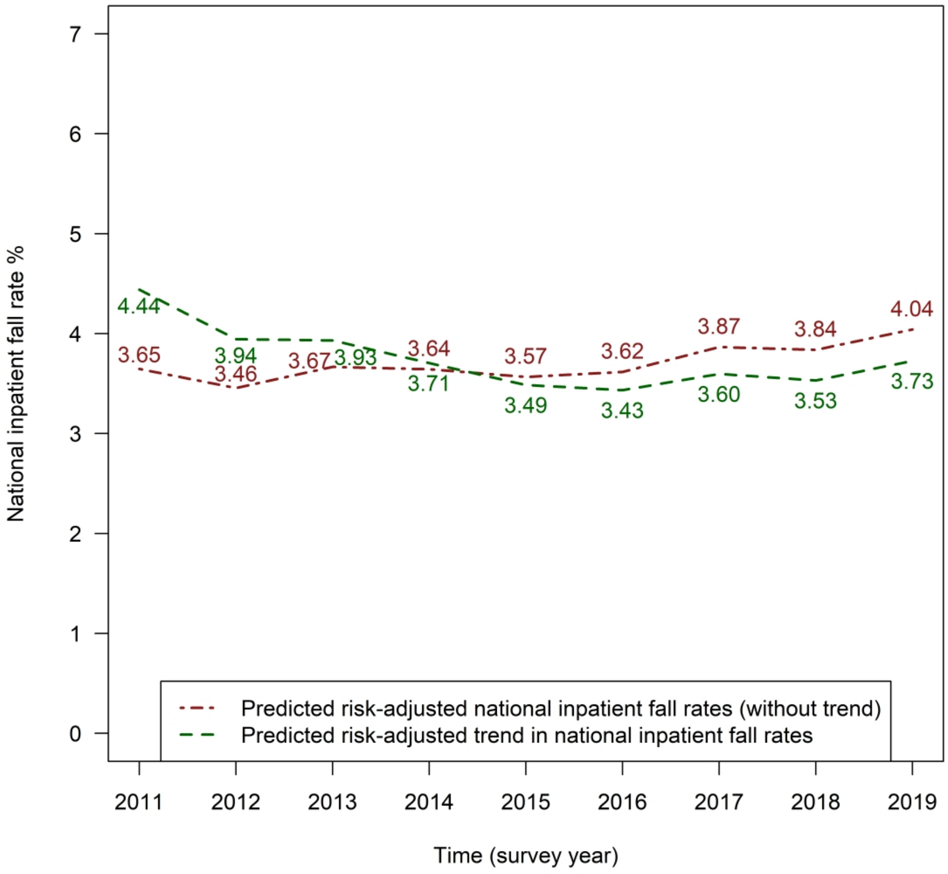


Figure 2: Visualisation of predicted risk-adjusted (with and without time trend) Swiss national inpatient fall rates from 2011 to 2019

482x482mm (315 x 315 DPI)

# Supplementary file

| | | Survey year | 2011 | 2012 | 2013 | 2014 | 2015 | 2016 | 2017 | 2018 | 2019 |
|---|---|---|---|---|---|---|---|---|---|---|---|
| **BFH national analysis dataset** | | *n=* | 15637 | 18105 | 17486 | 17550 | 17233 | 17619 | 17438 | 17250 | 17464 |
| **Exclusion** | Non-comparable hospital/ward types | *n=* | 42 | - | - | - | - | - | - | - | - |
| | Patients younger than 18 years | *n=* | 71 | - | - | - | - | - | - | - | - |
| | Conflicting cases: Non-participation, but data available; participation, but missings on mandatory variables | *n=* | 44 | 6 | 3 | 1 | - | - | - | - | - |
| | Unknown on outcome variable | *n=* | 172 | 203 | 209 | 167 | 158 | 11 | 41 | 78 | 71 |
| | Non-participation | *n=* | 4939 | 4453 | 4580 | 4233 | 4070 | 4154 | 4211 | 4023 | 4224 |
| | No data available on care dependency* | *n=* | 4726 | - | - | - | - | - | - | - | - |
| **Study population** | | *n=* | 5643 | 13443 | 12694 | 13149 | 13005 | 13454 | 13186 | 13149 | 13169 |
| | | *%* | 36.1% | 74.3% | 72.6% | 74.9% | 75.5% | 76.4% | 75.6% | 76.2% | 75.4% |
| **Merged analysis dataset** | | *n=* | | | | | 110892 | | | | |
| | | *%* | | | | | 71.2% | | | | |

*In 2011, the hospitals were free to decide whether to collect the Care Dependency Scale (CDS). In the analyses, 4726 patients from the survey year 2011 therefore had to be excluded due to missing values. No significant group difference was found in the frequency of inpatient falls in the excluded group (no information on CDS available) compared to the included group (information on CDS available) (Chi-Square *p* =0.107).

*Supplementary figure S1: Flowchart depicting the selection of cases for analysis*

*Supplementary table S1: Description of patient characteristics in the sample across the survey years*

| | 2011 *n* = 5643 | 2012 *n* = 13443 | 2013 *n* = 12694 | 2014 *n* = 13149 | 2015 *n* = 13005 | 2016 *n* = 13454 | 2017 *n* = 13186 | 2018 *n* = 13149 | 2019 *n* = 13169 | Total *n* = 110892 |
|---|---|---|---|---|---|---|---|---|---|---|
| **Patient characteristics** | *n* (%) | *n* (%) | *n* (%) | *n* (%) | *n* (%) | *n* (%) | *n* (%) | *n* (%) | *n* (%) | *n* (%) |
| Sex [female] | 3000 (53.2) | 7300 (54.3) | 6400 (50.4) | 6693 (50.9) | 6519 (50.1) | 6646 (49.4) | 6527 (49.5) | 6458 (49.1) | 6471 (49.1) | 56014 (50.5) |
| Care dependency (CDS) | | | | | | | | | | |
| care independent [70-75] | 3106 (55.0) | 7533 (56.0) | 6790 (53.5) | 7182 (54.6) | 7080 (54.4) | 7340 (54.6) | 7036 (53.4) | 7000 (53.2) | 6807 (51.7) | 59874 (54.0) |
| to a great extent independent [60-69] | 1288 (22.8) | 2893 (21.5) | 2963 (23.3) | 2989 (22.7) | 3072 (23.6) | 3236 (24.1) | 3130 (23.7) | 3174 (24.1) | 3225 (24.5) | 25970 (23.4) |
| partially dependent [45-59] | 707 (12.5) | 1844 (13.7) | 1813 (14.3) | 1879 (14.3) | 1767 (13.6) | 1760 (13.1) | 1890 (14.3) | 1888 (14.4) | 1960 (14.9) | 15508 (14.0) |
| to a great extent dependent [25-44] | 408 (7.2) | 883 (6.6) | 850 (6.7) | 814 (6.2) | 783 (6.0) | 776 (5.8) | 817 (6.2) | 792 (6.0) | 869 (6.6) | 6992 (6.3) |
| completely dependent [15-24] | 134 (2.4) | 290 (2.2) | 278 (2.2) | 285 (2.2) | 303 (2.3) | 342 (2.5) | 313 (2.4) | 295 (2.2) | 308 (2.3) | 2548 (2.3) |
| Surgical procedure within 14 days prior to measurement [yes] | 2111 (37.4) | 5336 (39.7) | 5218 (41.1) | 5469 (41.6) | 5708 (43.9) | 5981 (44.5) | 5863 (44.5) | 5774 (43.9) | 5773 (43.8) | 47233 (42.6) |
| ICD-10 – Diseases of the circulatory system [yes] | 2949 (52.3) | 6607 (49.2) | 6453 (50.8) | 6622 (50.4) | 6755 (51.9) | 7275 (54.1) | 7368 (55.9) | 7397 (56.3) | 7560 (57.4) | 58986 (53.2) |
| ICD-10 – Diseases of the musculoskeletal system and connective tissue [yes] | 1876 (33.2) | 4317 (32.1) | 4632 (36.5) | 4919 (37.4) | 5041 (38.8) | 5550 (41.3) | 5373 (40.7) | 5357 (40.7) | 5284 (40.1) | 42349 (38.2) |
| ICD-10 – Endocrine, nutritional and metabolic diseases [yes] | 1476 (26.2) | 3277 (24.4) | 3569 (28.1) | 3788 (28.8) | 4047 (31.1) | 4359 (32.4) | 4522 (34.3) | 4430 (33.7) | 4776 (36.3) | 34244 (30.9) |
| ICD-10 – Diseases of the genitourinary system [yes] | 1578 (28.0) | 3519 (26.2) | 3387 (26.7) | 3522 (26.8) | 3509 (27.0) | 4086 (30.4) | 4085 (31.0) | 4028 (30.6) | 4365 (33.1) | 32079 (28.9) |
| ICD-10 – Diseases of the digestive system [yes] | 1538 (27.3) | 3191 (23.7) | 2991 (23.6) | 3155 (24.0) | 3096 (23.8) | 3417 (25.4) | 3443 (26.1) | 3385 (25.7) | 3668 (27.9) | 27884 (25.1) |
| ICD-10 – Diseases of the respiratory system [yes] | 1175 (20.8) | 2592 (19.3) | 2805 (22.1) | 2837 (21.6) | 3038 (23.4) | 3259 (24.2) | 3241 (24.6) | 3181 (24.2) | 3472 (26.4) | 25600 (23.1) |
| ICD-10 – Neoplasms [yes] | 1014 (18.0) | 2204 (16.4) | 1739 (13.7) | 2426 (18.5) | 2450 (18.8) | 2716 (20.2) | 2836 (21.5) | 2792 (21.2) | 2990 (22.7) | 21167 (19.1) |
| ICD-10 – Mental, behavioural and neurodevelopmental disorders [yes] | 878 (15.6) | 2019 (15.0) | 2222 (17.5) | 2196 (16.7) | 2182 (16.8) | 2485 (18.5) | 2609 (19.8) | 2531 (19.2) | 2705 (20.5) | 19827 (17.9) |
| ICD-10 – Diseases of the blood and blood-forming organs [yes] | 494 (8.8) | 1089 (8.1) | 1663 (13.1) | 1739 (13.2) | 1785 (13.7) | 2083 (15.5) | 2145 (16.3) | 2092 (15.9) | 2371 (18.0) | 15461 (13.9) |
| ICD-10 – Diseases of the nervous system [yes] | 465 (8.2) | 1139 (8.5) | 1333 (10.5) | 1598 (12.2) | 1496 (11.5) | 1813 (13.5) | 1897 (14.4) | 1823 (13.9) | 1930 (14.7) | 13494 (12.2) |
| ICD-10 – Certain infectious and parasitic diseases [yes] | 752 (13.3) | 1672 (12.4) | 1325 (10.4) | 1365 (10.4) | 1386 (10.7) | 1695 (12.6) | 1823 (13.8) | 1663 (12.6) | 1813 (13.8) | 13494 (12.2) |
| ICD-10 – Diseases of the eye and adnexa OR ICD-10 – Diseases of the ear and mastoid process [yes] | 390 (6.9) | 745 (5.5) | 871 (6.9) | 904 (6.9) | 919 (7.1) | 1046 (7.8) | 1091 (8.3) | 1046 (8.0) | 1136 (8.6) | 8148 (7.3) |
| ICD-10 – Diseases of the skin and subcutaneous tissue [yes] | 272 (4.8) | 603 (4.5) | 812 (6.4) | 848 (6.4) | 909 (7.0) | 988 (7.3) | 1031 (7.8) | 1021 (7.8) | 1088 (8.3) | 7572 (6.8) |
| ICD-10 – Injury, poisoning and certain other consequences of external causes [yes] | 631 (11.2) | 1207 (9.0) | 922 (7.3) | 802 (6.1) | 762 (5.9) | 808 (6.0) | 708 (5.4) | 875 (6.7) | 844 (6.4) | 7559 (6.8) |
| ICD-10 – Congenital malformations, deformations and chromosomal abnormalities [yes] | 34 (0.6) | 72 (0.5) | 82 (0.6) | 67 (0.5) | 77 (0.6) | 70 (0.5) | 64 (0.5) | 81 (0.6) | 78 (0.6) | 625 (0.6) |
| | M (IQR) | M (IQR) | M (IQR) | M (IQR) | M (IQR) | M (IQR) | M (IQR) | M (IQR) | M (IQR) | M (IQR) |
| Age [in years] | 69 (25) | 68 (27) | 69 (24) | 69 (24) | 69 (23) | 70 (24) | 70 (24) | 70 (23) | 71 (23) | 70 (24) |

M=Median; IQR=interquartile range.



For peer review only - http://bmjopen.bmj.com/site/about/guidelines.xhtml

*Supplementary table S2: Illustration of the estimated non-linear trend using odds ratios, each of which describes the decrease in Swiss national fall rates in relation to the previous survey year*

| **Survey year** | **2011/2012** | **2012/2013** | **2013/2014** | **2014/2015** | **2015/2016** | **2016/2017** | **2017/2018** | **2018/2019** |
|---|---|---|---|---|---|---|---|---|
| *OR* | 0.92 | 0.93 | 0.94 | 0.95 | 0.97 | 0.98 | 0.99 | 1.00 |
| 95% *CI* | 0.88 – 0.96 | 0.90 – 0.96 | 0.92 – 0.96 | 0.94 – 0.97 | 0.95 – 0.98 | 0.96 – 1.00 | 0.96 – 1.02 | 0.96 – 1.04 |

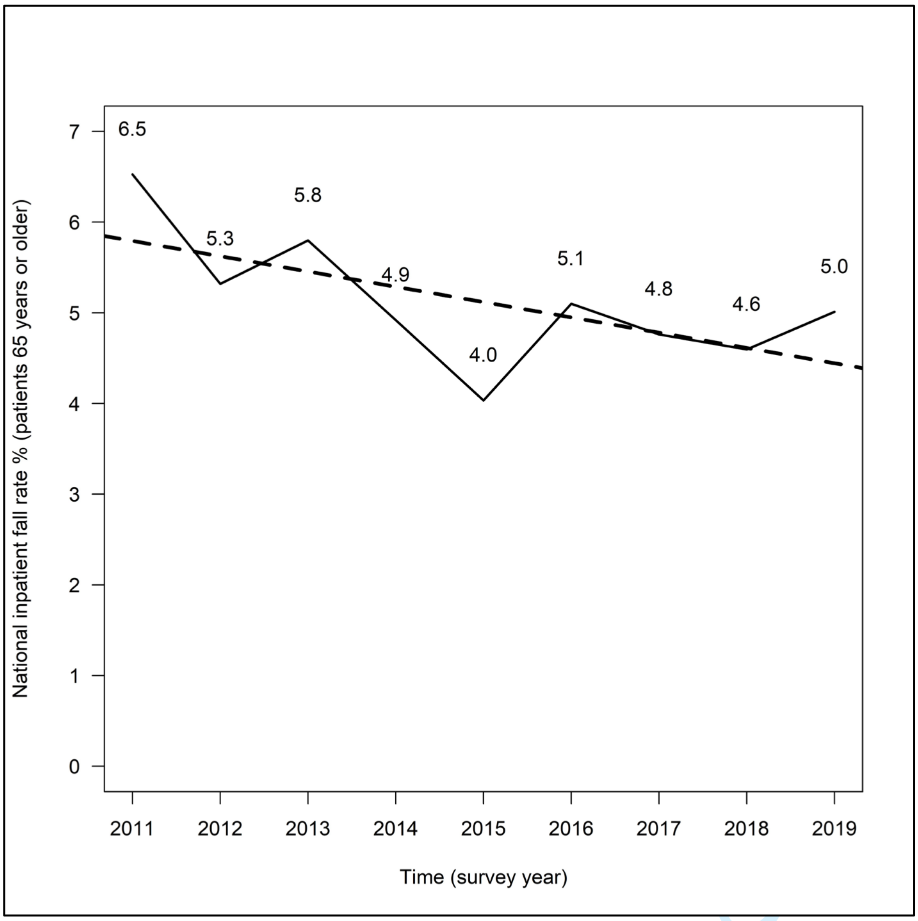


*Supplementary figure S2: Visualisation of the descriptive Swiss national inpatient fall rates from 2011 to 2019 and the estimated linear trend over time, taking into account only patients 65 years old or older*



For peer review only - http://bmjopen.bmj.com/site/about/guidelines.xhtml

*Supplementary table S3: Overview of the two-level random intercept logistic regression model used to derive the risk-adjusted trend in Swiss national inpatient fall rates, taking into account only patients 65 years old or older*

| | **Risk-adjusted trend in national inpatient fall rates** | | | | |
|---|---|---|---|---|---|
| *Predictors* | *coeff.* | *SE* | *p-value* | *OR* | *95% CI* |
| (Intercept) | -4.52 | 0.21 | **<0.001** | - | - |
| **Time related factors (trend)** | | | | | |
| Time | -0.12 | 0.04 | **0.001** | - | - |
| (Time)^2 | 0.01 | 0.00 | **0.020** | - | - |
| **Patient related fall risk factors** | | | | | |
| Age [in years] | 0.01 | 0.00 | **<0.001** | 1.01 | 1.01 – 1.02 |
| CDS [care independent (70-75)] | Ref. | | | | |
| CDS [to a great extent independent (60-69)] | 0.97 | 0.06 | **<0.001** | 2.64 | 2.36 – 2.95 |
| CDS [partially dependent (45-59)] | 1.38 | 0.06 | **<0.001** | 3.96 | 3.54 – 4.43 |
| CDS [to a great extent dependent (25-44)] | 1.66 | 0.06 | **<0.001** | 5.25 | 4.63 – 5.94 |
| CDS [completely dependent (15-24)] | 1.30 | 0.09 | **<0.001** | 3.68 | 3.05 – 4.43 |
| ICD-10 – Mental and Behavioural disorders [yes] | 0.53 | 0.04 | **<0.001** | 1.71 | 1.58 – 1.85 |
| ICD-10 – Neoplasms [yes] | 0.32 | 0.04 | **<0.001** | 1.38 | 1.27 – 1.50 |
| ICD-10 – Diseases of the nervous system [yes] | 0.21 | 0.05 | **<0.001** | 1.24 | 1.13 – 1.36 |
| ICD-10 – Diseases of the blood and blood-forming organs [yes] | 0.21 | 0.04 | **<0.001** | 1.24 | 1.13 – 1.35 |
| ICD-10 – Injury, poisoning, other consequences of external causes [yes] | 0.18 | 0.06 | **0.003** | 1.20 | 1.06 – 1.36 |
| ICD-10 – Endocrine, nutritional and metabolic diseases [yes] | 0.09 | 0.04 | **0.015** | 1.09 | 1.02 – 1.18 |
| Sex [female] | -0.27 | 0.04 | **<0.001** | 0.76 | 0.71 – 0.82 |
| Surgical procedure within 14 days prior to measurement [yes] | -0.40 | 0.04 | **<0.001** | 0.67 | 0.62 – 0.73 |
| **Random Effects** | | | | | |
| $\tau_{00}$ [variability in hospital intercepts] | | | 0.09 | | |
| N [hospitals] | | | 221 | | |
| Observations | | | 67336 | | |

Significant *p*-values are highlighted in bold.



For peer review only - http://bmjopen.bmj.com/site/about/guidelines.xhtml

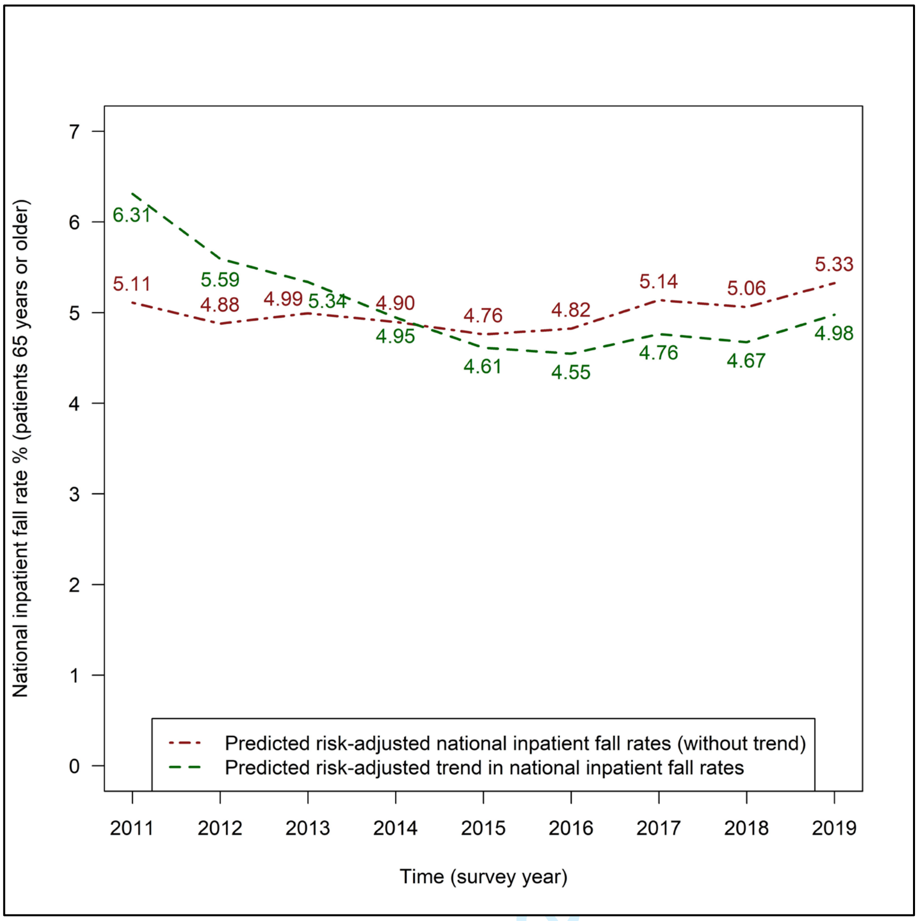


*Supplementary figure S3: Visualisation of predicted risk-adjusted (with and without time trend) Swiss national inpatient fall rates from 2011 to 2019, taking into account only patients 65 years old or older*

*Supplementary table S4: Illustration of the estimated non-linear trend using odds ratios, each of which describes the decrease in Swiss national fall rates in relation to the previous survey year, taking into account only patients 65 years old or older*

| **Survey year** | **2011/2012** | **2012/2013** | **2013/2014** | **2014/2015** | **2015/2016** | **2016/2017** | **2017/2018** | **2018/2019** |
|---|---|---|---|---|---|---|---|---|
| ***OR*** | 0.91 | 0.92 | 0.94 | 0.95 | 0.97 | 0.98 | 1.00 | 1.01 |
| **95% *CI*** | 0.86 – 0.96 | 0.89 – 0.96 | 0.91 – 0.96 | 0.93 – 0.97 | 0.95 – 0.98 | 0.96 – 1.00 | 0.96 – 1.03 | 0.97 – 1.06 |



For peer review only - http://bmjopen.bmj.com/site/about/guidelines.xhtml

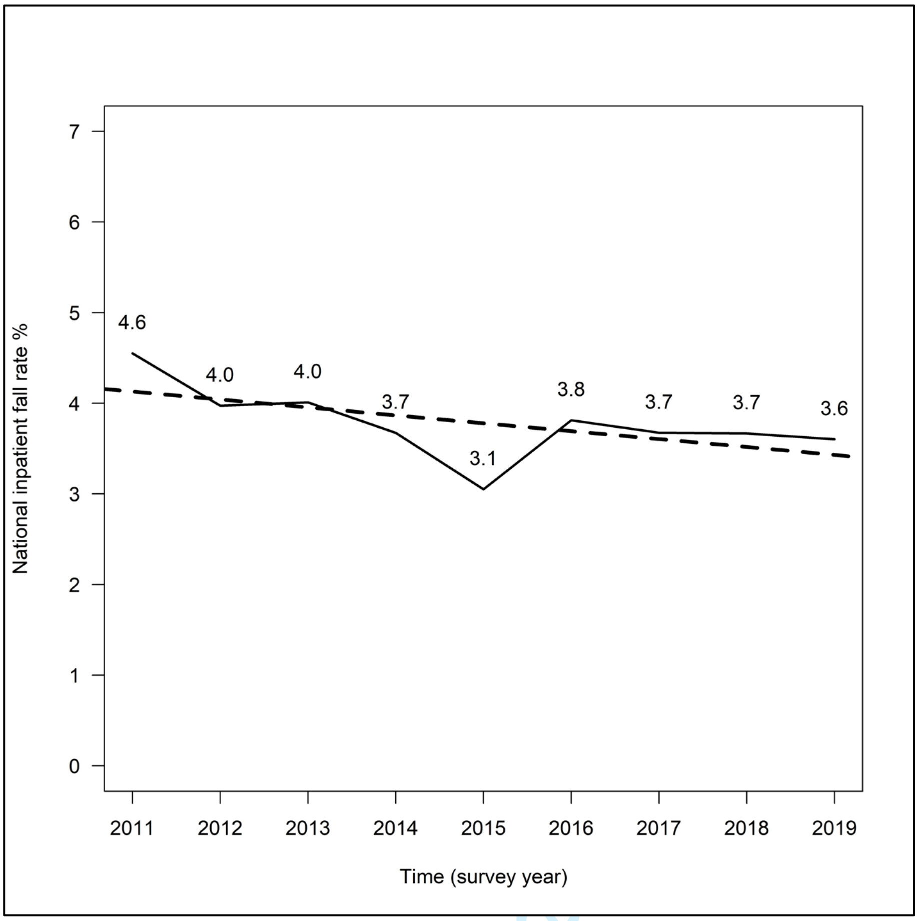


*Supplementary figure S4: Visualisation of the descriptive Swiss national inpatient fall rates from 2011 to 2019 and the estimated linear trend over time, taking into account only hospitals that provided data in all survey years*



For peer review only - http://bmjopen.bmj.com/site/about/guidelines.xhtml

*Supplementary table S5: Overview of the two-level random intercept logistic regression model used to derive the risk-adjusted trend in Swiss national inpatient fall rates, taking into account only hospitals that provided data in all survey years*

| | **Risk-adjusted trend in national inpatient fall rates** | | | | |
|---|---|---|---|---|---|
| *Predictors* | *coeff.* | *SE* | *p-value* | *OR* | *95% CI* |
| (Intercept) | -5.16 | 0.13 | **<0.001** | - | - |
| **Time related factors (trend)** | | | | | |
| Time | -0.05 | 0.01 | **<0.001** | - | - |
| **Patient related fall risk factors** | | | | | |
| Age [in years] | 0.01 | 0.00 | **<0.001** | 1.02 | 1.01 – 1.02 |
| CDS [care independent (70-75)] | Ref. | | | | |
| CDS [to a great extent independent (60-69)] | 1.04 | 0.06 | **<0.001** | 2.82 | 2.49 – 3.20 |
| CDS [partially dependent (45-59)] | 1.45 | 0.07 | **<0.001** | 4.25 | 3.73 – 4.84 |
| CDS [to a great extent dependent (25-44)] | 1.75 | 0.07 | **<0.001** | 5.76 | 4.99 – 6.66 |
| CDS [completely dependent (15-24)] | 1.28 | 0.11 | **<0.001** | 3.59 | 2.88 – 4.46 |
| ICD-10 – Mental and Behavioural disorders [yes] | 0.58 | 0.05 | **<0.001** | 1.79 | 1.64 – 1.96 |
| ICD-10 – Neoplasms [yes] | 0.35 | 0.05 | **<0.001** | 1.42 | 1.29 – 1.56 |
| ICD-10 – Diseases of the nervous system [yes] | 0.26 | 0.06 | **<0.001** | 1.30 | 1.16 – 1.45 |
| ICD-10 – Diseases of the blood and blood-forming organs [yes] | 0.22 | 0.05 | **<0.001** | 1.24 | 1.12 – 1.38 |
| ICD-10 – Endocrine, nutritional and metabolic diseases [yes] | 0.11 | 0.04 | **0.011** | 1.12 | 1.03 – 1.22 |
| ICD-10 – Diseases of the circulatory system [yes] | 0.12 | 0.05 | **0.021** | 1.12 | 1.02 – 1.24 |
| Sex [female] | -0.17 | 0.04 | **<0.001** | 0.85 | 0.78 – 0.92 |
| Surgical procedure within 14 days prior to measurement [yes] | -0.50 | 0.05 | **<0.001** | 0.60 | 0.55 – 0.67 |
| **Random Effects** | | | | | |
| $\tau_{00}$ [variability in hospital intercepts] | | | 0.06 | | |
| N [hospitals] | | | 93 | | |
| Observations | | | 64038 | | |

Significant *p*-values are highlighted in bold.

For peer review only



For peer review only - http://bmjopen.bmj.com/site/about/guidelines.xhtml

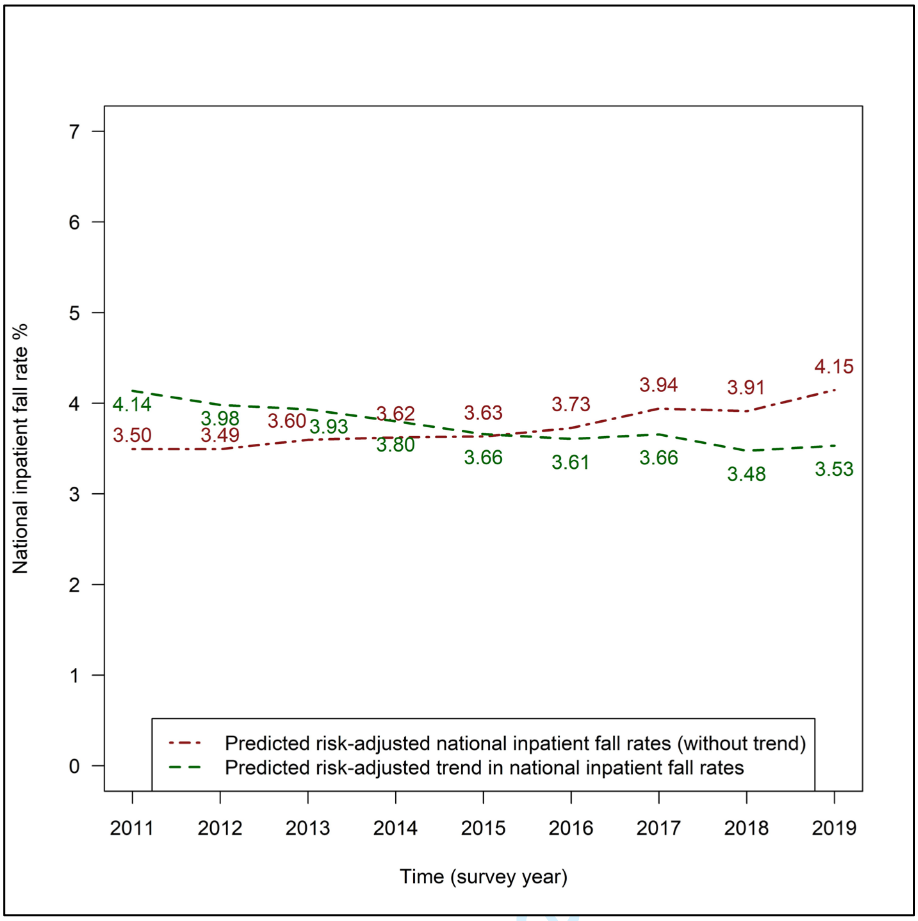


*Supplementary figure S5: Visualisation of predicted risk-adjusted (with and without time trend) Swiss national inpatient fall rates from 2011 to 2019, taking into account only hospitals that provided complete data in all survey years*



For peer review only - http://bmjopen.bmj.com/site/about/guidelines.xhtml

**STROBE 2007 (v4) Statement—Checklist of items that should be included in reports of *cross-sectional studies***

| Section/Topic | Item # | Recommendation | Reported on page # |
|---|---|---|---|
| **Title and abstract** | 1 | (*a*) Indicate the study's design with a commonly used term in the title or the abstract | 1 |
| | | (*b*) Provide in the abstract an informative and balanced summary of what was done and what was found | 2 |
| **Introduction** | | | |
| Background/rationale | 2 | Explain the scientific background and rationale for the investigation being reported | 5-7 |
| Objectives | 3 | State specific objectives, including any prespecified hypotheses | 7 |
| **Methods** | | | |
| Study design | 4 | Present key elements of study design early in the paper | 7 |
| Setting | 5 | Describe the setting, locations, and relevant dates, including periods of recruitment, exposure, follow-up, and data collection | 7-9 |
| Participants | 6 | (*a*) Give the eligibility criteria, and the sources and methods of selection of participants | 8-9 |
| Variables | 7 | Clearly define all outcomes, exposures, predictors, potential confounders, and effect modifiers. Give diagnostic criteria, if applicable | 9 |
| Data sources/ measurement | 8* | For each variable of interest, give sources of data and details of methods of assessment (measurement). Describe comparability of assessment methods if there is more than one group | 9 |
| Bias | 9 | Describe any efforts to address potential sources of bias | 8 |
| Study size | 10 | Explain how the study size was arrived at | 13 |
| Quantitative variables | 11 | Explain how quantitative variables were handled in the analyses. If applicable, describe which groupings were chosen and why | 10, 11 |
| Statistical methods | 12 | (*a*) Describe all statistical methods, including those used to control for confounding | 10-13 |
| | | (*b*) Describe any methods used to examine subgroups and interactions | 12 |
| | | (*c*) Explain how missing data were addressed | 13 |
| | | (*d*) If applicable, describe analytical methods taking account of sampling strategy | - |
| | | (*e*) Describe any sensitivity analyses | 12, 15 |
| **Results** | | | |

For peer review only

| | | | |
|---|---|---|---|
| Participants | 13* | (a) Report numbers of individuals at each stage of study—eg numbers potentially eligible, examined for eligibility, confirmed eligible, included in the study, completing follow-up, and analysed | 13 |
| | | (b) Give reasons for non-participation at each stage | 13 |
| | | (c) Consider use of a flow diagram | - |
| Descriptive data | 14* | (a) Give characteristics of study participants (eg demographic, clinical, social) and information on exposures and potential confounders | 13-14 |
| | | (b) Indicate number of participants with missing data for each variable of interest | 13 |
| Outcome data | 15* | Report numbers of outcome events or summary measures | 14-15 |
| Main results | 16 | (*a*) Give unadjusted estimates and, if applicable, confounder-adjusted estimates and their precision (eg, 95% confidence interval). Make clear which confounders were adjusted for and why they were included | 14-18 |
| | | (*b*) Report category boundaries when continuous variables were categorized | - |
| | | (*c*) If relevant, consider translating estimates of relative risk into absolute risk for a meaningful time period | - |
| Other analyses | 17 | Report other analyses done—eg analyses of subgroups and interactions, and sensitivity analyses | 18-19 |
| **Discussion** | | | |
| Key results | 18 | Summarise key results with reference to study objectives | 19-20 |
| Limitations | 19 | Discuss limitations of the study, taking into account sources of potential bias or imprecision. Discuss both direction and magnitude of any potential bias | 22 |
| Interpretation | 20 | Give a cautious overall interpretation of results considering objectives, limitations, multiplicity of analyses, results from similar studies, and other relevant evidence | 20-22 |
| Generalisability | 21 | Discuss the generalisability (external validity) of the study results | 22 |
| **Other information** | | | |
| Funding | 22 | Give the source of funding and the role of the funders for the present study and, if applicable, for the original study on which the present article is based | 32 |

*Give information separately for cases and controls in case-control studies and, if applicable, for exposed and unexposed groups in cohort and cross-sectional studies.

**Note:** An Explanation and Elaboration article discusses each checklist item and gives methodological background and published examples of transparent reporting. The STROBE checklist is best used in conjunction with this article (freely available on the Web sites of PLoS Medicine at http://www.plosmedicine.org/, Annals of Internal Medicine at http://www.annals.org/, and Epidemiology at http://www.epidem.com/). Information on the STROBE Initiative is available at www.strobe-statement.org.

For peer review only
